# Natural variation in MdNAC5 contributes to fruit firmness and ripening divergence in apple

**DOI:** 10.1093/hr/uhae284

**Published:** 2024-10-08

**Authors:** Li Liu, Yuanji Wang, Jianhua Guo, Ziqi Han, Kaixuan Yu, Yaxiao Song, Hongfei Chen, Hua Gao, Yazhou Yang, Zhengyang Zhao

**Affiliations:** State Key Laboratory of Crop Stress Biology for Arid Areas, College of Horticulture, Northwest A&F University, Yangling 712100, Shaanxi, China; College of Life Science, Northwest A&F University, Yangling 712100, Shaanxi, China; State Key Laboratory of Crop Stress Biology for Arid Areas, College of Horticulture, Northwest A&F University, Yangling 712100, Shaanxi, China; State Key Laboratory of Crop Stress Biology for Arid Areas, College of Horticulture, Northwest A&F University, Yangling 712100, Shaanxi, China; State Key Laboratory of Crop Stress Biology for Arid Areas, College of Horticulture, Northwest A&F University, Yangling 712100, Shaanxi, China; State Key Laboratory of Crop Stress Biology for Arid Areas, College of Horticulture, Northwest A&F University, Yangling 712100, Shaanxi, China; State Key Laboratory of Crop Stress Biology for Arid Areas, College of Horticulture, Northwest A&F University, Yangling 712100, Shaanxi, China; Department of Ecology and Evolutionary Biology, Yale University, New Haven, CT 06520, USA; State Key Laboratory of Crop Stress Biology for Arid Areas, College of Horticulture, Northwest A&F University, Yangling 712100, Shaanxi, China; State Key Laboratory of Crop Stress Biology for Arid Areas, College of Horticulture, Northwest A&F University, Yangling 712100, Shaanxi, China; State Key Laboratory of Crop Stress Biology for Arid Areas, College of Horticulture, Northwest A&F University, Yangling 712100, Shaanxi, China

## Abstract

Fruit firmness is an important trait for characterizing the quality and value of apple. It also serves as an indicator of fruit maturity, as it is a complex trait regulated by multiple genes. Resequencing techniques can be employed to elucidate variations in such complex fruit traits. Here, the whole genomes of 294 F_**1**_ hybrids of ‘Fuji’ and ‘Cripp's Pink’ were resequenced, and a high-density binmap was constructed using 5014 bin markers with a total map distance of 2213.23 cM and an average map distance of 0.44 cM. Quantitative trait loci (QTLs) of traits related to fruit were mapped, and an A-T allele variant identified in the coding region of *MdNAC5* was found to potentially regulate fruit firmness and ripening. The overexpression of *MdNAC5*^***A***^ resulted in higher production of methionine and 1-aminocyclopropanecarboxylic acid compared to *MdNAC5*^***T***^, leading to reduced fruit firmness and accelerated ripening in apples and tomatoes. Furthermore, the activities of MdNAC5^**A**^ and MdNAC5^**T**^ were enhanced through their differential binding to the promoter regions of *MdACS1* and *MdERF3*. Spatial variations in MdNAC5^**A**^ and MdNAC5^**T**^ caused changes in *MdACS1* expression following their interaction with MdERF3. Ultimately, utilizing different *MdNAC5* alleles offers a strategy to manipulate fruit firmness in apple breeding.

## Introduction

Apple (*Malus* × *domestica* Borkh.) is an important fruit in temperate regions of the world, with a global apple planting area and production of 4.82 million hectares and 93.92 million tons, respectively, in 2022. Apple fruit quality is a complex character controlled by multiple genes that affect traits such as fruit color, fruit texture, sugar content, acid content, and aroma [1, 2]. The fruit ripening process involves complex changes in texture, which is a key factor affecting fruit quality that has implications for the storability of fruit and its acceptance by consumers [3, 4]. Fruit firmness is an important index for measuring the internal quality of apple fruit that affects fruit palatability and determines the storage and transport capacity of fruit. There is still much room for improvement in fruit ripening traits, especially in fruit firmness. Analysis of the molecular mechanism underlying the regulation of fruit firmness and ripening is essential for improving the quality of apple plants in breeding programs [5]. However, identifying the genes that affect complex quality traits in apple is a major challenge because of the long juvenile period, perennial characteristics, and high genomic heterozygosity of apple plants [6–8].

Clarifying associations between complex quantitative traits such as fruit firmness using genetic linkage maps is an important approach for identifying closely linked molecular markers and major regulatory genes [9]. The construction of apple genetic map began with the “European Apple Genome Mapping Project” in 1989, and the first apple genetic map was constructed based on the theory of the “double pseudo test cross” [10, 11]. Simple sequence repeat (SSR) and expressed sequence tag-SSR (EST-SSR) markers were used to construct the earliest genetic maps [12–15]. The development of second-generation sequencing technology facilitated the construction of genetic maps of apple plants [16–19], and the combined use of SSR and SNP markers has significantly increased the accuracy of genetic maps [20–22]. Bulk segregation analysis (BSA) is a quantitative trait locus (QTLs) mapping strategy based on next-generation sequencing (NGS) that has been widely used for the construction of genetic maps for various grains, vegetables, and fruits [23–28]. Previous studies have shown that MapQTL combined with BSA-Seq can be used to identify early flowering tolerance genes in cucumber, heat tolerance genes in lettuce, and QTLs related to high acidity in apple fruits [29, 30]. Genome-wide association studies (GWAS) can be used to map the QTLs of traits in natural populations, and they can detect multiple alleles at a single locus, as well as candidate genes with higher accuracy [3, 31–36]. Based on the above mapping strategies for complex fruit traits, several QTLs related to fruit firmness and ripening have been identified on chromosomes 3, 5, 7, 10, 14, 15, and 16, and relevant candidate genes identified include *MdACO1*, *MdACS1*, *MdEXP7*, *MdPG1*, *MdERF4, MdERF118,* and *NAC18.1* [35–41].

Changes in fruit firmness are some of the most obvious changes in fruit during the ripening process, and fruit firmness is not only affected by changes in physical properties, such as cell wall structure [42], the stratum corneum, and cell swelling but also by ethylene and transcription factors [43–46]. Ethylene is synthesized from methionine (Met). In this process, Met is converted to *S*-adenosylmethionine (SAM) by *S*-adenosylmethionine synthetase and adenosine triphosphate. SAM is then converted to 1-aminocyclopropanecarboxylic acid (ACC) by 1-aminocyclopropanecarboxylic acid synthase (ACS). Finally, ACC is directly oxidized by 1-aminocyclopropanecarboxylic acid oxidase (ACO) to form ethylene [47, 48]. As a climacteric fruit apple, it will produce a large amount of ethylene during the ripening [49]. The ethylene response factor MdERF3 binds to the *MdACS1* promoter and activates its transcription to promote the release of ethylene, and MdERF2 binds to the *MdERF3* promoter and negatively feeds back to regulate ethylene production [46]. Mutation of *SlACS2*, an ethylene biosynthesis gene, significantly delays the ripening of tomato [50]. Mutation of the ethylene response factor-associated amphiphilic inhibition (EAR) motif in the coding region of MdERF4 induces a change in the binding of MdERF4 to the *MdERF3* promoter, thus inhibiting ethylene production and the loss of fruit firmness [38]. In addition, genes involved in the ethylene pathway, such as *MdBBX25* [51], *MdCRF4* [50], *MdERF3/MdERF118* [41], *MdMYC2* [49], and *MdPUB29-MdbHLH3* [45], promote or inhibit ethylene production and affect fruit firmness and ripening in apple.

The NAC transcription factor family is one of the biggest families of plant-specific transcription variables, and individuals of this family play a key part in the organic handle of fruit growth, development, and ripening [52]. Many studies have examined the roles of NAC factors in regulating fruit firmness and ripening in fruit, including SlNOR [53, 54], SlNOR-like1 [55], and SlNAM1 [56] from tomato; AdNAC6 and AdNAC7 [56] from kiwifruit; FcNAC [57] and FaNAC035 [58] from strawberry; MaNAC2 from banana [59]; CmNAC-NOR from melon [60]; and PpNAC1 and PpNAC5 from peach [61, 62]. However, few studies of key regulatory genes in apple have been conducted; ethylene has been shown to enhance MdMAPK3-mediated MdNAC72 phosphorylation to promote fruit softening [63].

The QTLs mapping resolution based on genetic map or GWAS depends on the marker density and population size [31]. With the progress of NGS, the revolution of genome analysis and its application in the trait improvement has been promoted. Resequencing strategies have been increasingly used to clarify the relationship between variation in key genes and fruit traits. Here, the whole genomes of F_1_ hybrids of ‘Fuji’ and ‘Cripp's Pink’ were resequenced, and this dataset was used to construct a high-density binmap. QTLs of traits related to fruit firmness were mapped, and an A-T allele variant in the coding region of *MdNAC5* in one of the QTLs with the largest logarithm of odds (LOD) and variance explained was identified as a candidate gene controlling fruit firmness and ripening. The homozygous genotypes MdNAC5^A^ and MdNAC5^T^ with natural mutations might target the *MdERF3* and *MdACS1* complex to differentially regulate fruit firmness through the ethylene pathway.

## Results

### Resequencing of the hybrid population

**Figure 1:**
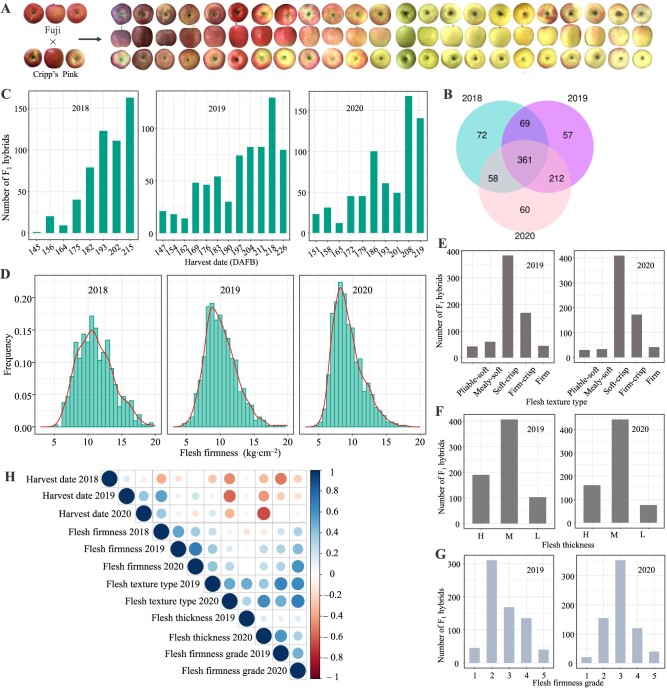
Distributions of and correlations among fruit-related traits. (**A**) Example hybrids of ‘Fuji’ and ‘Cripp's Pink’. (**B**) The number of F_1_ hybrids of ‘Fuji’ and ‘Cripp's Pink’ for three consecutive years (from 2018 to 2020). (**C, D**) Distribution of HD and FF traits in 2018–2020. (**E–G**) Distributions of flesh texture type, flesh thickness, and flesh firmness grade traits in 2019 and 2020. H, M, and L indicate that the flesh thickness was high, moderate, and low, respectively. A scale of 1 to 5 was used to evaluate FF, with 1 being soft and 5 being firm. (**H**) Heat maps of the correlation analysis among six traits and years.

Stable genetic F_1_ hybrids (*n* = 935) were generated by crossing ‘Fuji’ and ‘Cripp's Pink’ (Fig. 1A). A total of 560, 699, and 717 F_1_ hybrids of ‘Fuji’ and ‘Cripp's Pink’ populations were investigated in 2018, 2019, and 2020, respectively (Fig. 1B). Data on fruit firmness-related traits that were taken included harvest date (HD) and flesh firmness (FF); data for the sensory evaluation included flesh texture type, flesh thickness, and flesh firmness grade. Shapiro–Wilk analysis showed that all traits were normally distributed and varied quantitatively (Fig. 1C–G). Analysis of correlations among traits showed that the HD was positively correlated with FF and negatively correlated with flesh texture type (Fig. 1H). There was a strong correlation between fruit firmness measured by the instrument and fruit texture according to the sensory evaluation, indicating that the phenotypic data were robust. The broadsense heritability of HD and FF ranged from 99.49% to 111.26%, (Table S1). Our results revealed natural variation in all the traits in F_1_ hybrids.

To clarify the genetic basis of fruit quality control, 294 offspring were randomly selected from the F_1_ hybrid population of ‘Fuji’ and ‘Cripp's Pink’, and then a genetic map was constructed. The raw data were filtered and evaluated using high-throughput DNA resequencing, and finally a total of 3011.01 GB of clean data were obtained; the GC content and Q30 were 38.59% and 90.39%, respectively (Tables S2 and S3). The female parent ‘Fuji’ and the male parent ‘Cripp's Pink’ had 180 983 278 and 147 985 987 paired-end reads; the average sequencing depth was 13.09×; the average sequencing depth for ‘Fuji’, ‘Cripp's Pink’, and each the F_1_ hybrids were 74×, 60×, and 12.73×, respectively. We used the base mass, base ratio, insert size, and coverage depth distribution to evaluate the quality of the resequencing dataset of the ‘Fuji’ and ‘Cripp's Pink’ parents (Fig. S1). Among the parental SNPs, the transition number (Ti) was 2 549 656, and the transversion number (Tv) was 1 219 255 (Ti/Tv: 2.09, Table S4). According to analysis of the clean reads in the reference genome, the mapping rates of ‘Fuji’, ‘Cripp's Pink’, and the F_1_ hybrids were 97.56%, 97.38%, and 96.71%, respectively (Table S5).

### Construction of a high-density genetic binmap

**Figure 2:**
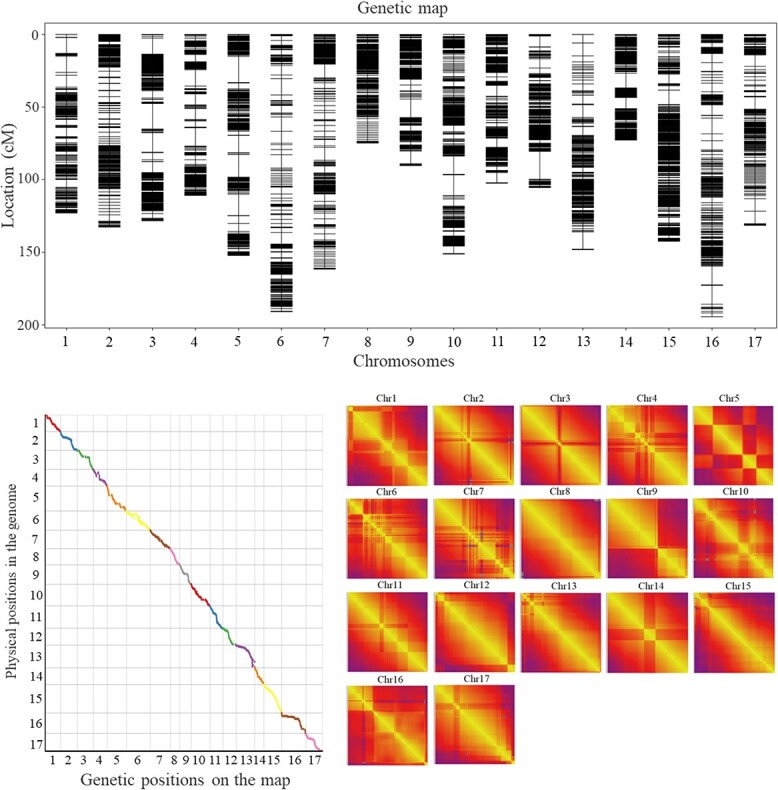
High-density genetic linkage map based on 5014 bin markers of ‘Fuji’ × ‘Cripp's Pink’. (**A**) The dispersion of bin markers on the 17 chromosomes. (**B**) The Spearman correlation coefficients of markers on the map between genetic positions and physical positions of reference genome. (**C**) The heat maps show distances and recombination rates between markers on the 17 chromosomes. The yellow to purple indicates the recombination rate. Gray indicates a lack of recombination rate between the markers that only segregated in different parents.

A total of 5014 bin markers were developed and divided on 17 chromosomes to construct the genetic map of the female ‘Fuji’ parent. The total genetic map distance was 2194.84 cM, and the chromosome length ranged from 60.81 to 240.68 cM (Fig. S2A; Table S6A). An addition of 4880 bin markers was created for the genetic map of the male ‘Cripp's Pink’ parent, with an addition of genetic map distance of 2219.79 cM and chromosome lengths extending from 80.37 to 222.09 cM (Fig. S2B and Table S6B). Concurring with the collinear loci on each chromosome, the genetic map of the parents containing 5014 bin markers was obtained (Fig. 2A and Table 1). The entire length of the genetic map was 2213.23 cM, and the average genetic distance was 0.44 cM. The length of the 17 chromosomes extended from 72.78 cM (chr14) to 194.37 cM (chr16), with a greatest span of 121.59 cM and an average length of 130.19 cM. The number of bin markers on 17 chromosomes shifted from 241 (chr4, average distance of 0.46 cM) to 435 (chr15, average distance of 0.33 cM), and the average number of bin markers was 295. The shortest and longest chromosomes were chr14 and chr16, which contained 265 and 313 bin markers with genetic map distances of 72.78 cM and 194.37 cM and average distances of 0.27 cM and 0.62 cM, respectively. The genetic gap ranged from 19.19 cM for chr6 to 2.39 cM for chr8.

**Table 1:** Description of the basic characteristics of the 17 chromosomes

Chromosome	Total bin markers	Total distance (cM)	Average distance (cM)	Max gap (cM)	Gaps <5 cM (%)
Chr1	267	122.95	0.46	11.03	98.12
Chr2	307	132.63	0.43	4.57	100.00
Chr3	294	128.35	0.44	17.50	98.63
Chr4	241	110.88	0.46	12.52	98.33
Chr5	316	152.24	0.48	17.98	98.10
Chr6	268	190.96	0.71	11.33	97.38
Chr7	293	161.61	0.55	9.38	98.97
Chr8	260	74.80	0.29	2.39	100.00
Chr9	242	90.39	0.37	12.73	99.17
Chr10	372	151.23	0.41	14.32	99.19
Chr11	306	102.45	0.33	7.42	99.02
Chr12	266	105.47	0.40	19.19	98.87
Chr13	305	148.22	0.49	11.88	98.03
Chr14	265	72.78	0.27	10.00	99.24
Chr15	435	142.43	0.33	9.86	99.54
Chr16	313	194.37	0.62	12.85	97.44
Chr17	264	131.47	0.50	8.60	98.48
Total	5014	2213.23	0.44	19.19	98.74

The positions of the 5014 bin markers on the genetic map were collinear with the reference genome, and the results are shown in Fig. 2B. The Spearman correlation coefficients between each chromosome on a physical graph suggested that chr1, chr3, chr5, chr8, chr9, chr12, chr14, chr15, and chr16 were correlated with 1, and the correlations of the other chromosomes were very close to 1. Pairwise recombination fractions among all 17 chromosomes were located on the diagonal of the heat map, indicating that the genetic map was of high quality (Fig. 2B and Fig. S3). In this study, the frequency of double exchanges of the 17 linkage groups was less than 3%, indicating that the type and order of markers in the map were appropriate (Fig. S4).

### QTL analysis and identification of genes involved in apple fruit firmness and ripening

The interval mapping method was used to identify QTLs for fruit-related traits in apple, and a total of 60 putatively related QTLs were identified (Fig. S5; Table S7). The LOD score and physical length of these identified QTLs ranged from 2.64 Mb to 30.30 Mb and from 0.01 Kb to 23.02 Mb, respectively. The QTLs for HD and FF and the mapped QTLs partially overlapped with genes previously identified to be involved in fruit firmness on chr3, chr10, chr15, and chr16. In addition, we identified major QTLs on chr2, chr7, and chr9 for fruit firmness and ripening period. These results demonstrated the high accuracy and quality of the genetic map. We identified 8 major QTLs for flesh texture type, 10 major QTLs for flesh thickness, and 9 major QTLs for flesh firmness grade on chr3, chr4, chr5, chr6, chr7, chr9, chr15, and chr16. Among the 60 QTLs, the highest LOD score of 30.30 for harvest time in 2019 was observed for chr3 (explaining 42.80% of the variation), and the maximum LOD score of 13.17 for fruit firmness in 2018 was observed for chr3 (explaining 26.30% of the variation). In the sensory evaluation of the three traits related to fruit firmness, the highest LOD score was observed for chr3.

Based on these findings, we further re-sequenced ‘Fuji’ × ‘Cripp's Pink’ hybrids with extreme firmness (50 plants) (excluding plants used to construct the genetic map) and performed BSA-Seq. The phenotypic distribution of the average firmness of fruit in the 3-year pool of R02 (firm) and R03 (soft) F_1_ offspring is shown in Fig. S6A. The raw data obtained from the high-throughput DNA re-sequencing were filtered and analyzed (Fig. S6B–F). The GC content of the two pools (R02/R03) was greater than 38%, and 294 864 360 and 270 058 776 clean paired-end reads were obtained for R02 and R03, respectively (Tables S8 and S9). Finally, 1 874 945 high-quality SNP loci were obtained in the mixed pool of ‘Fuji’ × ‘Cripp's Pink’ (Table S10). The Euclidean distance (ED) and Δ(SNP-index) values of the BSA-Seq data revealed 9 QTLs related to fruit firmness on chr3, chr5, and chr15 (Fig. S7A; Table S11). The locus chr3:30277722.0.30924873 was located in the interval on chr3.

**Figure 3:**
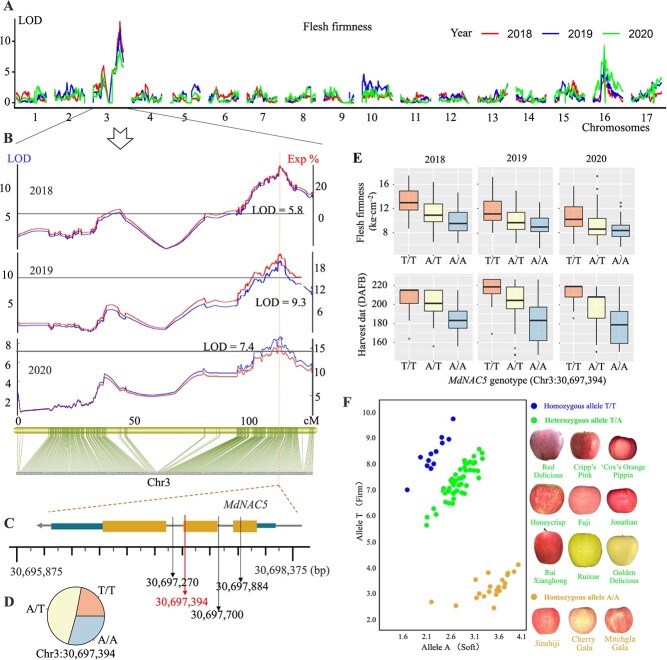
*MdNAC5* was selected by QTLs mapping and affected fruit firmness and ripening. (**A**) LOD values of 17 chromosomes for the 3 years according to interval mapping. (**B**) Stable QTLs for FF in chr3. (**C**) Location of *MdNAC5* associated with fruit FF. The black arrows indicate the SNPs related to FF and HD in *MdNAC5*. The red arrow indicates the most significant and nonsynonymous SNP. (**D**) Frequency of the SNP genotypes of *MdNAC5* in 294 F_1_ hybrids. (**E**) Relationship between fruit FF and HD phenotypes of 294 F_1_ hybrids and genotypes of *MdNAC5* at locus Chr3:30697394. (**F**) **C**ompetitive allele-specific PCR show that the allele mutation at Chr3:30697394 in *MdNAC5* could eliminate the extremely firm or soft varieties, and most of the varieties are A/T heterozygous for *MdNAC5*.

Annotations of genes in the chr3:30277722.0.30924873 interval are shown in Table S12. The annotations suggested that these genes play important roles in redox reactions, membrane composition, and transport. Transcriptome data [64] from ‘Fuji’ and ‘Cripp's Pink’ during fruit development at 120, 150, 170, 180, 190, and 200 days after full bloom (DAFB) revealed five differentially expressed genes (DEGs), with the expression of MD03G1222600 (designated as *MdNAC5*) being the highest at each stage (Fig. S7B). Analysis of tissue-specific expression patterns revealed that the expression of *MdNAC5* varied among tissues (leaf, root, stem tip, flower, and young fruit tissue); specifically, its expression was highest in flowers, followed by young fruit and stem tips (Fig. S8A). *MdNAC5*, which was located on chr3, explained the most variance in fruit-related traits and had the largest LOD value according to the mapping results (Fig. 3A, B; Table S7). Analysis of the resequencing data revealed four variant SNP sites in *MdNAC5*. Two variable sites (chr3:30697270, chr3:30697700) were located in the intron region, and two variable sites were located in the coding region (chr3:30697394, chr3:30697884, Fig. 3C). The mutation of chr3:30697394 is non-synonymous, whereas that of chr3:30697884 is synonymous. A non-synonymous SNP (A-to-T) in *MdNAC5*, a homolog of tomato *NOR*, results in the substitution of the encoded amino acid from methionine (Met) to leucine (Leu).

The SNP genotyping and the expression of *MdNAC5* in hybrids showed that the A/A allele (*MdNAC5^A^*) is related to the early ripening and softening of fruit, and the T/T allele (*MdNAC5^T^*) is related to the late ripening and hardening of fruit (Figs 3D, E and S8B). The hybrids of the homozygous genotype *MdNAC5^A^* had lower fruit firmness and higher gene expression, whereas the hybrids of the homozygous genotype *MdNAC5^T^* had lower gene expression (Fig. S8B). The results of subcellular localization analysis of tobacco showed that both MdNAC5^A^ and MdNAC5^T^ proteins played a role in the nucleus (Fig. S8C). Furthermore, competitive allele-specific PCR showed that the allele mutation at Chr3:30697394 in *MdNAC5* could eliminate the extremely firm or soft varieties, and most of the varieties in production were A/T heterozygous for *MdNAC5* (Fig. 3F). These results suggest that the A-T allele variant in the coding region of *MdNAC5* might be an important factor leading to variation in fruit firmness.

### Characteristics and transcriptome analysis of *MdNAC5* in apple

**Figure 4:**
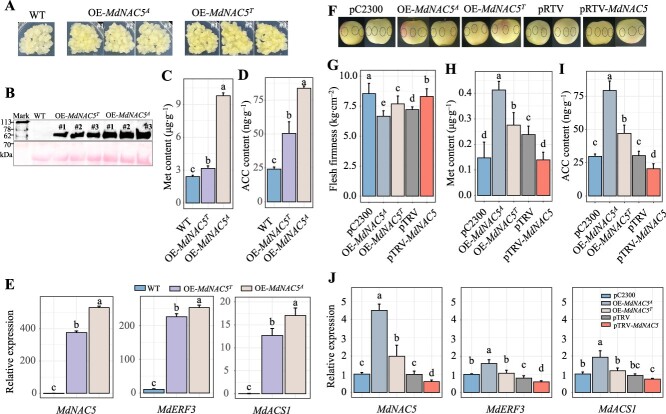
Expression analysis and characterization of *MdNAC5^A^* and *MdNAC5^T^* in apple. (**A**) Phenotype of ‘Orin’ calli overexpressing *MdNAC5^A^*#1/2/3 and *MdNAC5^T^*#1/2/3. (**B**) The protein levels of *MdNAC5* transgenic apple calli. (**C, D**) Content of Met and ACC in *MdNAC5* transgenic apple calli. (**E**) Relative expression of *MdNAC5*, *MdERF3*, and *MdACS1* in the transcriptome of MdNAC5 transgenic apple calli. (**F**) Transient overexpression of *MdNAC5* in ‘Fuji’ apple. Flesh firmness (**G**) and content of Met (**H**) and ACC (**I**) in *MdNAC5* transient transformed apple fruit. (**J**) Relative expression of *MdNAC5*, *MdERF3*, and *MdACS1* in *MdNAC5* transgenic apple fruits. The bars are mean the values of three replicates (± SD), and diverse letters show differ significantly (Turkey’s test, *P* < 0.05).

To examine the role of *MdNAC5^A^* and *MdNAC5^T^* in regulating fruit firmness, the recombinant plasmids pC2300-*MdNAC5^A^* and pC2300-*MdNAC5^T^* (for overexpression) were introduced into apple calli (Fig. 4A). The protein expression level of *MdNAC5* transgenic apple calli is shown in Fig. 4B. We analyzed the expression of wild-type (WT), *MdNAC5^A^*-overexpression (OE-*MdNAC5^A^*, L), and *MdNAC5^T^*-overexpression (OE-*MdNAC5^T^*, M) lines via RNA sequencing. The transcriptome data were robust based on the high Pearson correlation coefficient and the similarity in the biological replicates in the WT, L, and M groups (Fig. S9A). RNA-seq data analysis revealed 6986, 6229, and 2473 significantly upregulated genes and 5145, 3573, and 1408 significantly downregulated genes in the WT vs. L, WT vs. M, and L vs. M groups, respectively (Fig. S9B). Gene ontology (GO) analysis of these DEGs revealed various functions in the cellular component, molecular function, and biological process categories (Fig. S9C–F). This is consistent with our speculation that mutation of the *MdNAC5* allele affects ethylene synthesis and regulation, given that this mutation results in a change in the amino acid at the variable site from methionine to leucine. This suggests that *MdNAC5* is involved in ethylene synthesis, and variation in the alleles in the coding region is an important factor underlying differences in fruit firmness and ripening. The stable overexpression of these alleles (OE-*MdNAC5^A^* and OE-*MdNAC5^T^*) led to the content of Met and ACC increasing significantly in apple calli, and the Met and ACC contents in OE-*MdNAC5^A^* were significantly higher than those in OE-*MdNAC5^T^* (Fig. 4C, D). Transcriptome analysis revealed that overexpression of *MdNAC5^A^* and *MdNAC5^T^* significantly increased their expression and up-regulated the expression of various ethylene pathway-related genes, including *MdACS1* and *MdERF3* (Fig. 4E). By contrast, the expression of ethylene-related genes was up-regulated to a greater degree in OE-*MdNAC5^A^* than in OE-*MdNAC5^T^*.

**Figure 5:**
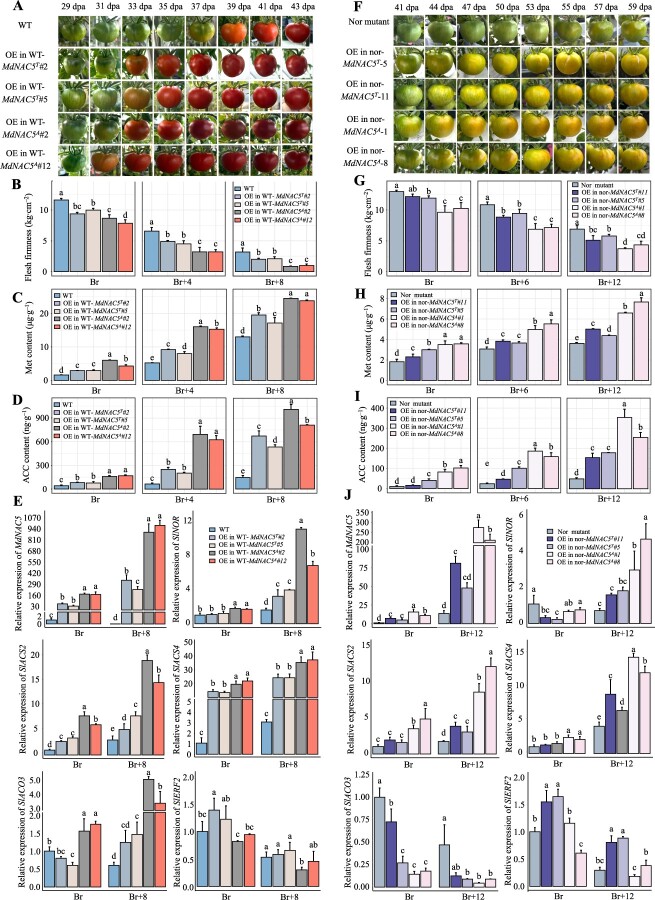
Analysis of the functional effects of overexpressing *MdNAC5^A^* and *MdNAC5^T^* in tomato. Phenotypes of wild-type (**A**) and nor mutant (**F**) cv. Ailsa Craig tomato overexpressing *MdNAC5^A^* and *MdNAC5^T^*. dpa, day post-anthesis. Flesh firmness (**B, G**), content of Met (**C, H**) and ACC (**D, I**), and expression levels of ripening-related genes (e, j) in transgenic and WT fruits at the indicated stages. Br represent breaker and Br + n represent the days after breaker. The bars are mean the values of three replicates (± SD), and diverse letters show differ significantly (Turkey’s test, *P* < 0.05).

**Figure 6:**
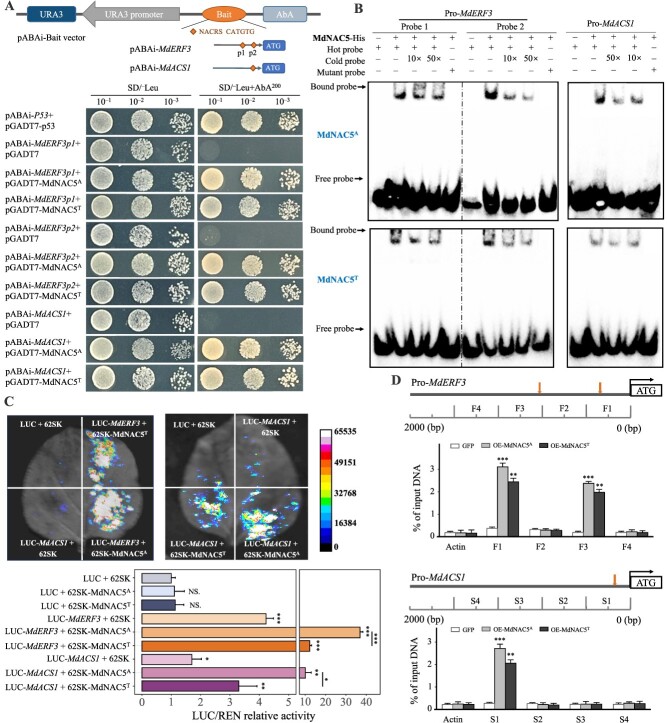
MdNAC5 regulates the expression of *MdERF3* and *MdACS1* by binding to their promoters. (**A**) Y1H assay showing that MdNAC5^A^ and MdNAC5^T^ bind to the *MdERF3* and *MdACS1* promoters. The schematic diagram of truncated 350-bp fragments with NACRS elements on *MdERF3* and *MdACS1* promoters. The fusion vectors pGADT7-MdNAC5^A^/MdNAC5^T^ and pABAi-*MdERF3*/*MdACS1* were cotransformed in Y1H on the SD/−Leu (−LT) medium. Single colonies were dissolved in 6 μl of sterile 0.1% NaCl, diluted to 10^−1^ to 10^−3^, and finally transferred to the SD/−Leu/AbA^200^ medium. (**B**) EMSA showing that MdNAC5^A^ and MdNAC5^T^ bind to the promoters of *MdERF3* and *MdACS1*. The shifted probes and free DNA probes are indicated by black arrows. “+” and “–” demonstrate the presence and absence of the protein and the probe appeared, individually. (**C**) Dual-luciferase assay revealing that MdNAC5^A^ and MdNAC5^T^ bind to the *MdERF3* and *MdACS1* promoters. The white color indicates high luciferase activity, and the black color indicates low luciferase activity. The bar chart shows the relative activity of LUC/REN and set at least three repetitions. (**D**) CHIP-qPCR assay showing that MdNAC5^A^ and MdNAC5^T^ bind to the *MdERF3* and *MdACS1* promoters. F1–F4 indicate the position of qPCR detection for the *MdERF3* promoter, and S1–S4 indicate the position of qPCR detection for the *MdACS1* promoter. Actin serves as an internal control. Values are means of three replicates ± SD (Turkey’s test, ^***^P < 0.001, ^**^  *P* < 0.01, ^*^P < 0.05).

Transient overexpression of *MdNAC5* and silencing of this gene via VIGS were performed in ‘Fuji’ apple fruit at 165 DAFB (Fig. 4F). The fruit firmness was lower, the content of Met and ACC was enhanced, and the expression of genes involved in the ethylene pathway (*MdACS1* and *MdERF3*) and self-expression were significantly greater in apple plants overexpressing *MdNAC5^A^* and *MdNAC5^T^* than in control plants (Fig. 4G–I). The fruit firmness of *MdNAC5^A^* was lower than that of *MdNAC5^T^*; however, the content of Met and ACC and the expression of *MdACS1* and *MdERF3* were higher in *MdNAC5^A^* than in *MdNAC5^T^*. The content of Met and ACC and the expression of *MdACS1/MdERF3* were lower in *MdNAC5*-silenced fruits than in control plants (Fig. 4J). These data indicated that the function of *MdNAC5^A^* and *MdNAC5^T^* in regulating the firmness of apple fruits and calli was highly dependent on ethylene pathway-related genes, and the alteration in the content of Met and ACC and gene expression was greater in *MdNAC5^A^* than in *MdNAC5^T^*.

### Effects of *MdNAC5^A^* and *MdNAC5^T^* overexpression on ethylene biosynthesis-related substances and gene expression in tomato fruit

To further clarify the role of the two homozygous genotypes *MdNAC5^A^* and *MdNAC5^T^* in fruit ripening, we fused *MdNAC5^A^* and *MdNAC5^T^* to the pC2300 vector and overexpressed *MdNAC5^A^* and *MdNAC5^T^* in tomato (Ailsa Craig, WT; AC tomato with *SlNOR* deletion mutants, Nor; Fig. S10A). A total of 32 independent T_0_ transgenic lines were obtained, and gene expression levels were determined (Fig. S10B, C). Two representative transgenic lines (WT-*MdNAC5^T^*#2/5, WT-*MdNAC5^A^*#2/12, nor-*MdNAC5^T^*#5/11, and nor-*MdNAC5^A^*#1/8) were used for the detection of protein levels and subsequent analysis (Fig. S10d). To evaluate the fruit ripening phenotype of transgenic lines overexpressing *MdNAC5^A^* and *MdNAC5^T^*, the fruit ripening process of transgenic lines, WT, and nor mutants were studied. The fruit ripening of WT-*MdNAC5^T^*#2/5 and WT-*MdNAC5^A^*#2/12 lines was promoted, and fruits reached the breaker (Br) stage approximately 5 and 7 days earlier compared with WT plants, respectively (Fig. 5A). The fruit firmness of transgenic lines overexpressing WT-*MdNAC5^T^*#2/5 and WT-*MdNAC5^A^*#2/12 was significantly lower than that of WT plants, and the content of Met and ACC in transgenic fruits of WT-*MdNAC5^A^*#2/12 at the Br, Br + 4 (4 days after breaker), and Br + 8 stage increased gradually and was significantly higher in the fruits of WT-*MdNAC5^A^*#2/12 than in the fruits of WT-*MdNAC5^T^*#2/5 (Fig. 5B, C). Overexpression of nor-*MdNAC5^T^*#5/11 and nor-*MdNAC5^A^*#1/8 significantly accelerated the ripening process of tomato, but the ripening phenotype of the nor mutant fruit could not be completely restored; the fruit reached the breaker stage approximately 12 and 16 days earlier for WT-*MdNAC5^A^*#2/12 and WT-*MdNAC5^T^*#2/5 compared with the nor mutant, respectively (Fig. 5F). In addition, the fruit firmness of transgenic lines overexpressing nor-*MdNAC5^T^*#5/11 and nor-*MdNAC5^A^*#1/8 was significantly lower than that of the nor mutant, while the content of Met and ACC was significantly higher in transgenic fruits than in the nor mutants at Br, Br + 6, and Br + 12 (Fig. 5G–I). The expression of maturation-related genes, including *MdNAC5*, *SlNOR*, *SlACS2*, *SlACS4*, and *SlACO3*, was significantly higher in transgenic lines (WT-*MdNAC5^T^*#2/5, WT-*MdNAC5^A^*#2/12, nor-*MdNAC5^T^*#5/11, and nor-*MdNAC5^A^*#1/8) than in WT plants and nor mutants (Fig. 5E, J). Fruit ripening was promoted to a greater degree in OE-*MdNAC5^A^* tomato plants than in OE-*MdNAC5^T^* plants. In sum, these results show that the regulatory effects of *MdNAC5^A^* and *MdNAC5^T^* on tomato and apple fruit ripening were similar.

### MdNAC5^A^ and MdNAC5^T^ differentially regulate the expression of *MdERF3* and *MdACS1* by binding to their promoters

Previous studies have shown that MdERF3 is a potential positive regulatory transcription factor, which promotes the expression of *MdACS1*. The relationships of MdNAC5^A^ and MdNAC5^T^ with *MdERF3/MdACS1* in this study were evaluated using Y1H, dual-luciferase, and electrophoretic mobility shift assays (EMSAs). *MdERF3* and *MdACS1* promoters were truncated into 300-bp fragments with NACRS elements and inserted into pABAi vectors to form pABAi-*MdERF3p1*, pABAi-*MdERF3p2*, and pABAi-*MdACS1* fusion expression vectors. The fusion expression vector and pGADT7-MdNAC5^A^ and pGADT7-MdNAC5^T^ were cotransformed into yeast-competent cells. The transformant with truncated promoter containing the NACRS element grew well on SD/−Leu with 200 ng∙ml^−1^ AbA, which indicated that MdNAC5^A^ and MdNAC5^T^ could differentially bind to the NACRS element of the *MdERF3* and *MdACS1* promoters in yeast (Figs 6a and S11). Next, MdNAC5^A^ and MdNAC5^T^ were fused to pET32a vector for protein induction and purification, and the interactions of MdNAC5^A^-His and MdNAC5^T^-His with pro-*MdERF3/MdACS1* were analyzed using EMSAs. EMSAs showed that MdNAC5^A^-His and MdNAC5^T^-His could bind to the promoter of pro-*MdERF3/MdACS1* (including the NACRS element), and the binding strength of MdNAC5^A^-His to the NACRS element on pro-*MdERF3/MdACS1* was greater than that of MdNAC5^T^-His (Fig. 6B). Dual-luciferase assays showed that MdNAC5^A^ and MdNAC5^T^ bind to the *MdERF3* and *MdACS1* promoters in tobacco leaves. The detection of luciferase LUC/REN relative activity *in vivo* showed that the co-expression of LUC-*MdERF3/MdACS1* and 62SK-MdNAC5^A^/MdNAC5^T^ was significantly higher in tobacco leaves compared with other combinations, and the luciferase activity was greater in the LUC-*MdERF3/MdACS1* and 62SK-MdNAC5^A^ combination than in LUC-*MdERF3/MdACS1* and 62SK-MdNAC5^T^ (Fig. 6C). Chromatin immunoprecipitation qPCR (CHIP-qPCR) analysis was performed to determine whether MdNAC5 can directly bind to these motifs. The results showed that MdNAC5^A^ and MdNAC5^T^ could bind to the fragments (F1, F3, and S1) containing NACRS elements in *MdERF3* and *MdACS1* promoters (Fig. 6D). These results indicate that MdNAC5^A^ and MdNAC5^T^ activate the expression of *MdERF3/MdACS1* by binding to the *cis*-element of NACRS on the *MdERF3/MdACS1* promoter, and the positive regulatory effects of MdNAC5^A^ were stronger than those of MdNAC5^T^.

### The interactions of MdNAC5^A^ and MdNAC5^T^ with MdERF3 regulate the expression of *MdACS1* in differentiation

**Figure 7:**
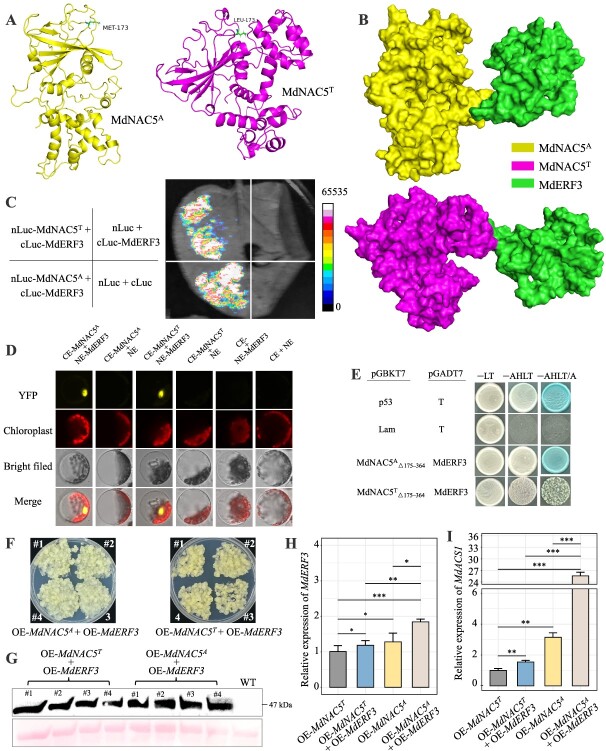
MdNAC5^A^ and MdNAC5^T^ differentially interact with MdERF3 to regulate *MdACS1*. (**A**) The structure of MdNAC5^A^ and MdNAC5^T^. (**B**) Docking prediction of MdNAC5^A^ and MdNAC5^T^ on MdERF3. (**C**) Luciferase complementation imaging assays showed that MdNAC5^A^ and MdNAC5^T^ interact with MdERF3. (**D**) BiFC assays showed that MdNAC5^A^ and MdNAC5^T^ interact with MdERF3 (indicated by the fluorescence observed in the nucleus of protoplasts). The YFP fluorescence coexpressed by MdNAC5^A^/MdNAC5^T^ and MdERF3 *Arabidopsis thaliana* protoplasts was detected by confocal laser scanning microscope. From left to right, YFP, chloroplast, bright field, and merged field images. Bars = 10 μm. (**E**) Y2H assays showing that MdNAC5^A^ and MdNAC5^T^ interact with MdERF3. The fusion vectors pGBKT7-MdNAC5^A^_△175–364_/MdNAC5^T^_△175–364_ and pGADT7-MdERF3 were co-transformed in Y2H on SD/−Leu/−Trp (−LT) medium. Single colonies were dissolved in 10 μl of sterile 0.1% NaCl, coated, and transferred to SD/−Ade/–His/−Leu/−Trp/X-α-Gal (–AHLT) and SD/−Ade/–His/−Leu/−Trp/X-α-Gal (–AHLT/X) medium. Positive control: pGADT7-T/pGBKT7–53; negative control: pGADT7-T/pGBKT7-Lam. (**F**) MdERF3 was overexpressed in OE-*MdNAC5^A^* and OE-*MdNAC5^T^* apple calli. (**G, H**) The protein level and RNA level of OE-*MdNAC5^A^* + OE-*MdERF3* and OE-*MdNAC5^T^* + OE-*MdERF3* were identified. (I) The expression levels of *MdACS1* in transgenic calli. Values are means of three replicates ± SD (Turkey’s test, ^***^  *P* < 0.001, ^**^  *P* < 0.01, ^*^  *P* < 0.05).

First, modeling of the amino acids of MdNAC5^A^ and MdNAC5^T^ revealed changes in the structure of the two proteins (Fig. 7A). Docking prediction of MdNAC5^A^ and MdNAC5^T^ on MdERF3 was carried out, and the optimal binding site of these proteins differed (Fig. 7B). Next, we used *in vivo* and *in vitro* assays to verify whether MdNAC5^A^ and MdNAC5^T^ interacted with MdERF3. MdNAC5^A^ and MdNAC5^T^ with MdERF3 were fused into the complementary fragments of the nLuc/cLuc reporter vector and then transformed into tobacco leaves. Further luciferase complementation imaging assays revealed that MdNAC5^A^ and MdNAC5^T^ interact with MdERF3 (Fig. 7C). BiFC assays indicated that MdNAC5^A^ and MdNAC5^T^ interact with MdERF3 in the nucleus of *Arabidopsis* protoplasts according to fluorescence data (Fig. 7D). Furthermore, the coding regions of MdNAC5^A^ and MdNAC5^T^ were cloned into the pGBKT7 vector and transformed into Y2H yeast cells; no self-activating activity of MdNAC5^A^_△175–364_/MdNAC5^T^_△175–364_ was detected on SD/−Trp medium (Fig. S12). The pGADT7-MdERF3 and MdNAC5^A^_△175–364_/MdNAC5^T^_△175–364_ constructs were fused to the pGBKT7 vector and cotransformed into yeast cells; the growth of the transformants was detected on SD/−Leu-Thr-Ade–His medium. Yeast cells containing pGBKT7-MdNAC5^A^_△175–364_-pGADT7-MdERF3 were able to grow more densely on SD/−Leu-Thr-Ade–His medium than on MdNAC5^T^_△175–364_-pGADT7-MdERF3, which indicates that MdNAC5^A^_△175–364_ might interact more strongly with MdERF3 *in vitro* than MdNAC5^T^_△175–364_ (Fig. 7E). In addition, *MdERF3* was overexpressed in OE-*MdNAC5^A^* and OE-*MdNAC5*^T^ apple calli, and the protein and RNA levels of OE-*MdNAC5^A^* + OE-*MdERF3* and OE-*MdNAC5^T^* + OE-*MdERF3* were identified (Fig. 7F–H). RT-qPCR analysis showed that the expression of *MdACS1* was significantly higher in OE-*MdNAC5^A^* + OE-*MdERF3* apple calli than in OE-*MdNAC5T* + OE-*MdERF3* apple calli (Fig. 7I). In sum, these findings indicate that the interactions of MdNAC5^A^ and MdNAC5^T^ with *MdERF3* in the nucleus regulate the expression of *MdACS1* in differentiation.

## Discussion

The ripening of apple fruit involves complex changes in texture, which is a key factor affecting fruit quality [2]. Excessive firmness and over-softening are undesirable, and this has an effect on the acceptance of fruit by consumers and reduces the shelf life of fruit [3, 4]. Fruit firmness is controlled by multiple genes, and this increases the difficulty of selecting high-quality varieties [7, 65]. The cross-breeding of apple plants is an important approach for improving fruit quality, but traditional cross-breeding approaches are lacking in efficiency. Therefore, the use of high-quality genetic bin maps for the QTL mapping of quality traits is critically important for analyzing the genetic and molecular mechanisms underlying fruit quality variation. Many studies have used SNPs to construct genetic maps of apple [16, 18, 19]. The combined use of SSR and SNP markers for constructing genetic maps can significantly increase their accuracy [20–22]. Genetic maps have been constructed for an increasing number of species based on SNP information, and bins have been used as the smallest recombination unit [66, 67]. We obtained 5014 high-quality bin markers via re-sequencing, and a high-density genetic map of apple with an added length of 2213.23 cM and an average genetic distance of 0.44 cM was obtained (Table 1). The quality of the genetic maps generated in this study was higher than those in previous studies due to improvements in sequencing technology and marker density; this genetic map could thus be used for the fine-mapping of QTLs.

The reliability of the phenotypic data collected via sensory evaluations can be poor, and this can lead to the development of inaccurate markers for constructing genetic maps, which decreases the accuracy of the mapping results [3, 6]. Therefore, many of the previous mapping results were not sufficient for identifying specific genes. The QTLs related to firmness, such as those on chromosomes 1, 3, 4, 5, 10, 14, 15, and 16, have been identified in multiple populations [34, 41, 68–71]. Most fruit ripening-related QTLs were mainly concentrated on chromosomes 3, 9, 10, 15, and 16 [34, 35, 40]. Although these five trait mapping intervals were concentrated on chr3, the LOD values and phenotypic variance explained for each trait on chromosomes varied (Fig. S7). For the markers in the region with the largest LOD on chr3, the variance explained for HD was 37.3%, 42.8%, and 39.1% in 2018, 2019, and 2020, respectively. The maximum variance explained for flesh firmness was 15.5–26.3%. This locus on chr3 explained most of the variation in HD. Variation in flesh firmness was also regulated by other loci [38, 41, 45, 49, 51, 72]. Most of the QTL intervals overlapped or were similar to those reported in previous studies, indicating that these genomic regions play an important role in controlling apple firmness or ripening traits. These findings confirmed that the QTLs related to fruit firmness and ripening identified in our study were reliable.

Migicovsky *et al*. [35] showed that the SNP of *NAC18.1* on Chr3:30698039 is polymorphic. Fruit ripening earlier and are less firm when the allele is homozygous A/A; however, fruit mature later and are firm when the allele is homozygous C/C. However, in this study, we showed that *MdNAC5* is a candidate gene for the major-effect fruit firmness and ripening QTLs (Figs 3 and S6). Firstly, we describe the detailed information of all QTL loci related to fruit hardness and maturity in Table S7. We found that the contribution rate of Marker in these QTL loci to phenotype is different, and the Marker containing *MdNAC5* gene has the highest contribution rate and the LOD value corresponding to this marker is also the highest. Furthermore, we also located the gene by BSA analysis and reduced the location interval to 0.65 Mb, with 48 candidate genes in this interval. ‘Fuji’ and ‘Cripp's Pink’, as parents of this research population, have differences in fruit firmness at the development stage [64], and the results of transcriptome show that the expression of *MdNAC5* increases with the decrease of fruit firmness. In addition, the expression of *MdNAC5* was the most significant among the five DEGs in the minimum interval. A nonsynonymous A-to-T SNP in *MdNAC5* can distinguish fruit firmness and ripening period in hybrids. Differences in tomato ripening were observed following the overexpression of *MdNAC5^A^* and *MdNAC5^T^*, and ripening occurred earlier when *MdNAC5^A^* was overexpressed compared with when *MdNAC5^T^* was overexpressed. The TF SlNAC-NOR was identified as the main regulator of climacteric ripening in the tomato natural mutant NOR [53]. Mutation of nor completely inhibited fruit ripening, but the immature tomato fruit phenotype was improved to a greater degree when *MdNAC5^A^* was overexpressed compared with when *MdNAC5^T^* was overexpressed.

Studies have shown that ethylene can promote the ripening and firmness decrease of the climacteric fruits such as apples, while ethylene production is less in nonclimacteric fruits [38, 46, 69]. Wang *et al*. [60] showed that CmNAC-NOR^S,N^ and CmNAC-Nor^A,S^ are two natural mutants of *CmNAC-NOR*. Climacteric haplotype CmNAC-NOR^S,N^ could significantly induce the expression of downstream maturation related genes, while nonclimacteric haploid CmNAC-Nor^A,S^ could not. Here, a nonsynonymous SNP in *MdNAC5* causes the coding amino acid to change from methionine to leucine. Thus, we speculate that MdNAC5 binds to *MdERF3/MdACS1* in the ethylene signal pathway to promote ethylene synthesis, which affects fruit firmness and ripening. Biochemical analyses revealed that MdNAC5^A^ and MdNAC5^T^ bind directly to the *MdERF3/MdACS1* promoter to activate its transcription (Fig. 6). We also found that the content of Met and ACC in *MdNAC5^A^* transgenic materials (Fig. 7, S13) was higher than that in *MdNAC5^T^*, which explained differences in fruit firmness and ripening between *MdNAC5^A^* and *MdNAC5^T^*. Previous studies have reported that MdERF3 can activate the transcription of *MdACS1* [46, 69]. In this study, we found that the two genotypes of the MdNAC5 mutation differentially regulate the transcriptional activation of *MdACS1*. The structural analysis of MdNAC5^A^ and MdNAC5^T^ proteins is helpful to reveal how the A-to-T mutation affects their transcriptional activation activity. However, further study on the SNP variation of the two *MdNAC5* genotypes will help to determine its role in fruit ripening of the climacteric type. The experiments shown in Fig. 7F–H demonstrate that the interaction between the two MdNAC5 genotypes and MdERF3 enhances *MdACS1* expression. These findings indicate that the combined interaction of MdNAC5 and MdERF3 has a significantly greater transcriptional effect on *MdACS1* than either factor alone. In addition, the transformation efficiency of stable genetic transformation method in apple is still low. Although two genotypes of apple transgenic have been obtained because the fruit acquisition cycle is long, there is still a long way to go in the future. Therefore, after obtaining the transgenic fruits of MdNAC5^A^ and MdNAC5^T^ genotypes, we will continue to clarify how they fine regulate fruit ripening and firmness.

**Figure 8:**
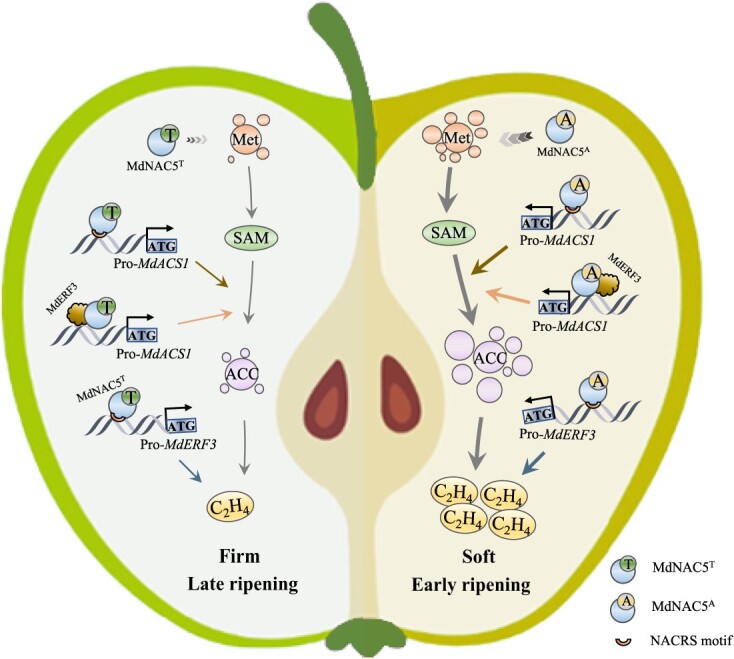
A model showing the roles of *MdNAC5* alleles associated with fruit firmness and ripening variation in apple. In apple, mutation of the A/T allele in *MdNAC5* leads to differences in fruit firmness and ripening. MdNAC5^A^ and MdNAC5^T^ promotes its activity by directly binding to DNA in the promoter region of *MdACS1* and *MdERF3*. MdNAC5^A^ results in the production of more Met and ACC and releases more ethylene than MdNAC5^T^, which reduces fruit firmness and accelerates fruit ripening.

In sum, we established a model that clarifies the parts of *MdNAC5* alleles in directing variety with the fruit firmness and ripening of apple (Fig. 8). In apple, the mutation of the A-T allele in *MdNAC5* leads to contrasts in fruit firmness and ripening. The interaction between MdNAC5^A^ and MdERF3 advances the expression of MdACS1 to a more noteworthy degree than the interaction between MdNAC5^T^ and MdERF3. MdNAC5^A^ and MdNAC5^T^ advance the activity of MdACS1 to diverse degrees by directly binding to the promoter region of *MdACS1* and *MdERF3*. *MdNAC5^A^* produces more Met and ACC than *MdNAC5^T^*, which reduces fruit firmness and accelerates fruit ripening.

## Materials and methods

### Plant materials and growth conditions

The plant materials used in this experiment were crossed with ‘Fuji’ as the female parent and ‘Cripp's Pink’ as the male parent in 2009. The hybrid progenies of ‘Fuji’ × ‘Cripp's Pink’ were grafted onto M_26_ rootstock (*M. domestica* Borkh.) in August 2012. The hybrids were planted in an orchard at a density of 0.5 × 4 m, with a total of 935 plants. We randomly selected 294 individuals to construct a genetic map for QTL mapping analysis. For consistency, the parents ‘Fuji’ and ‘Cripp's Pink’ were planted in the same orchard as their progeny. All plants were planted at the Experimental Station of Northwest A&F University, which is located in Baishui County, Shaanxi Province, China 35°21 N, 109°55′ E; 850 m. RNA was extracted from roots, stems, leaves, fully bloomed flowers, and fruits of uniform size and appearance and with no external damage. The maturity of the apple fruit was assessed using internal and external indicators, including the touch firmness of the fruit, the ease with which the stalk can be shed, the color of the seed coat, the background color of the fruit peel, and the color of the flesh; starch staining was also performed to ensure that the selected fruits were at the same stage of physiological maturity.

Apple calli (‘Orin’) was used for the stable transformation [73]. For each experimental replicate, eight ‘Fuji’ apples with similar growth 165 DAFB were collected for analysis of transient injection, gene expression, and flesh firmness. The Ailsa Craig (AC) tomato (*Solanum lycopersicum*) cultivar and tobacco plantlets were planted in organic fertilizer at 25°C under a 16 h/8 h light/dark cycle. The content of Met and ACC was measured using a liquid chromatography-mass spectrometry system (LC–MS) following a previously described method [74].

### DNA extraction and re-sequencing

Genomic DNA was extracted from the young leaves of 300 F_1_ individuals and two parents (‘Fuji’ and ‘Cripp's Pink’) using the CTAB method. Ultrasonic fragmentation was used to randomly interrupt the DNA after the samples were qualified. The A base was added at the 3′ end, the sequence was then spliced, purified, and amplified by PCR to construct the sequencing library using Illumina’s NEB Next Ultra DNA Library Prep Kit (NEB, USA). After quantification using a Bioanalyzer (Agilent, USA), libraries were paired-end sequenced by an Illumina HiSeq 2500 system.

### SNP identification

The original image data files were transformed into sequenced reads by base-calling using Illumina Casava 2.17. Reads in which Q ≤ 10 accounted for more than 50% of the bases of an entire reads were retained. The clean reads were mapped to the genome of apple GDDH13 v1.1 using BWA v 0.7.5a (with the parameter -n 1) [75]. The sample sequencing depth, genome coverage, and detected mutations were analyzed. Picard software was used to remove duplicates and analyze the local weight ratio and other pretreatments to ensure the accuracy of the detected SNPs. Picard and GATK software were used to remove duplicates and analyze the local weight ratio and other pretreatments to ensure the accuracy of the detected SNPs [76]. GATK was then used for SNP detection and filtering and to obtain the final set of SNPs loci.

### Genetic map construction and collinearity analysis

To obtain chromosomes, SNP markers were first filtered and screened. After detecting the genetic linkage of paired markers, the recombination rate between markers was used to determine the linkage phase. Genotypes lacking linkage were used to fill in the gaps, and genotypic errors were corrected. The bins were divided based on recombination frequency information; they were then divided into chromosomes. HighMap software [77] was used to determine the linear arrangement of markers on chromosomes, estimate the genetic distance between adjacent markers on chromosomes, and generate the high-density genetic map. Finally, linkage relationships were determined, and collinearity analysis was performed using the genetic map.

### Trait phenotyping and heritability calculation

During three consecutive fruit ripening seasons (from 2018 to 2020), 6–10 mature fruits of the same size were randomly collected around the crown for trait measurements. Measurements of FF were taken from the fruit of each plant in the hybrid population, and the HD of fruit was recorded (expressed as DAFB). FF was measured using a TA.XT-21 texture analyzer (Stable Micro Systems Ltd., Godalming, Surrey, UK) [38]. The sensory evaluation of flesh texture was performed following the method of Amyotte *et al*. [3] with modifications, and fruits with a normal texture were used as a control. Flesh texture was divided into five types: pliable-soft, mealy-soft, soft-crisp, firm-crisp, and firm. Flesh thickness was divided into three grades, high, middle, and lower, and flesh firmness was divided into five grades from low to high using an ordinal scale from 1 to 5. The fruit evaluation team comprised various experts and students, and the final results were summarized using the evaluation results of all members in 2019 and 2020.

### QTLs mapping analysis

The R/qtl package was utilized to calculate the genetic distance, identify recombinant breakpoints, and generate bins and QTLs [78]. QTL analysis of the fruit traits was performed using MapQTL® 6 software. The QTL effect was determined by using the threshold of the statistical significance of 1000 permutations (*P* < 0.05).

### BSA-seq and QTL identification

According to the 2018–2020 apple firmness phenotypes, 50 extremely firm or soft offspring were randomly selected from ‘Fuji’ × ‘Cripp's Pink’ individuals. The leaf DNA was extracted and mixed in equal volumes to form two mixing pools (excluding the genetic map samples). The methods for resequencing the two mixed pools, analyzing the sequencing data, and comparing the data against the reference genome were the same as those described in the genetic map analyses. The genotype frequency of alleles between the two mixed pools was calculated to identify regions associated with firmness. SNPs and InDels in these areas were also annotated. After obtaining high-quality SNPs, ED and Δ(SNP-index) values were used to identify regions correlated with firmness [26].

### RNA sequencing, transcriptomic data analysis, and candidate genes prediction

Total RNA of apple calli (WT, OE-*MdNAC5^A^* and OE-*MdNAC5^T^*) was extracted using an RNAprep Pure Plant Kit (TIANGEN, Beijing, China). RNA-seq and preliminary analysis of mRNA were conducted by Personalbio (Shanghai, China). Finally, DEGs were recognized utilizing DESeq2 utilizing the taking after criteria: |fold change| ≥2 and false discovery rate < 0.01. All distinguished DEGs were subjected to GO and KEGG pathway enrichment analyses; the threshold for statistical significance in these analyses was *P* ≤ 0.01 [64].

Colocalized QTL intervals of the genetic map and BSA-Seq were mapped using the GDDH13 v1.1 apple genome. Genes in the same region were obtained from the Rosaceae genome database website (https://www.rosaceae.org/species/malus/malus_x_domestica/genome_GDDH13_v1.1). The functions of genes were annotated via Gene Ontology (GO) and Kyoto Encyclopedia of Genes and Genomes (KEGG) pathway enrichment analyses. The results of these analyses, along with the transcriptome data obtained from the developing fruits of their parents (‘Fuji’ and ‘Cripp's Pink’), were used to identify the possible candidate genes. Genes that were highly expressed during fruit development were identified as candidate genes [64].

### Gene expression analysis

Total plant RNA was extracted from fresh roots, stems, leaves, flowers, and developing fruits. cDNA was synthesized from 1000 ng of RNA utilizing a PrimeScript RT Master Mix Kit (Takara, Dalian, China). Real-time quantitative polymerase chain reaction (RT-qPCR) was conducted using a StepOnePlus analyzer (Thermo Fisher Science Inc.). RT-qPCR reactions (20 μL) were conducted using 2× Accurate Taq (Accurate Biology, China). *MdCTAB* were used as the internal reference genes to normalize the expression levels of all genes. The gene expression levels were calculated using the 2^–ΔΔCt^ method [79].

### Stable genetic transformation of tomato and apple calli, and the transformation of apple fruit

The coding sequences (CDSs) of *MdNAC5^A^*, *MdNAC5^T^*, and *MdRERF3* were cloned to the pCAMBIA2300 vector (pC2300) with the 35S promoter. Wild-type tomato and apple calli were transformed using the *Agrobacterium tumefaciens*-mediated method to obtain transgenic plants [73]. Specific partial sequences of 300–400 bp from *MdNAC5* were cloned to the pTRV2 vector. The ‘Fuji’ apple fruits at 165 DAFB were used for transient expression following methods described in previous studies [73]. Briefly, after preparing the bacterial suspension, Fuji apples at 165 DAFB were injected with five 1 ml doses of the solution. Seven days later, the flesh firmness at the injection sites was measured. Total protein extraction and western blotting (WB) analysis of transgenic apple calli were performed following the methods described in previous studies [73]. The green fluorescent protein antibody used in this experiment was obtained from Abmart Medical Technology (Shanghai, China) Co., Ltd.

### Transcription factor–DNA interaction validation

For yeast one-hybrid (Y1H) assays, 300-bp fragments containing NAC elements (NACRS, CATGTG) in the promoters of *MdERF3* and *MdACS1* were truncated and inserted into the pAbAi vector, and the full-length CDSs of *MdNAC5^A^* and *MdNAC5^T^* were cloned and inserted into the pGADT7 vector. Based on previous studies, the minimum AbA inhibition concentrations of pAbAi-*MdERF3p1*, *pAbAi-MdERF3p2*, and pAbAi-*MdACS1* were determined on SD/–Ura medium; they were co-transformed with pGADT7-MdNAC5^A^ and pGADT7-MdNAC5^T^ into yeast cells and then cultured on SD/−Leu medium [48].

For dual-luciferase (Dual-LUC) assays, promoter sequences 2000 bp upstream of *MdERF3* and *MdACS1* were cloned to pGreenII 0800-LUC (the reporter vector, LUC), and the CDSs of *MdNAC5^A^* and *MdNAC5^T^* were cloned to pGreenII 62SK (the effector vector, 62SK). After the vectors LUC-*MdERF3*, LUC-*MdACS1*, 62SK-MdNAC5^A^, and 62SK-MdNAC5^T^ were transformed into GV3101, the *A. tumefaciens* effector and reporter vectors were coinfiltrated at a ratio of 1:9 in tobacco leaves and cultured for 3 days. The chemiluminescence values of firefly luciferase (LUC) and *Renilla* luciferase (REN) were measured using a microplate reader (Infinite M200pro, Tecan), and the LUC/REN value was obtained. Images were taken using a plant living molecular marker imaging system (CCD) (Lumazone Pylon 2048B).

For EMSAs, the CDSs of *MdNAC5^A^* and *MdNAC5^T^* were inserted into the pET-32a vector and expressed in the *Escherichia coli* strain BL21 (DE3) with 0.8 mM isopropyl-β-d-thiogalactoside for 14 h at 21°C. The histidine-tagged protein was purified using a prepacked gravity column (Ni-TED 6FF) from Sangon Biotech (Shanghai, China). According to the sequences of the *MdACS1* and *MdERF3* promoters, specific hot probes, cold probes, and mutation probes were designed, and the probes were labeled in Sangon Biotech (Shanghai, China). EMSAs were performed using the instructions in the EMSA/Gel-Shift Kit (Beyotirne, Shanghai, China).

For CHIP-qPCR assays, we followed the method described by Wei et al. [63] Briefly, apple calli was crosslinked with 1% (w/v) formaldehyde to stabilize protein–DNA interactions. Chromatin was then extracted and fragmented to approximately 200–800 bp using sonication. Immunoprecipitation was performed overnight at 4°C with or without an anti-GFP (AE012, ABclonal, Wuhan, China) antibody. Chromatin–antibody complexes were precipitated using ChIP-grade protein A/G agarose beads (Thermo Scientific, Waltham, MA, USA) and subsequently washed four times with various washing buffers. Following reverse crosslinking and protein removal, the recovered DNA was subjected to qPCR analysis. The primers used for ChIP-qPCR are listed in Table S13, with actin serving as the reference gene.

### Protein–protein interaction validation

Amino acid sequences for MdNAC5^A^, MdNAC5^T^, and MdERF3 were obtained from the genomic database of apple GDDH13 v1.1. The protein structures of MdNAC5^A^, MdNAC5^T^, and MdERF3 were modeled using the alphafold program [80]. The spatial docking of MdNAC5^A^-MdERF3 and MdNAC5^T^-MdERF3 proteins was predicted and scored by the GRAMM DOCKING and Rosetta programs [81].

For Y2H assays, the CDSs of MdERF3 were inserted into the pGADT7 vector, and MdNAC5^A^_∆175–364_ and MdNAC5^T^_∆175–364_ (transcriptional-free self-activation fragment) were cloned and inserted into the pGBKT7 bait vector. The pGADT7-MdERF3 vector was co-transformed into Y2H yeast cells with pGBKT7-MdNAC5^A^_∆175–364_ and pGBKT7-MdNAC5^T^_∆175–364_ and cultured on SD/−Leu medium. The specific steps were performed following the instructions in the Matchmaker™ Yeast Two-Hybrid Library Screening System kit (Clontech, Beijing, China).

For BiFC assays, the CDS of *MdERF3* was inserted into the pSPYNE vector (the YFP contained N-terminal), and MdNAC5^A^ and MdNAC5^T^ were cloned to the pSPYCE vector (the YFP contained C-terminal). Next, the fluorescence signal of YFP was detected by confocal laser-scanning microscopy (FV3000, Olympus) after co-expression in *Arabidopsis thaliana* protoplasts.

For Firefly LCI assays, the CDS of *MdERF3* was cloned to the pCAMBIA1300-35S-cLUC vector, and MdNAC5^A^ and MdNAC5^T^ were cloned to the pCAMBIA1300-35S-nLUC vector. Then, the expression vector *A. tumefaciens* was coinfiltrated into tobacco leaves and photographed after 3 days of culture.

### Statistical analysis

The R software (4.3.1) was used for graphic drawing and correlation analysis. All primers used are listed in Table S13.

## Supplementary Material

Web_Material_uhae284

## Data Availability

Sequencing raw reads are deposited in the NCBI under the following accession numbers: resequencing data of 294 hybrids and their parents (PRJNA816648), the data of BSA-seq (PRJNA1143202), and transcriptome data of fruit calli (PRJNA1141185). The transcriptome data of the parents ‘Fuji’ and ‘Cripp's Pink’ during the development stage were obtained from Liu *et al*. [64] (PRJNA728501).
