# Supplementary material for: Natural variation in MdNAC5 contributes to fruit firmness and ripening divergence in apple
Source: Hortic Res. 2024 Oct 8;12(1):uhae284. doi: 10.1093/hr/uhae284 (PMC11758708; doi:10.1093/hr/uhae284)
Supplement: Web_Material_uhae284 [file web_material_uhae284.zip › R2-supplementary figures.docx]

# Supplementary figures

**
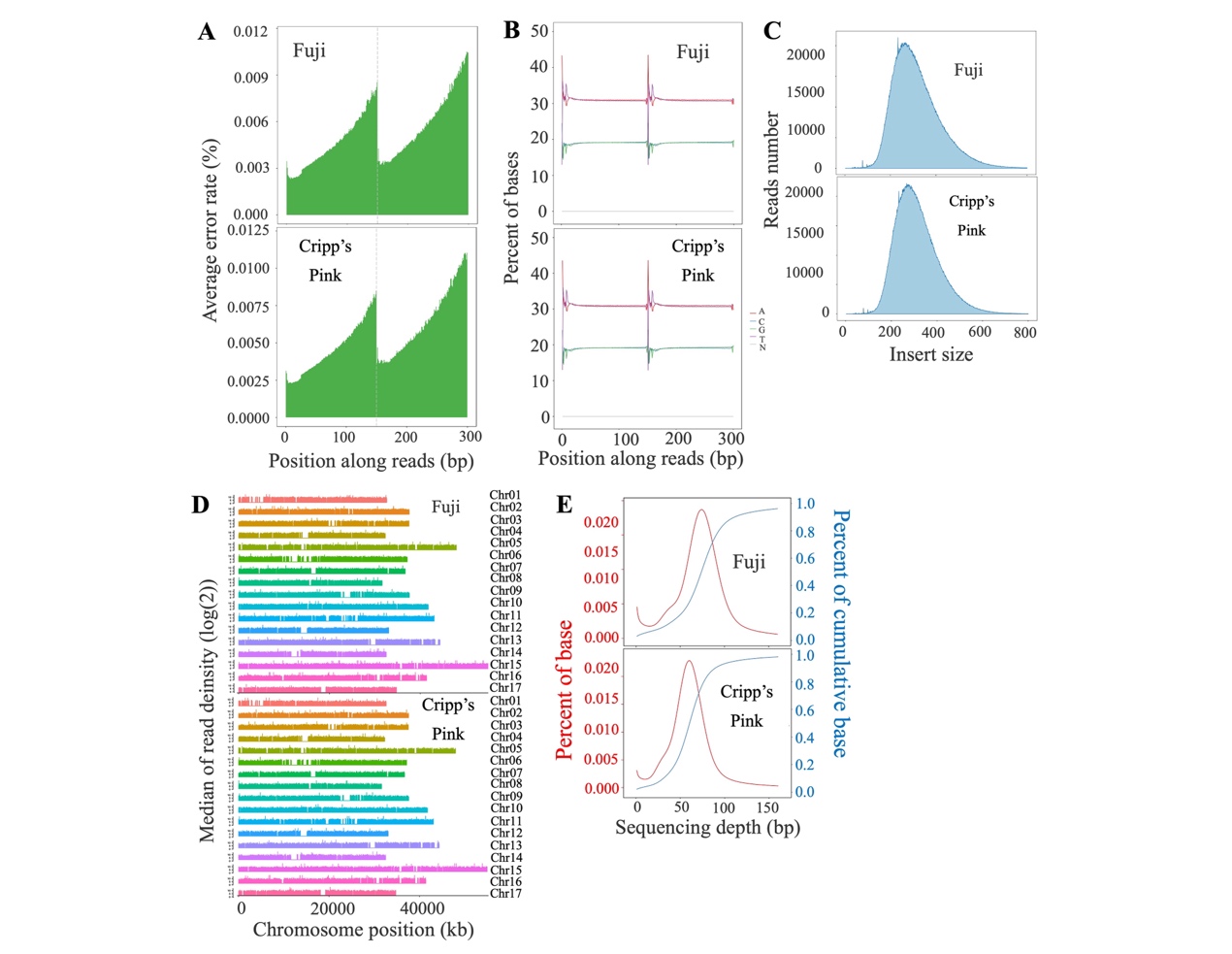
**

**Figure S1 Quality assessment of re-sequencing of parent ‘Fuji’ and ‘Cripp’s Pink’ apples.** Base mass **(A)**, base ratio **(B)**, insert size **(C)** and coverage depth **(D)** distributions for re-sequencing of parent ‘Fuji’ and ‘Cripp’s Pink’ apples. **(E)** The sequencing depth distributions of ‘Fuji’ and ‘Cripp’s Pink’ apples.


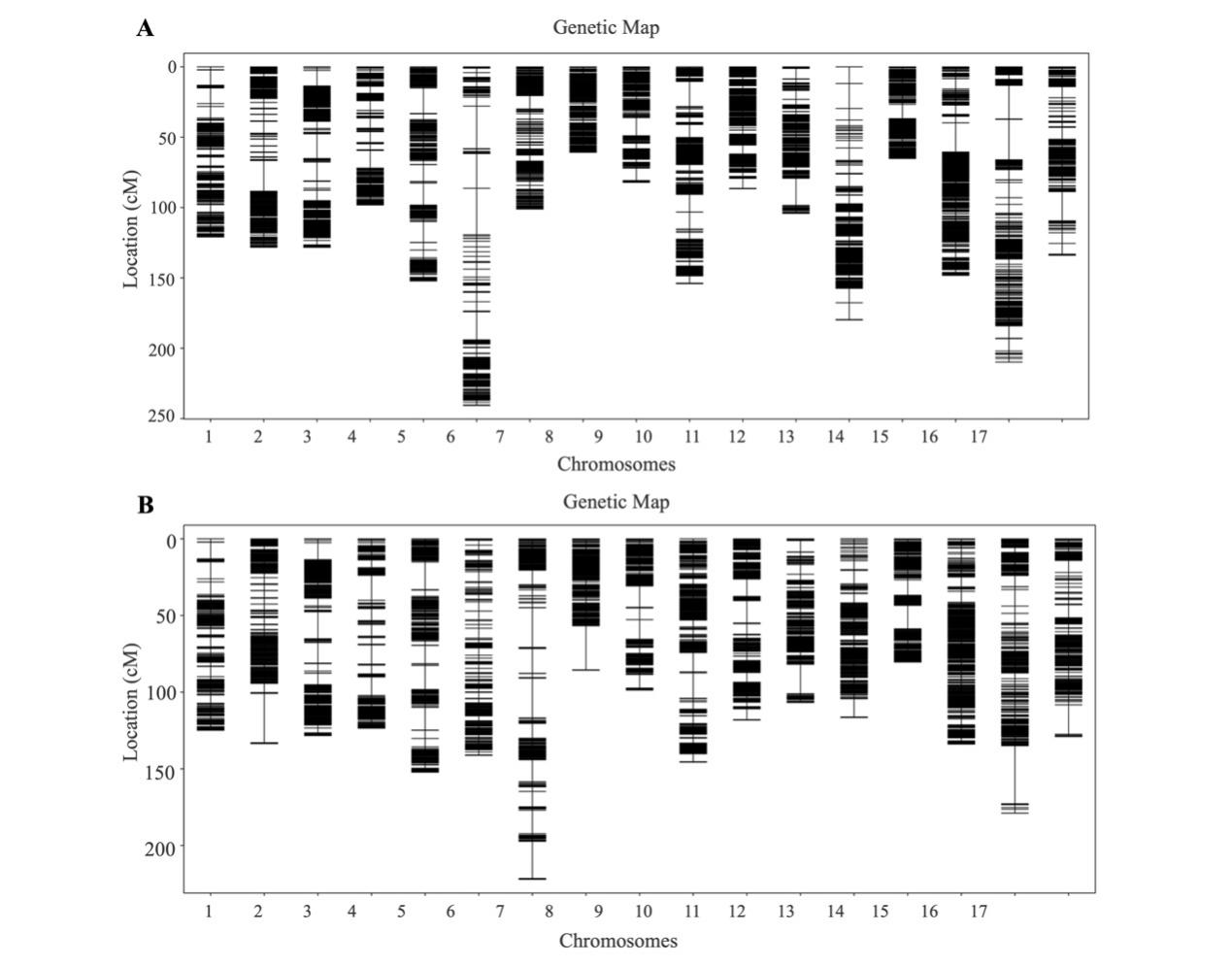


**Figure S2 The distribution of bin markers on the 17 chromosomes of female (A) and male (B).**

**
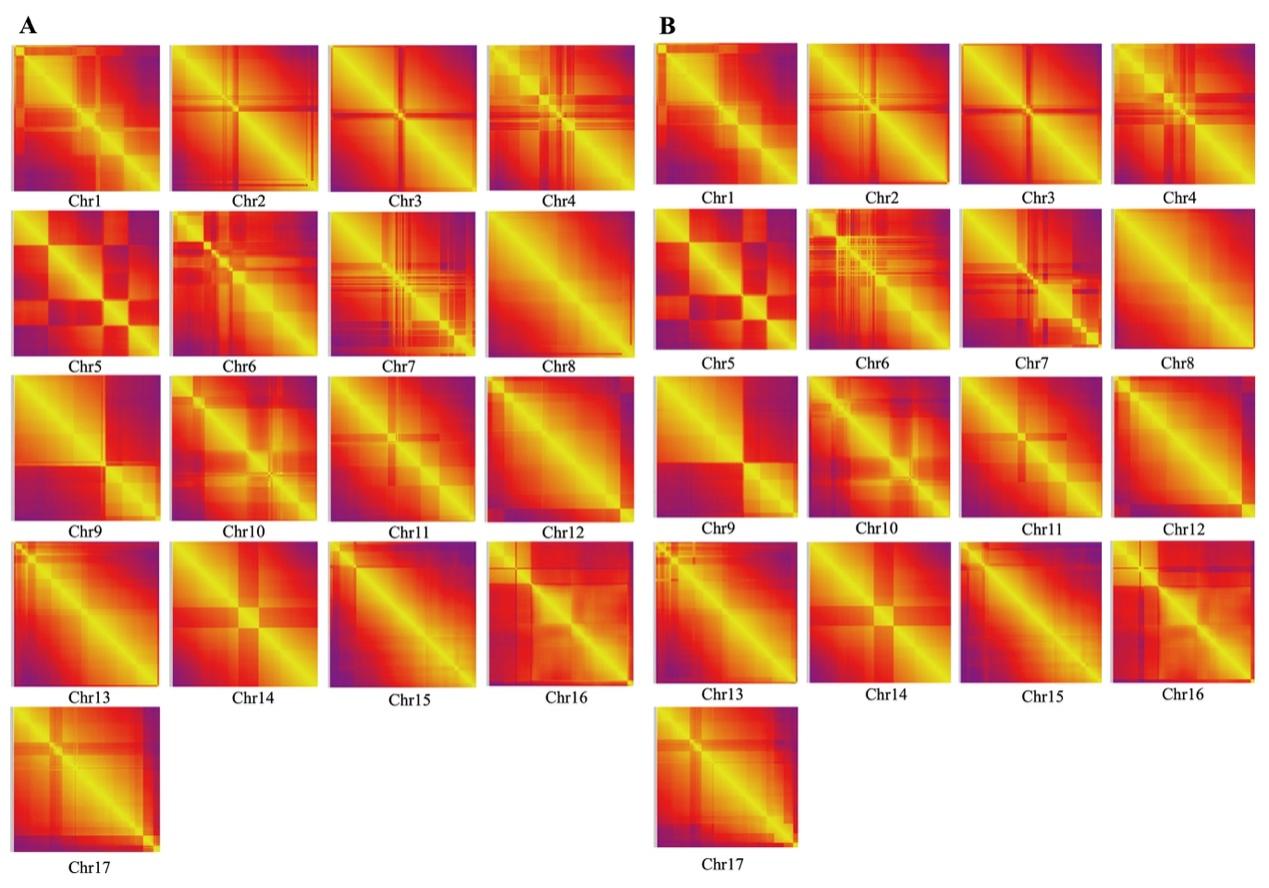
**

**Figure S3 The heat maps show distances and recombination rates between markers of female (A) and male (B) on the map.** The yellow to purple indicates the recombination rate. Gray indicates a lack of recombination rate between the markers that only segregated in different parents.

**
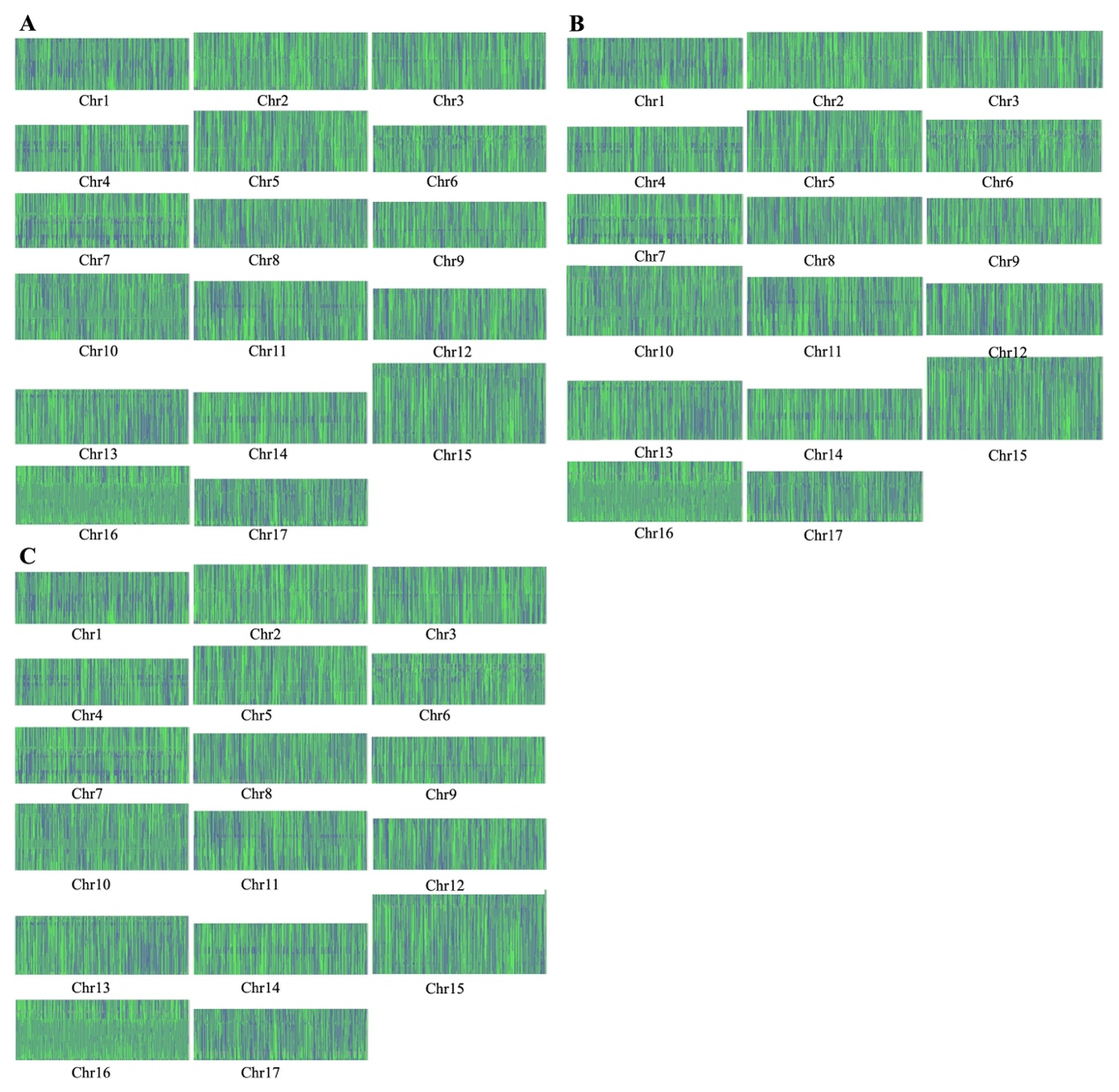
**

**Figure S4 Haplotype map of different chromosomes of neutral (A), female (B) and male (C).** Rows represent markers, arranged in order of their positions on the chromosome; columns represent chromosomes. There are color changes on the same column where recombination events occurred.

**
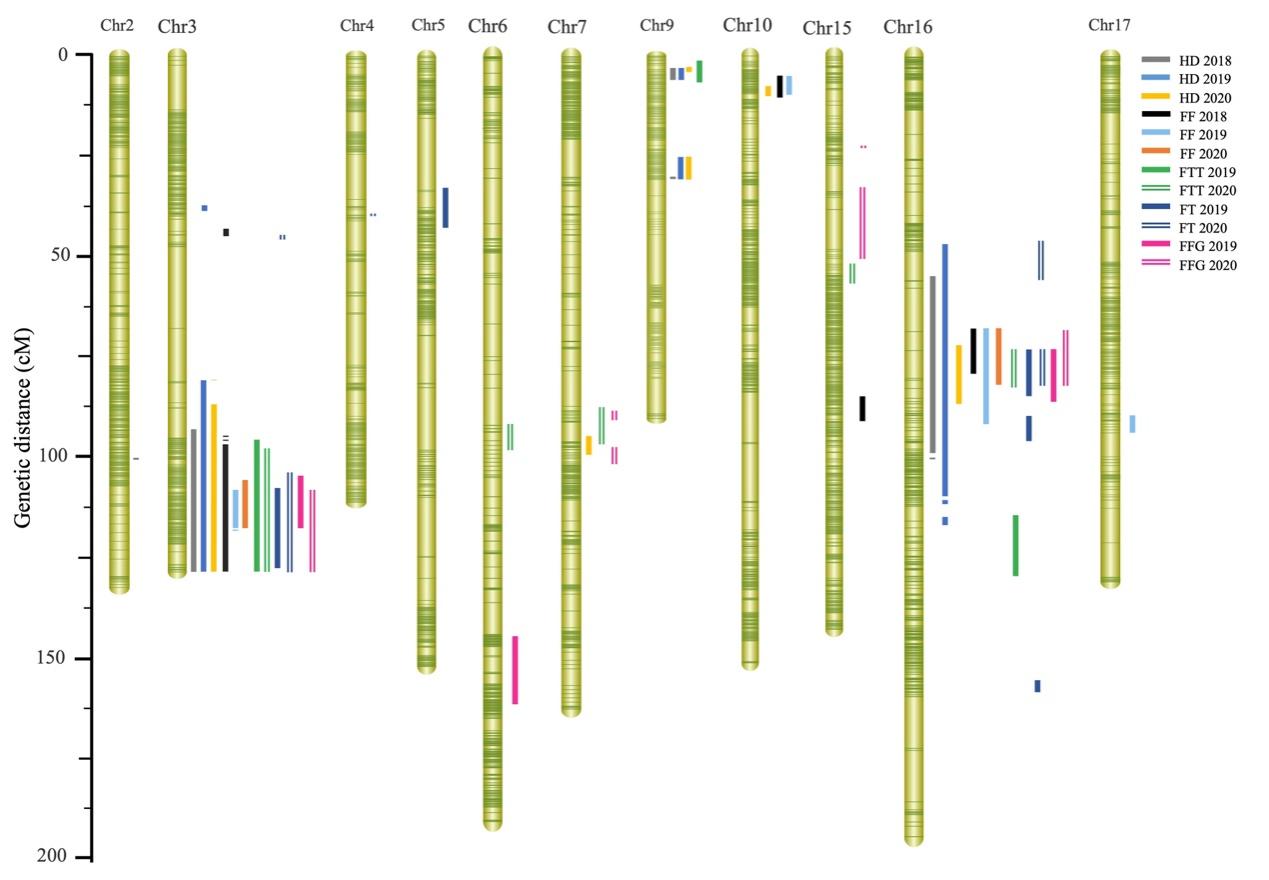
**

**Figure S5 QTL distributions of 5 fruit-related traits on high-density genetic map.** Colored bars represent the potential QTLs for different fruit traits (harvest date: HD; flesh firmness: FF; flesh texture type: FTT; flesh thickness: FT; flesh firmness grade: FFG). The left coordinates represent the physical distance of the chromosomes. The details of QTLs are list in **Table S7**.

**
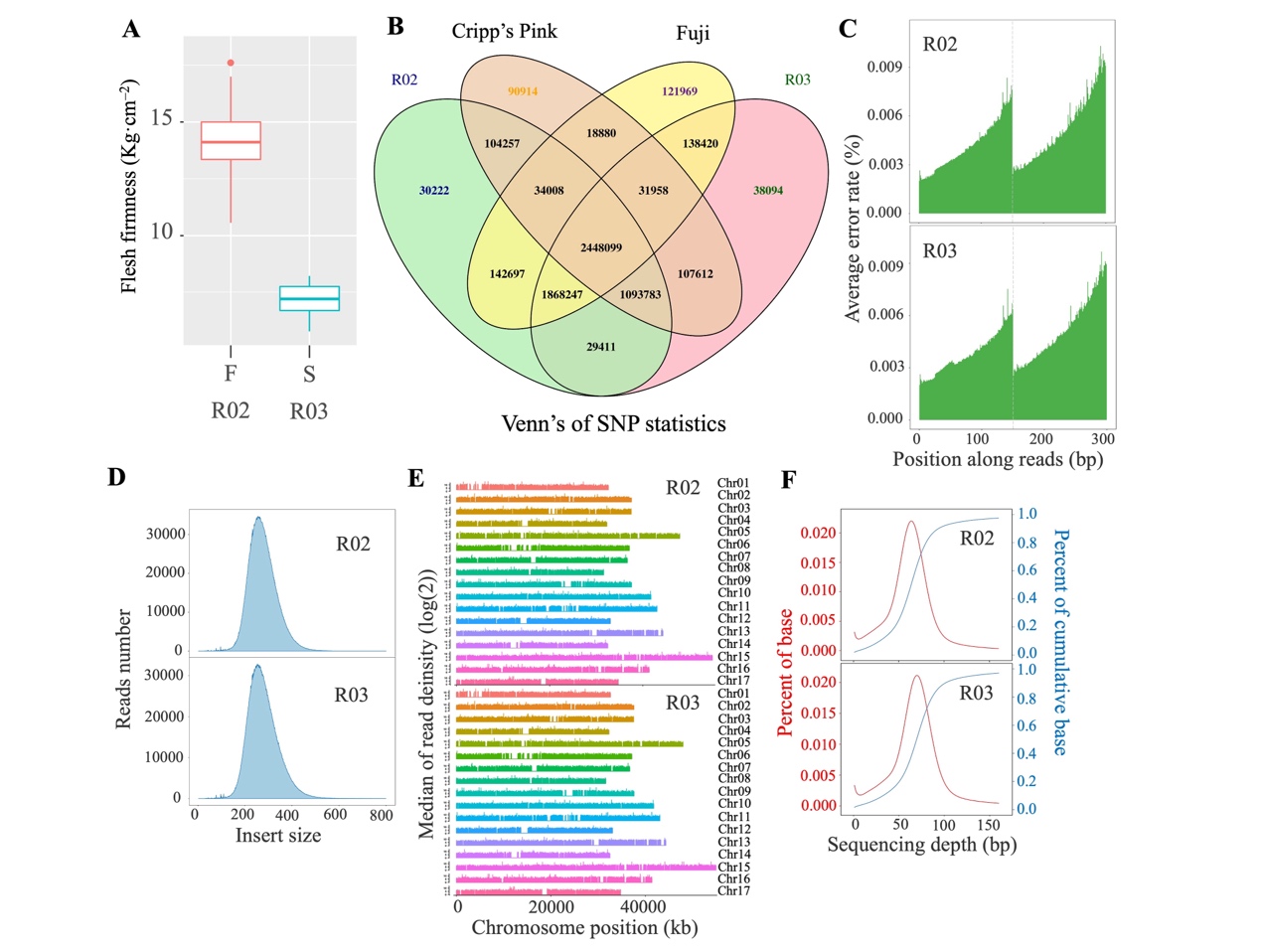
**

**Figure S6 Re-sequencing quality assessment for fruit firmness (firm-R02/soft-R03) in a mixed pool of F_1_ generations of ‘Fuji’ and ‘Cripp’s Pink’ (eliminating the plants that construct the genetic map).** **(A)** Distributions of extreme firmness phenotypes (three-year average from 2018 to 2020) of R02 and R03. **(B)** Venn’s of SNP differences between ‘Fuji’, ‘Cripp’s Pink’, R02 and R03. Base mass **(C)**, insert size **(D)**, coverage depth **(E)**, sequencing depth **(F)** distributions for re-sequencing of R02 and R03.

**
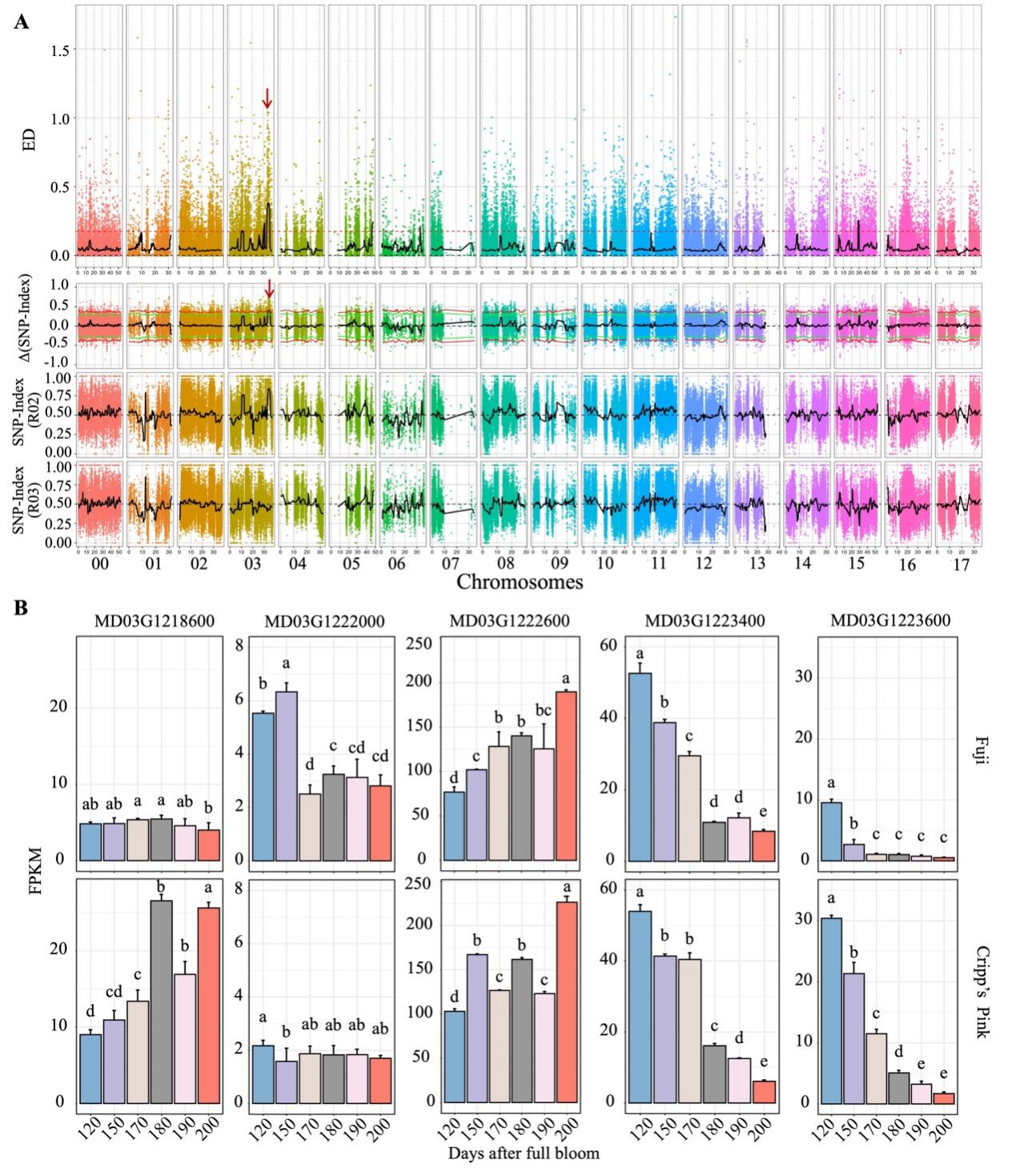
**

**Figure S7 Identification and expression analyses of genes identified through QTL analysis of flesh firmness. (A)** QTLs analysis of apple fruit firmness based on BSA-seq. The red arrow indicates the location of the MD03G1222600. **(B)** The FPKM of genes in the common interval during the development of parents ‘Fuji’ and ‘Cripp’s Pink’. The bars are mean the values of three replicates (± SD), and diverse letters show differ significantly (Turkey's test, *P* < 0.05).

**
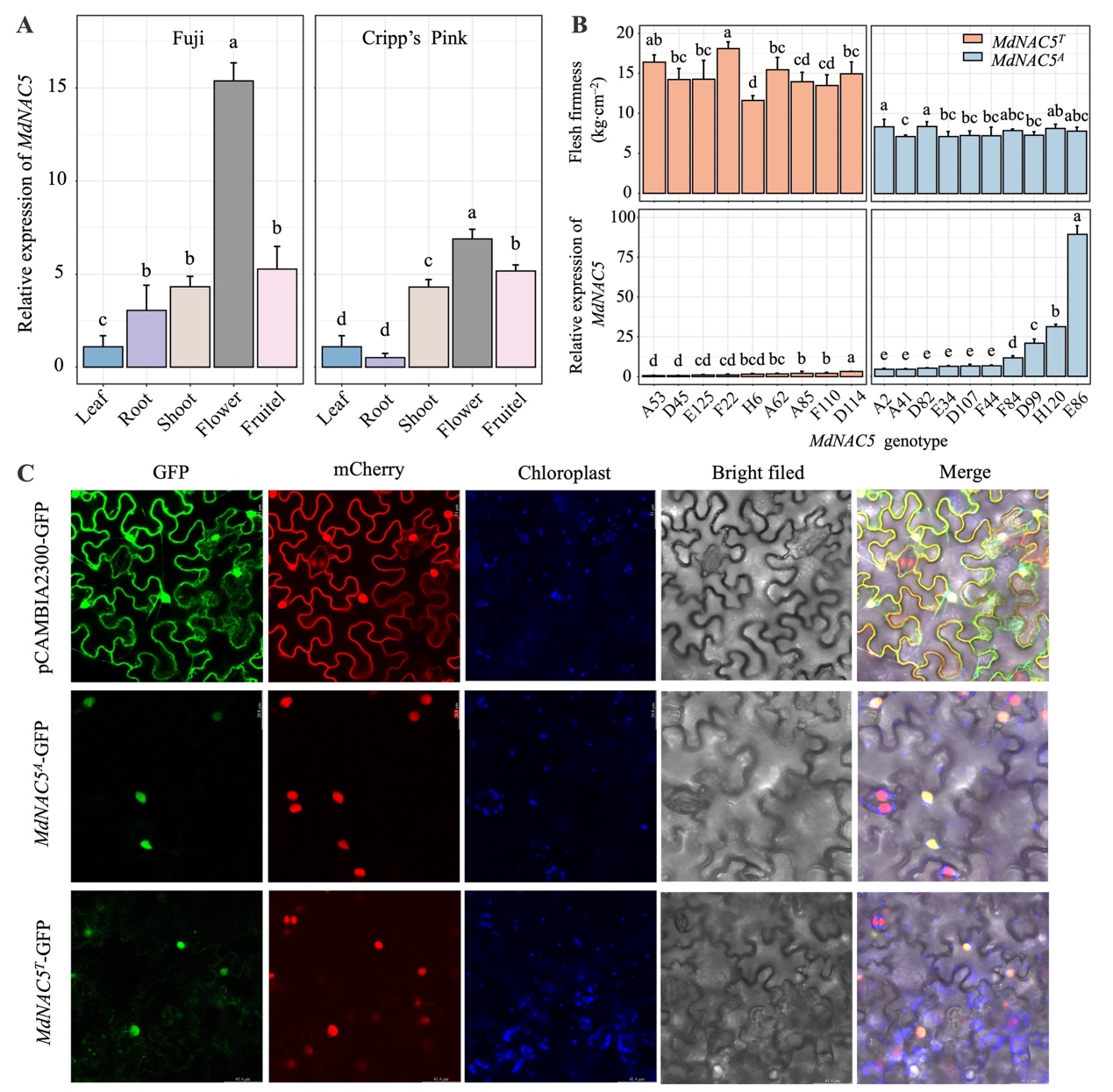
**

**Figure S8 Tissue expression profile, phenotype and subcellular localization analysis of *MdNAC5^A^* and *MdNAC5^T^*. (A)** Relative expression levels of *MdNAC5* in different tissue. **(B)** The fruit firmness and relative expression of *MdNAC5* in hybrids of homozygous genotype *MdNAC5^A^* and *MdNAC5^T^*. The bars are mean the values of three replicates (± SD), and diverse letters show differ significantly (Turkey's test, *p* < 0.05). **(C)** MdNAC5^A^ and MdNAC5^T^ subcellular localization in *N. benthamiana* leaves, indicating that they were located on the nucleus. Scale bars, 20 μm.

**
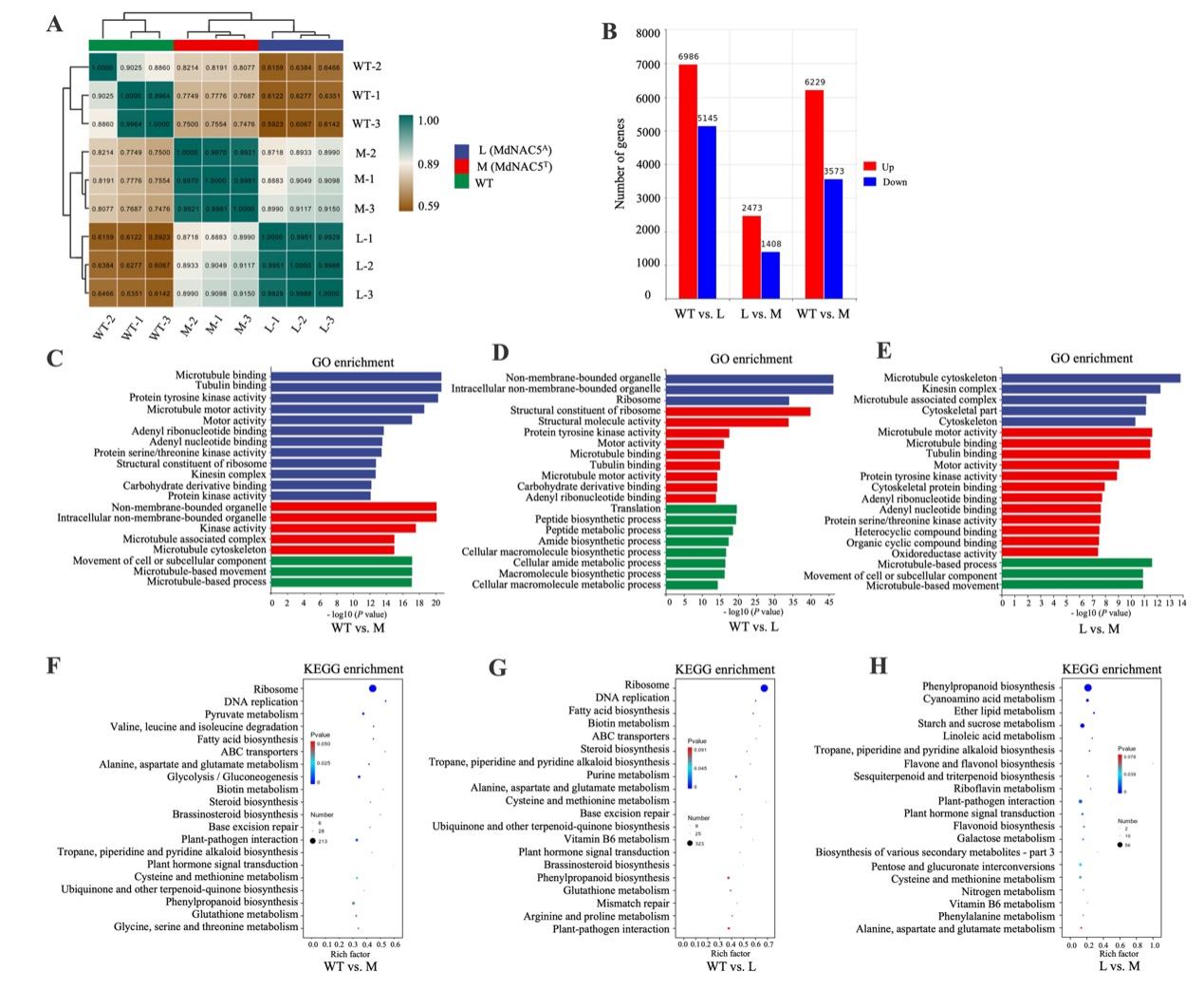
**

**Figure S9 The DEGs analysis of *MdNAC5* transgenic apple calli. (A)** Correlation of gene expression level among samples of WT, OE-*MdNAC5^A^* (M), OE-*MdNAC5^T^* (L). **(B)** Up-regulation and down-regulation of genes in apple calli between different groups. (**C–E)** GO terms analysis of DEG between different groups. **(F–H)** KEGG pathway analysis of DEGs between different groups.

**
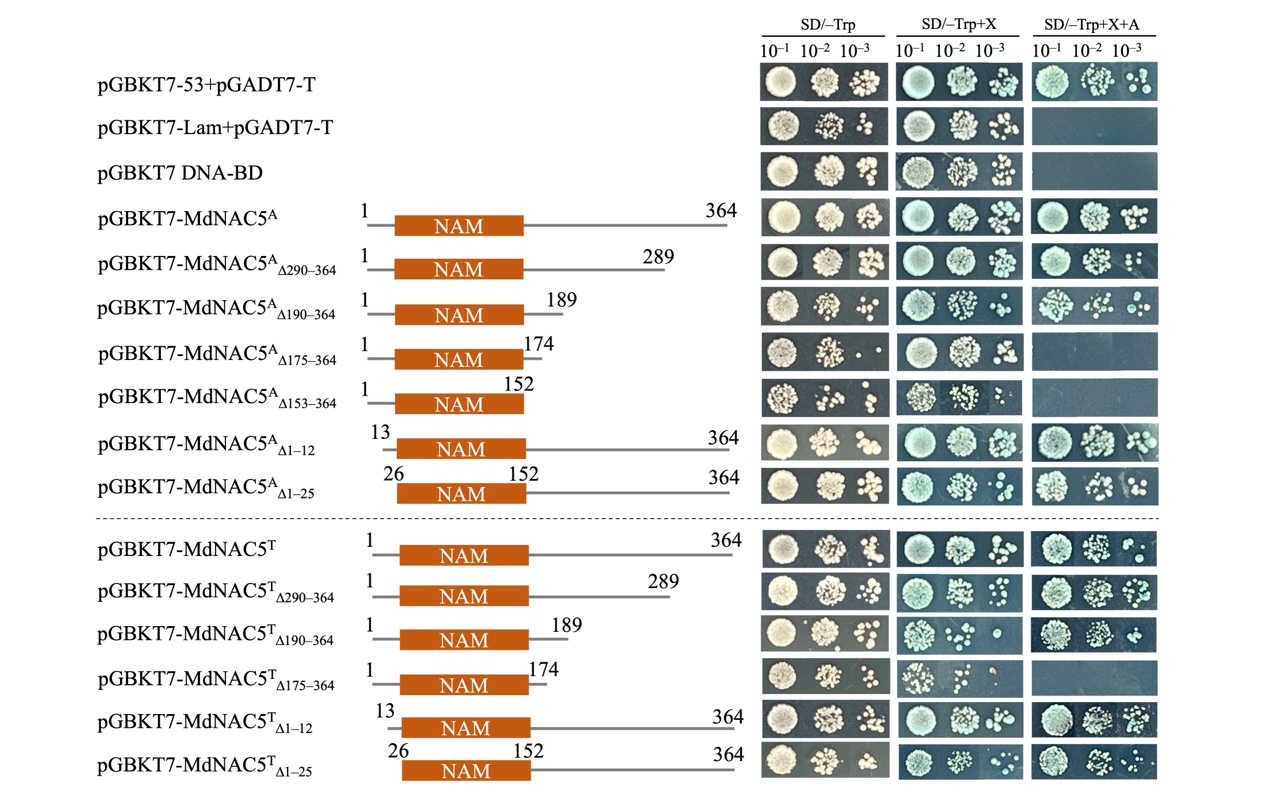
**

**Figure S10 The process of obtaining transgenic tomatoes. (A)** The process of obtaining cv Ailsa Craig (AC) and nor mutants of *MdNAC5* transgenic tomato. **(B, C)** Relative expression levels of *MdNAC5* in leaves of transgenic tomato. **(D)** Identification of protein expression level in *MdNAC5* transgenic tomato. The bars are mean the values of three replicates (± SD), and diverse letters show differ significantly (Turkey's test, *p* < 0.05).

**
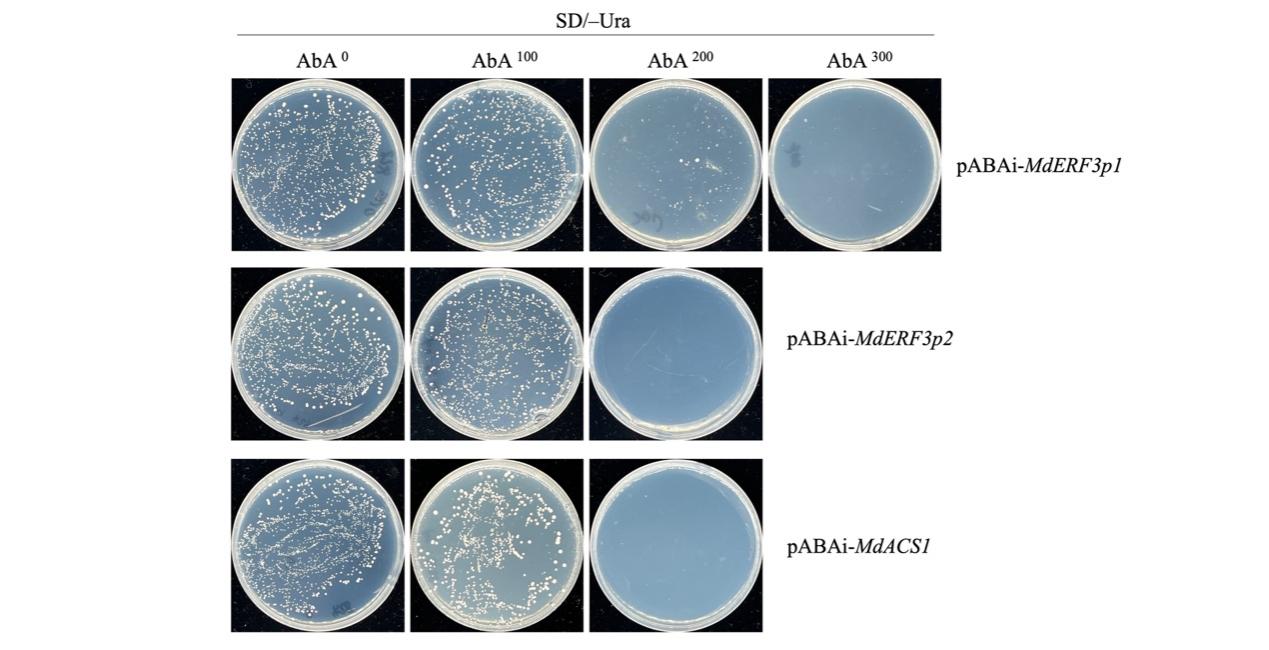
**

**Figure S11 Screening of minimal AbA concentration of pAbAi-*MdERF3p1*, pAbAi-*MdERF3p2* and *MdACS1* bait strain.**

**
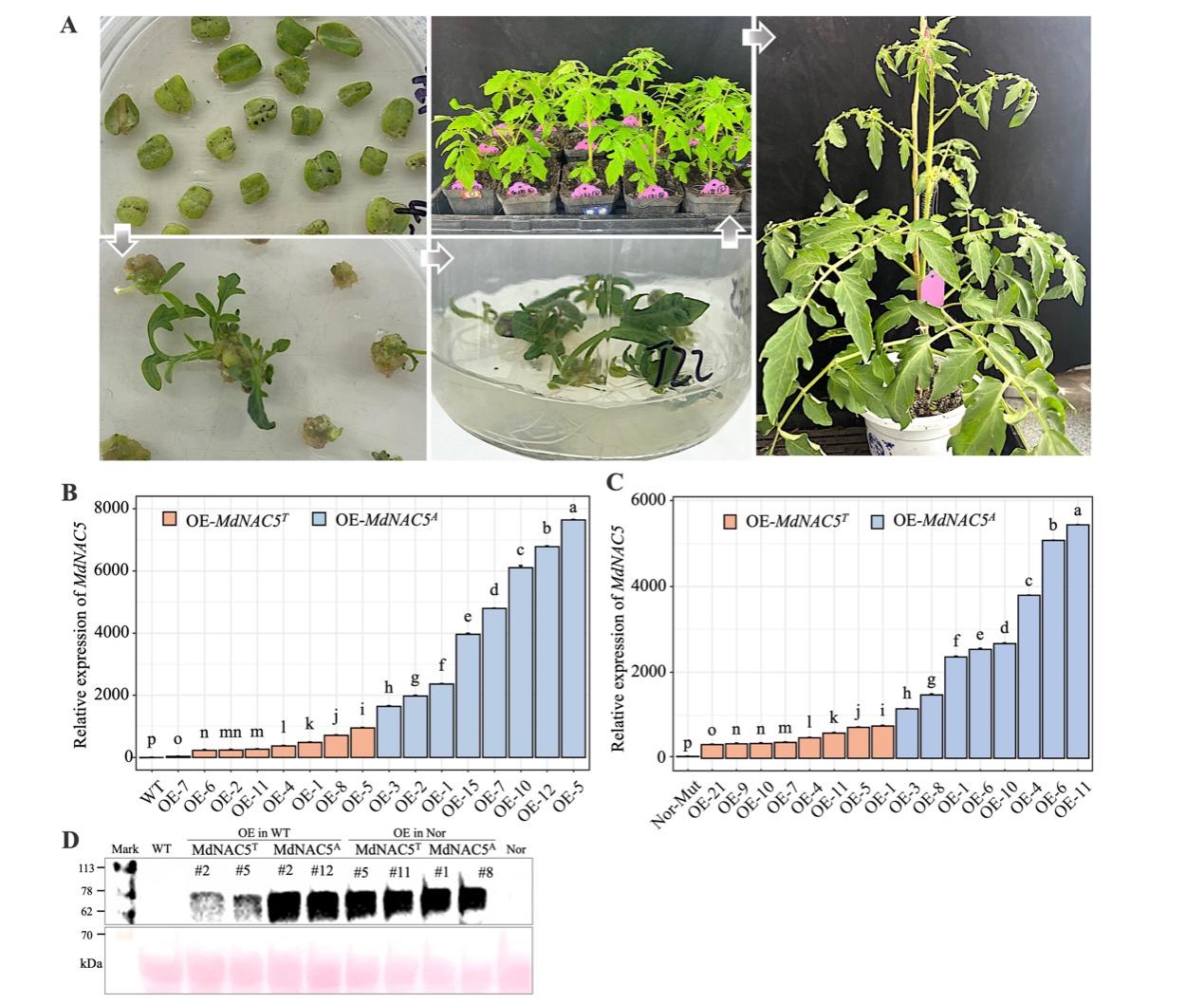
**

**Figure S12 Verification of MdNAC5^A^ and MdNAC5^T^ transcriptional self-activation.** Positive control: pGBKT7-53 + pGADT7-T; negative control: pGBKT7-Lam + pGADT7-T. X: supplemented with 40 μg·mL^–1^ X-α-Gal; A: supplemented with 200 ng·mL^–1^ of AbA.


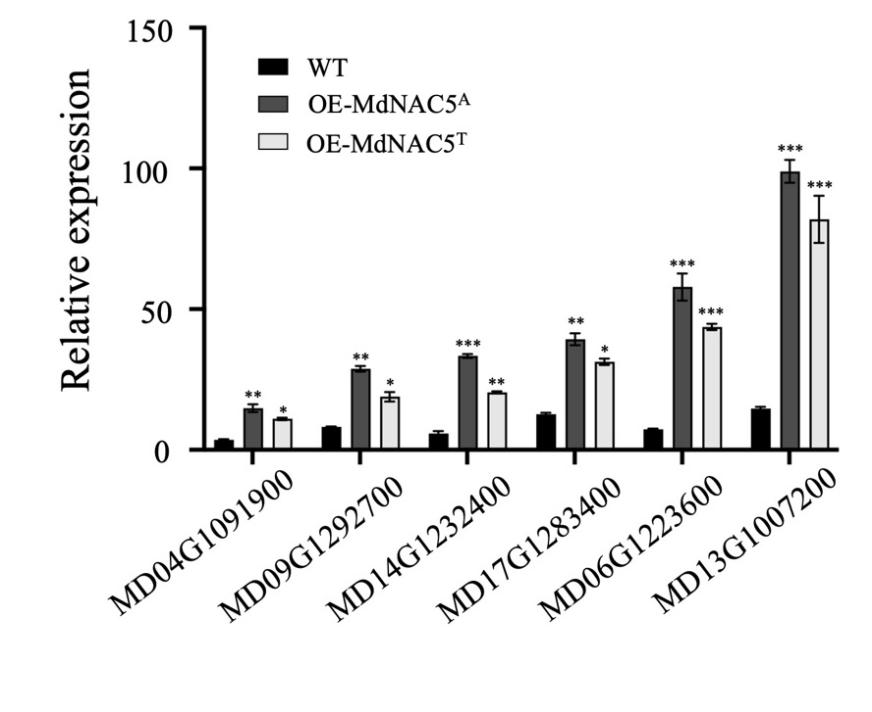


**Figure S13 Relative expression of S-adenosylmethionine synthase gene family in apple calli.** Values are means of three replicates ± SD (Turkey's test, *** *P* < 0.001, ** *P* < 0.01, **P* < 0.05).

**See another separate Excel file for tables 1–13.**

**Table S1** Phenotypic distribution of 5 fruit-related traits in 935 F_1_ hybrids from hybridization of apple ‘Fuji’ and ‘Cripp's Pink’.

**Table S2** The sequencing data for F_1_ hybrids.

**Table S3** Statistical sequencing data for the two parents and all F_1_ hybrids.

**Table S4** Statistics of two parents and F_1_ hybrids mapping with reference genome, and their coverage depth and coverage ratio.

**Table S5** SNP statistics detected between parents of ‘Fuji’ and ‘Cripp's Pink’.

**Table S6** Description of the basic characteristics on 17 chromosomes of female and male genetic map.

**Table S7** QTLs test results of five quantitative traits in apple.

**Table S8** Statistical sequencing data for the two pools.

**Table S9** Statistics of two pools mapping with reference genome.

**Table S10** Filtering Statistics for SNPs of two pools.

**Table S11** Positioning results of firm/soft flesh firmness in apple.

**Table S12** Annotation information of candidate genes based on the common interval.

**Table S13** The list of primer and gene sequences in this study.
