# Supplementary figures and images for: Natural variation in MdNAC5 contributes to fruit firmness and ripening divergence in apple
Source: Hortic Res. 2024 Oct 8;12(1):uhae284. doi: 10.1093/hr/uhae284 (PMC11758708; doi:10.1093/hr/uhae284)

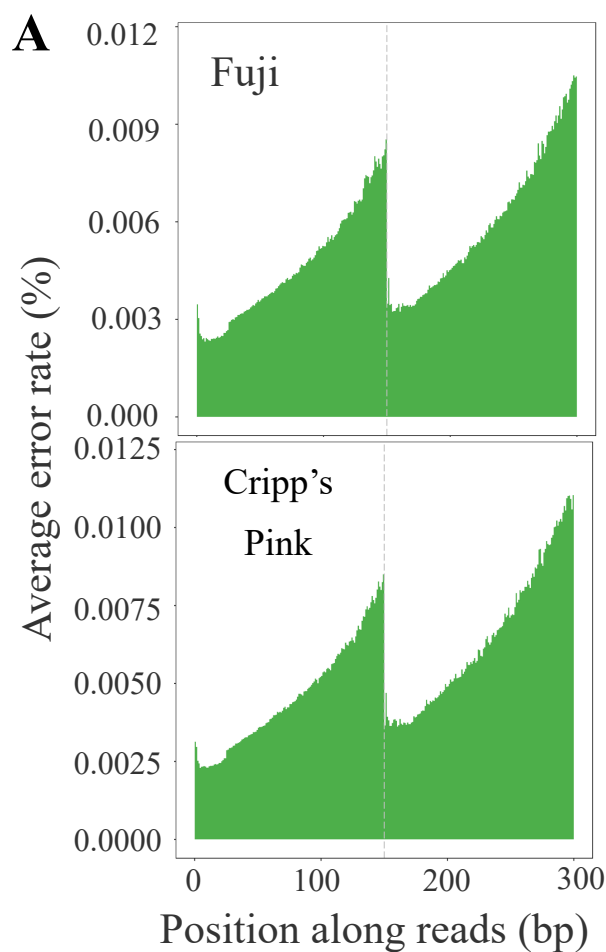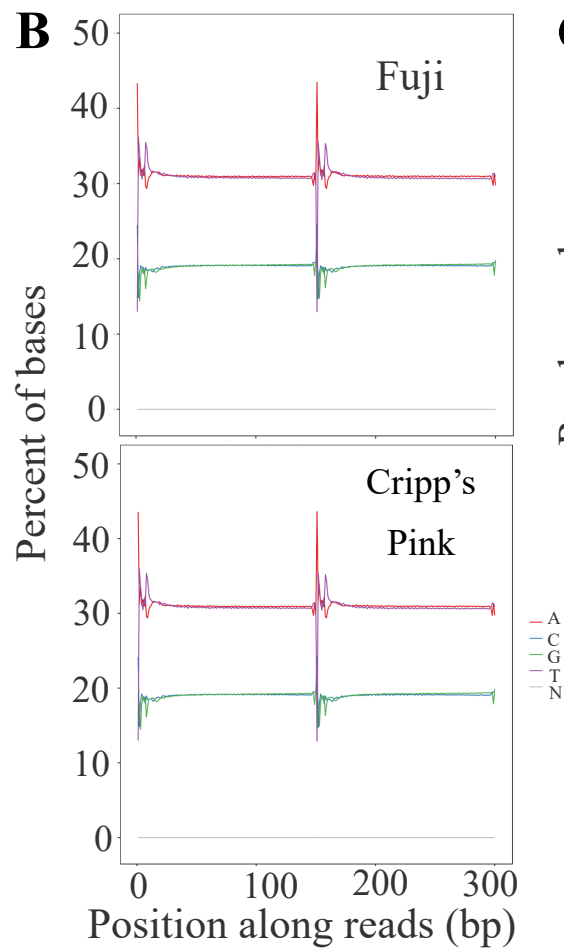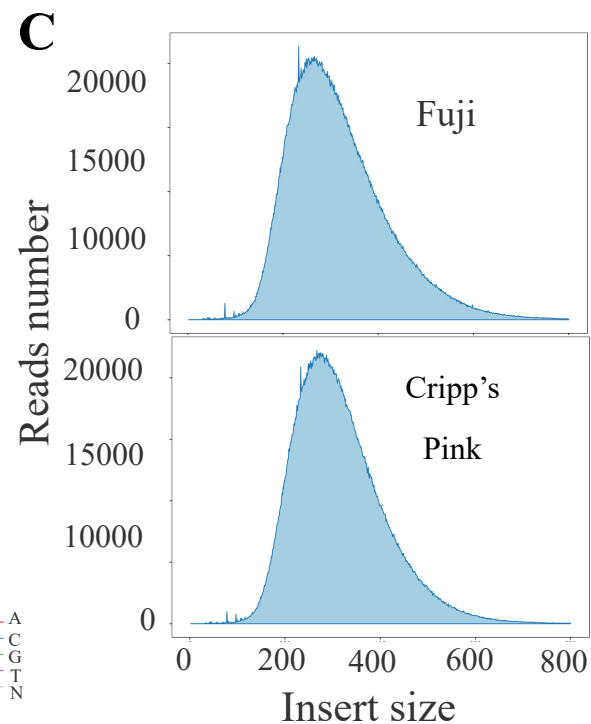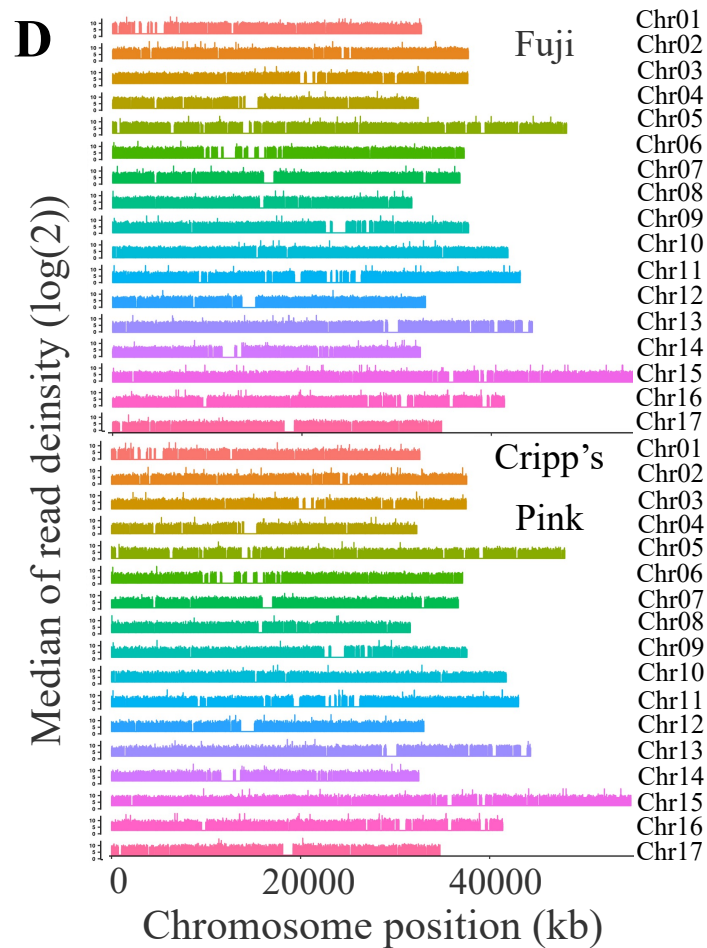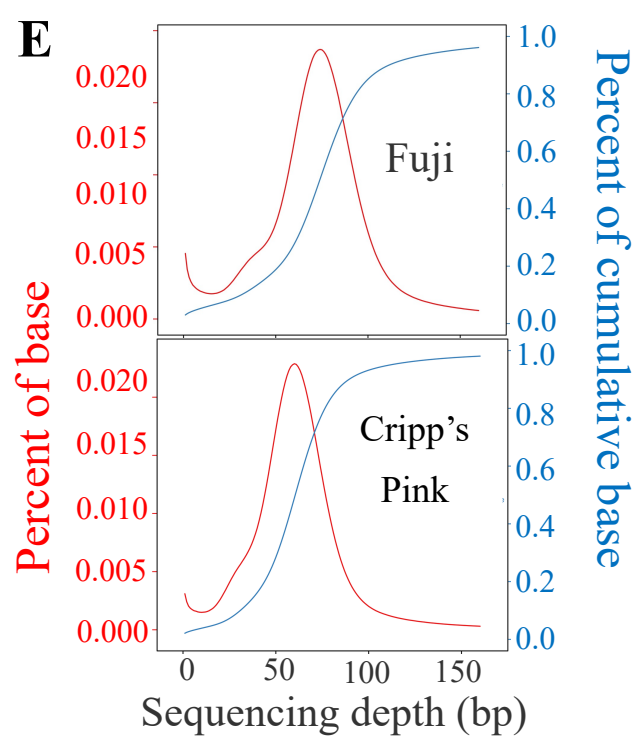

Supplement: Web_Material_uhae284 [file web_material_uhae284.zip › Figure S1.pdf]

**A**

Genetic Map

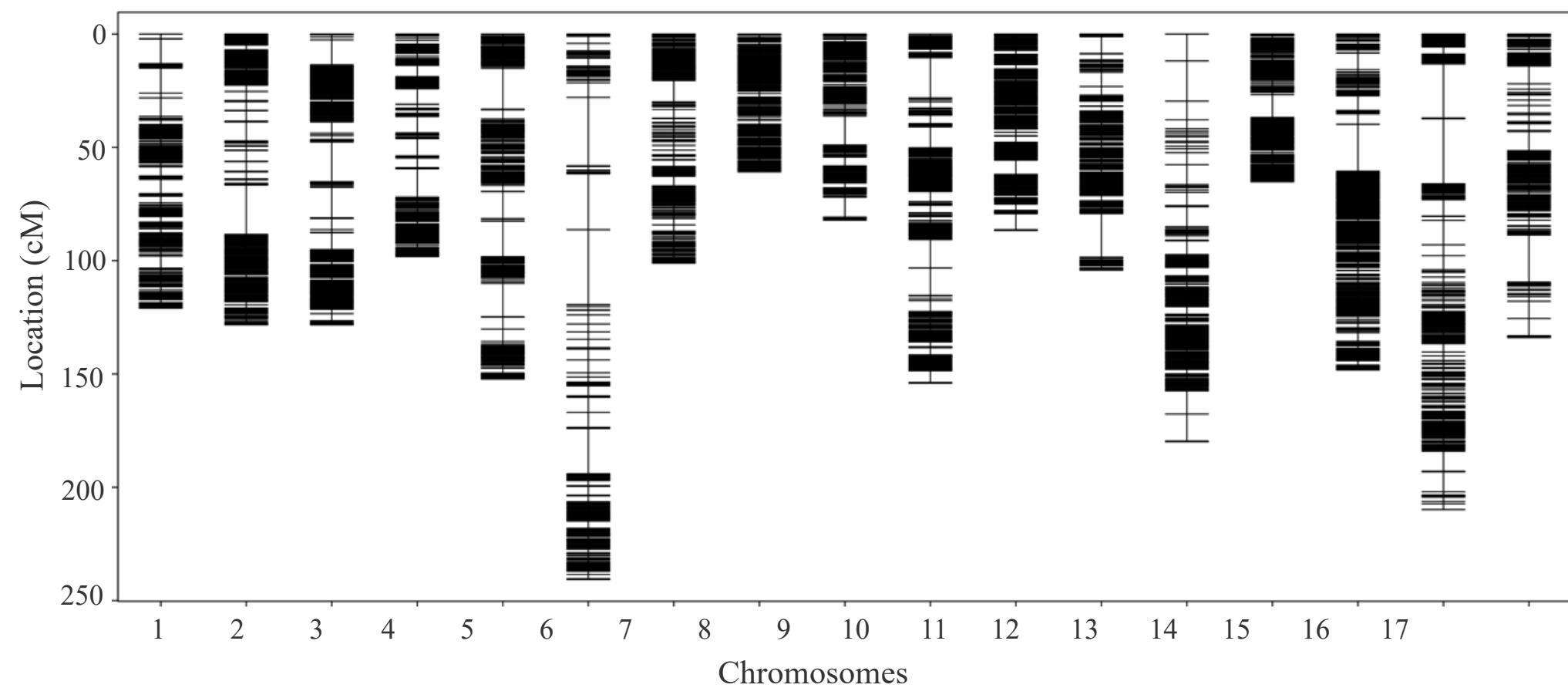**B**

Genetic Map

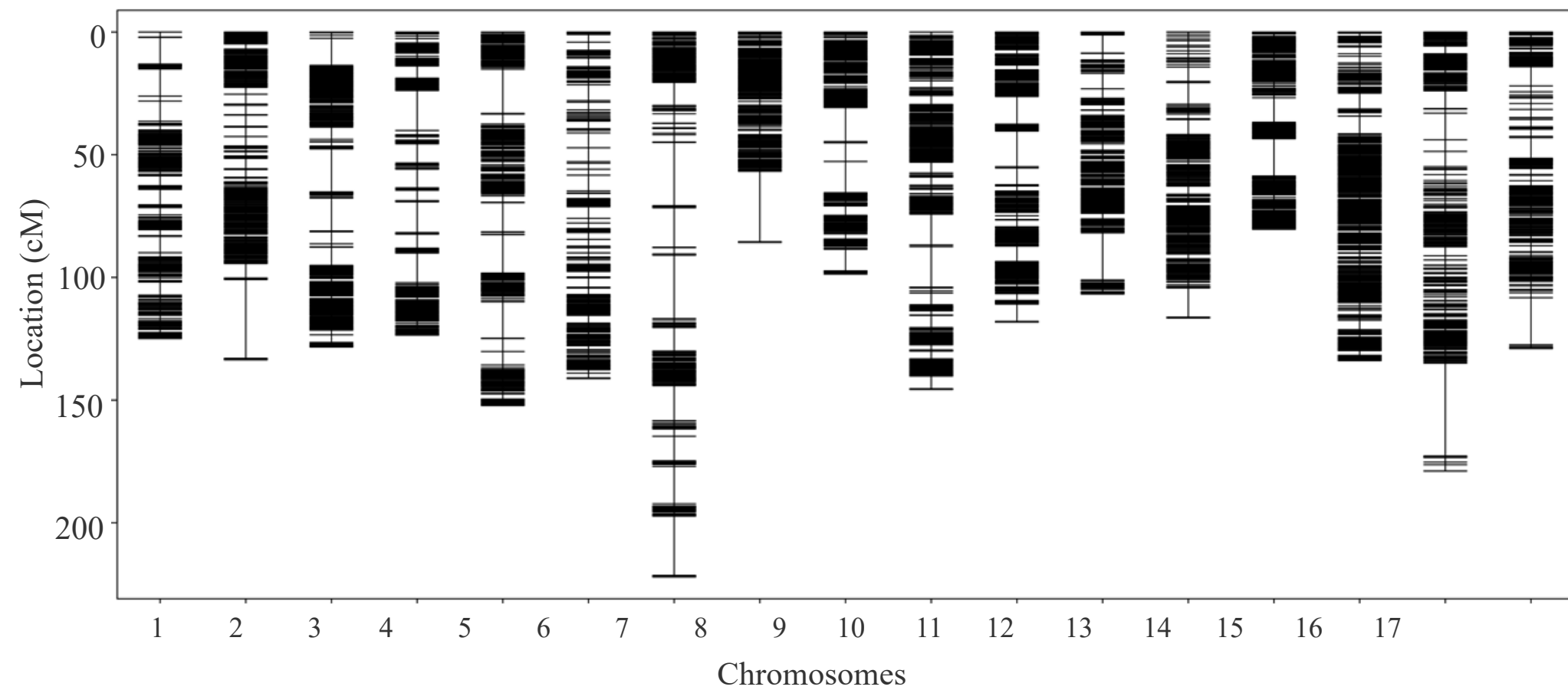

Supplement: Web_Material_uhae284 [file web_material_uhae284.zip › Figure S2.pdf]

**A**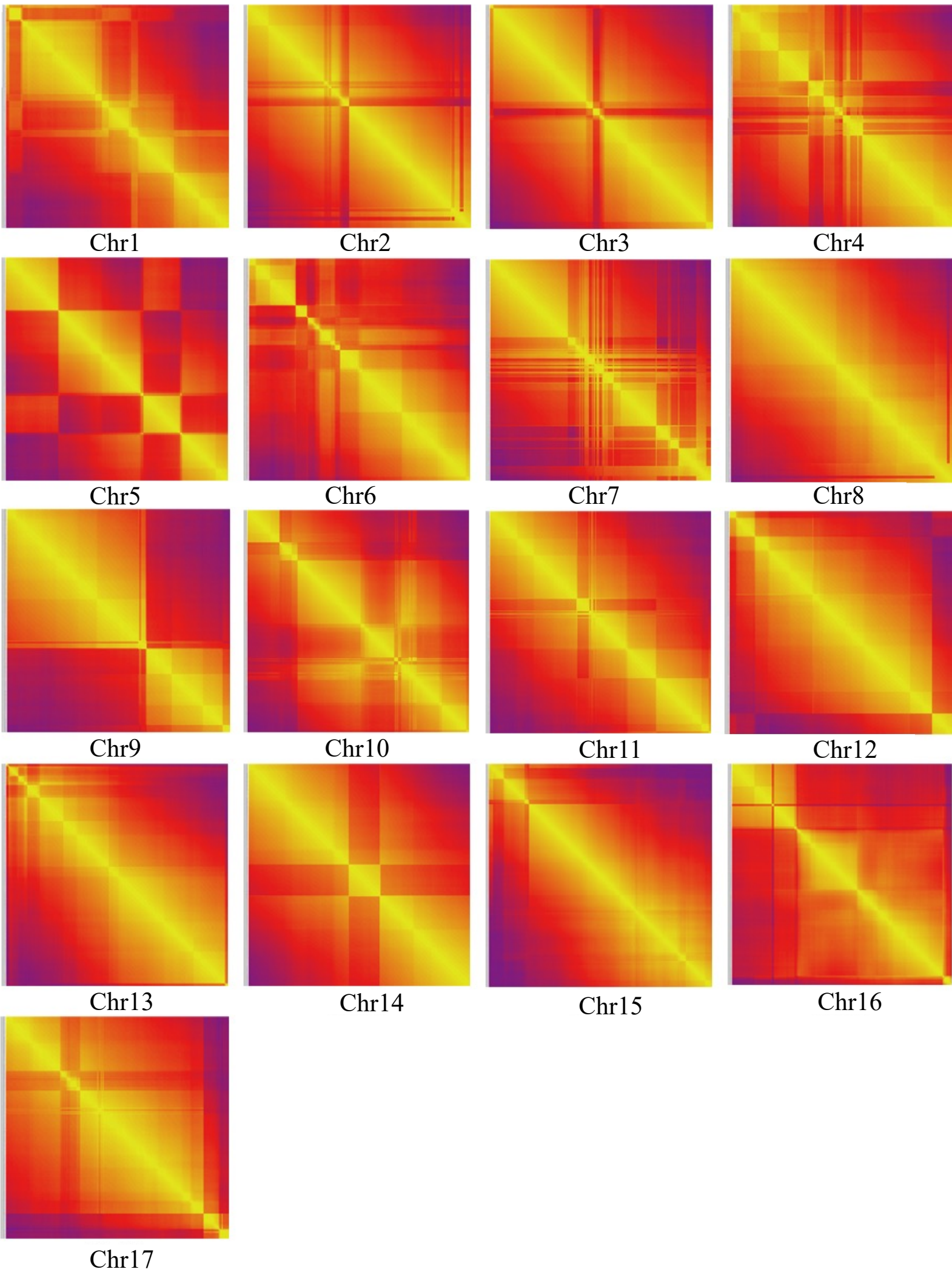**B**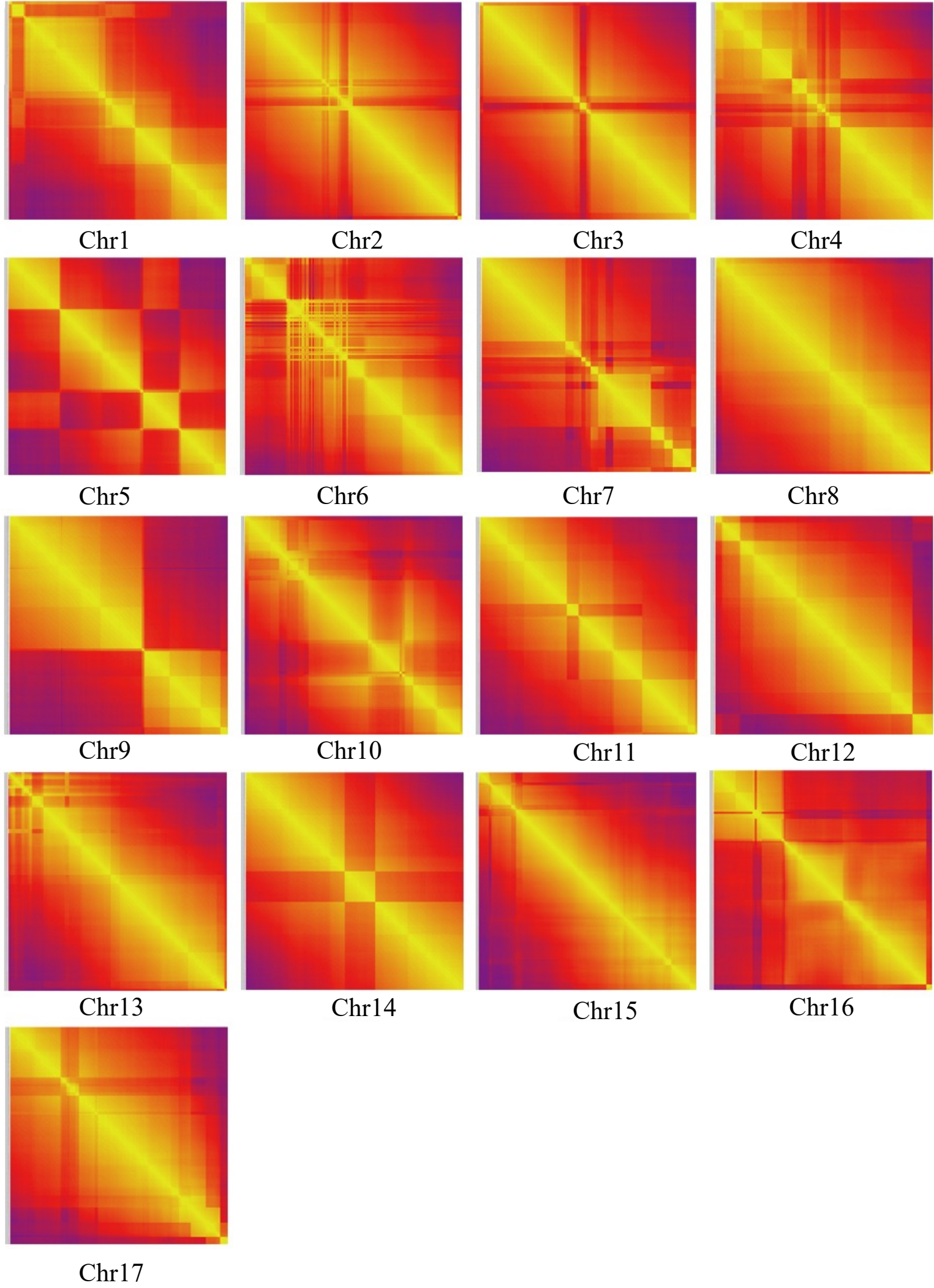

Supplement: Web_Material_uhae284 [file web_material_uhae284.zip › Figure S3.pdf]

**A**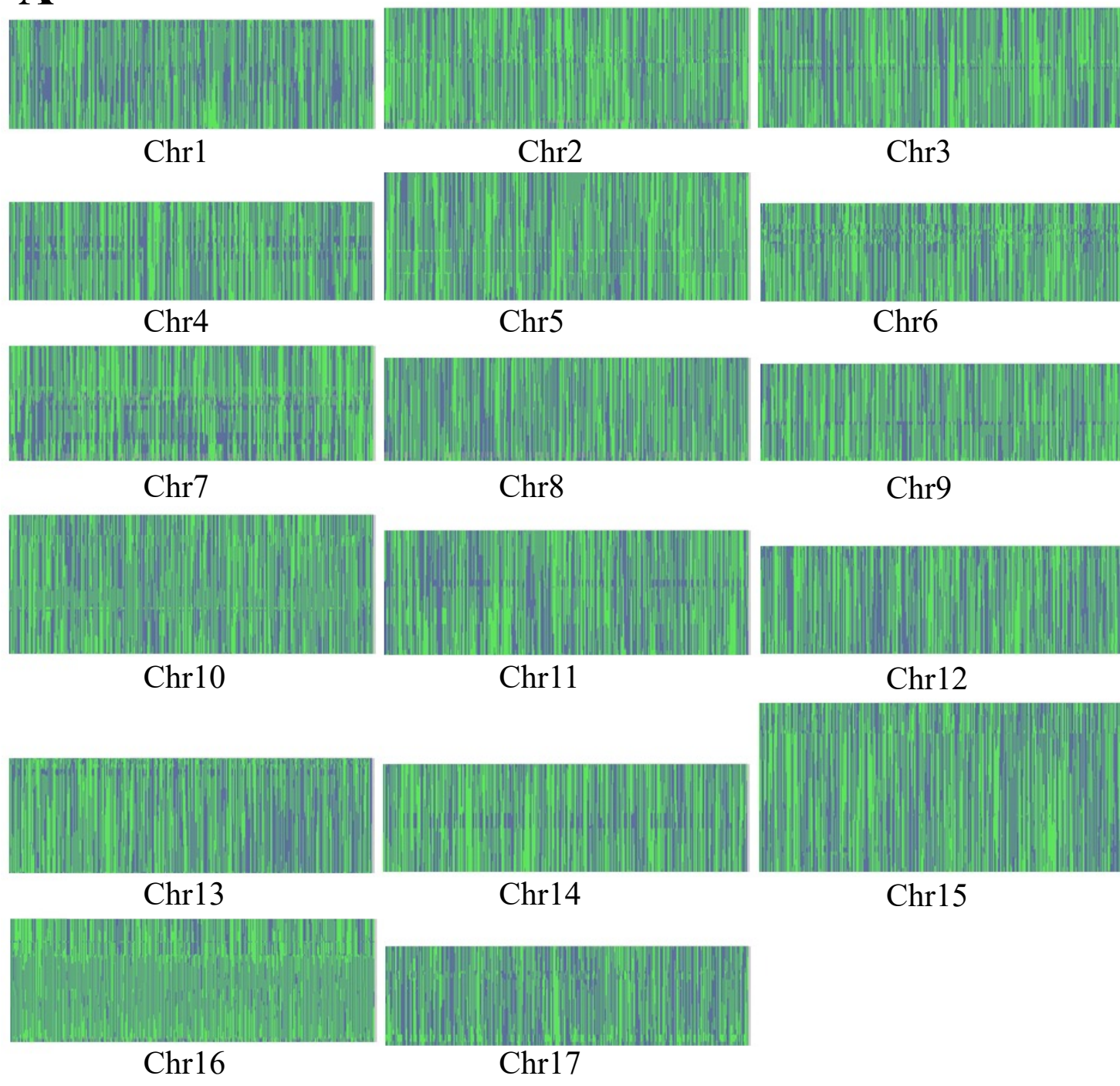**B**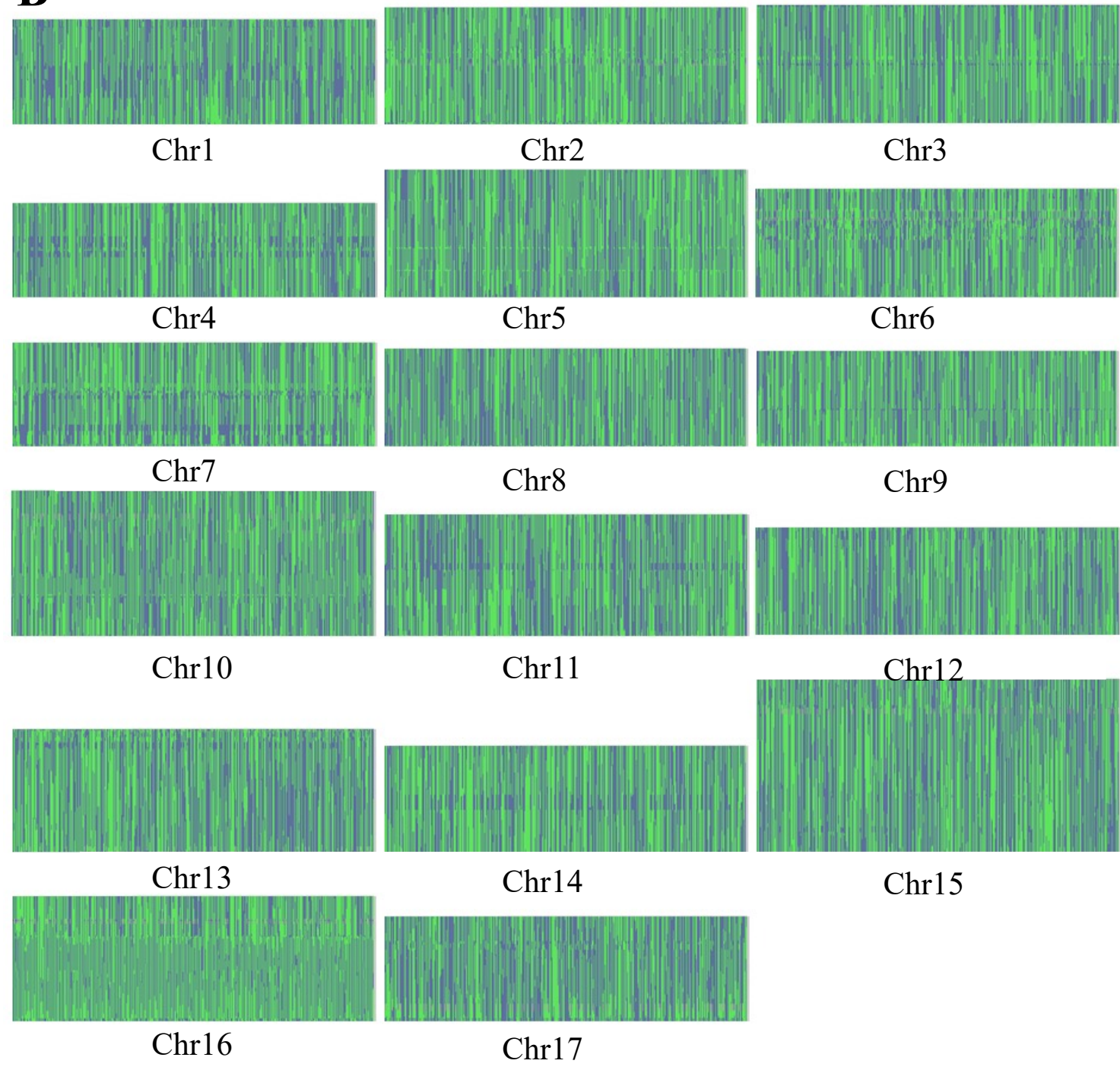**C**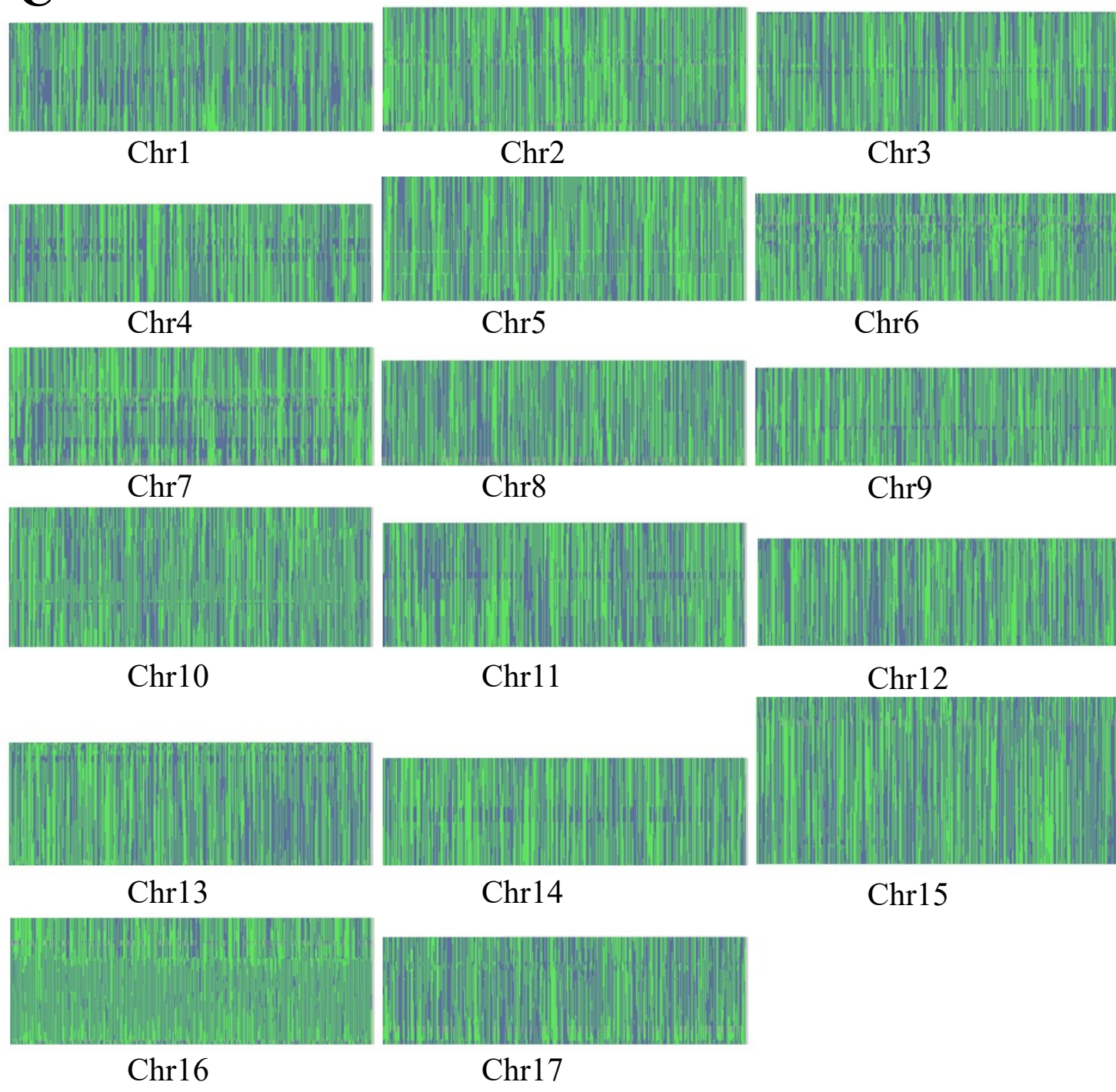

Supplement: Web_Material_uhae284 [file web_material_uhae284.zip › Figure S4.pdf]

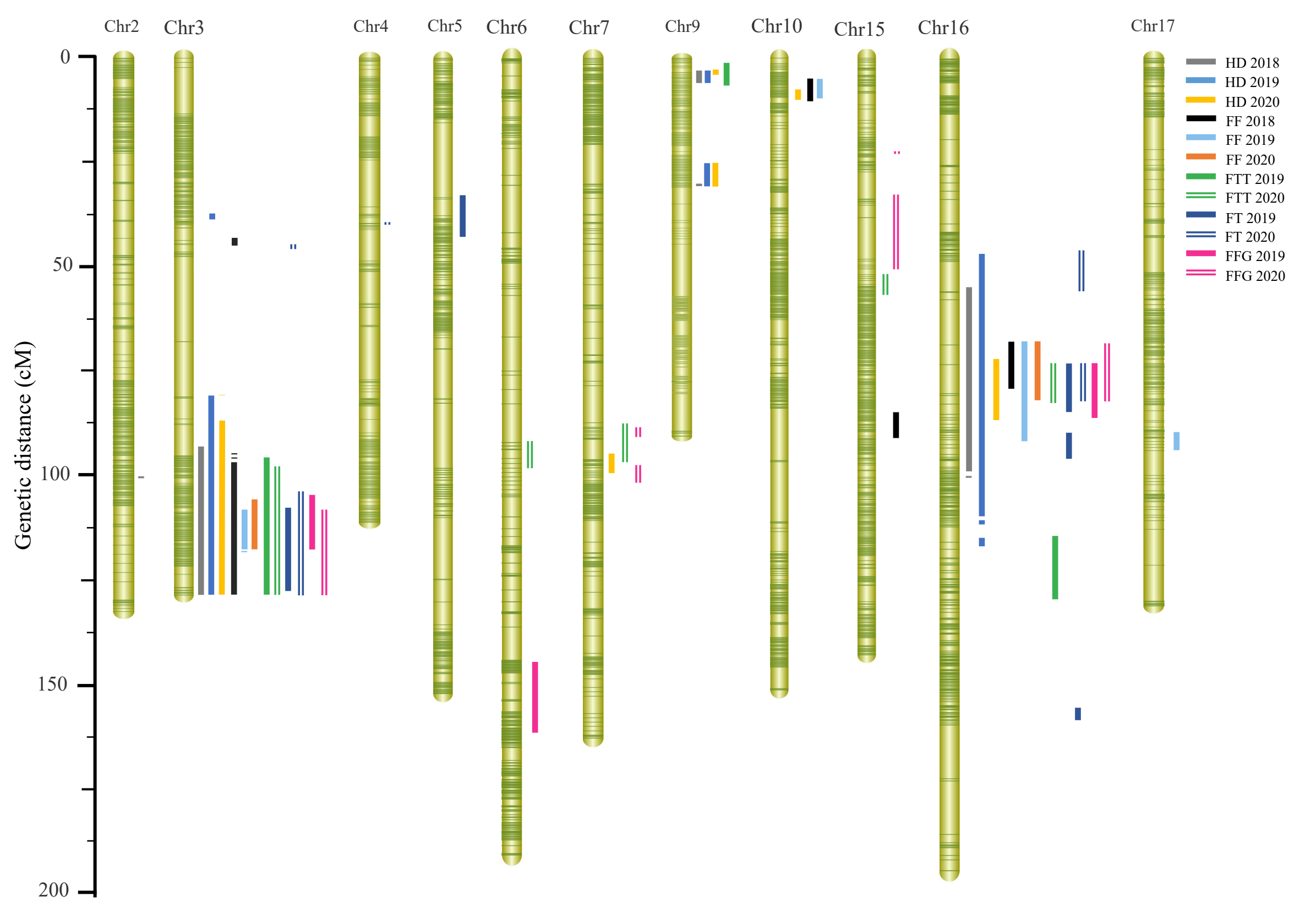

Supplement: Web_Material_uhae284 [file web_material_uhae284.zip › Figure S5.pdf]

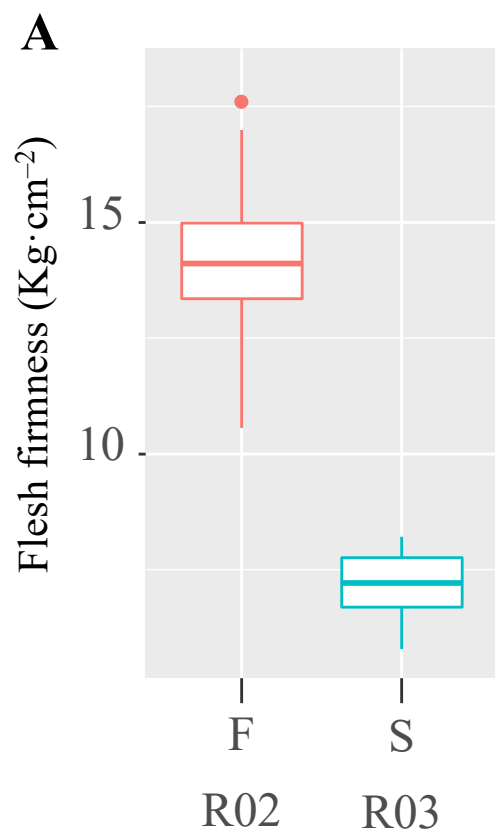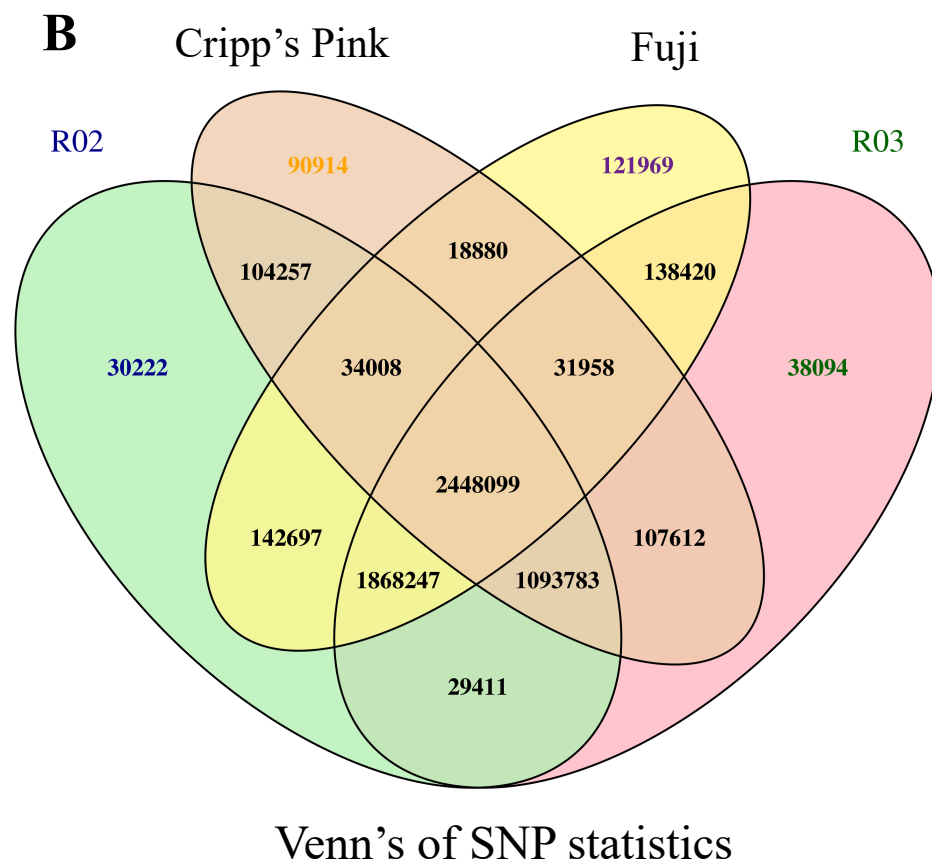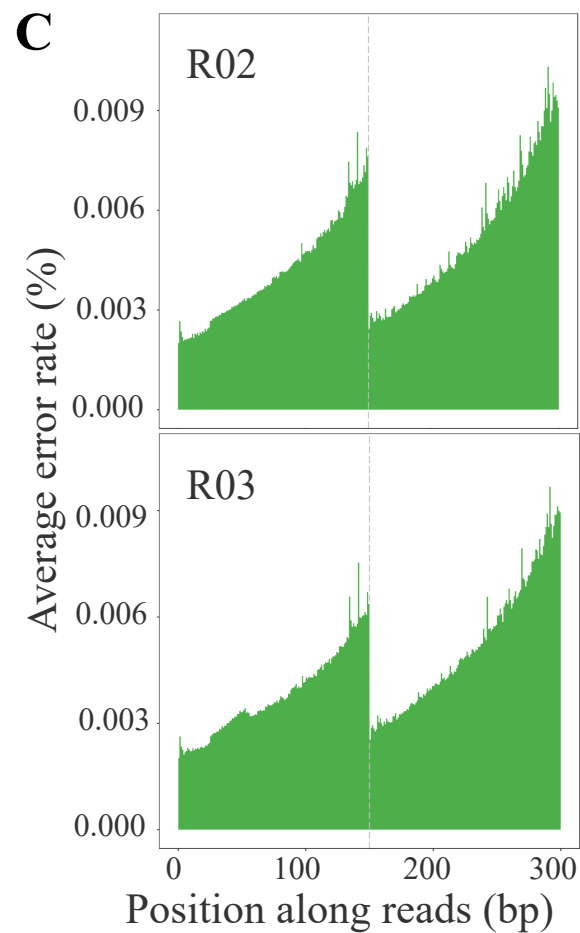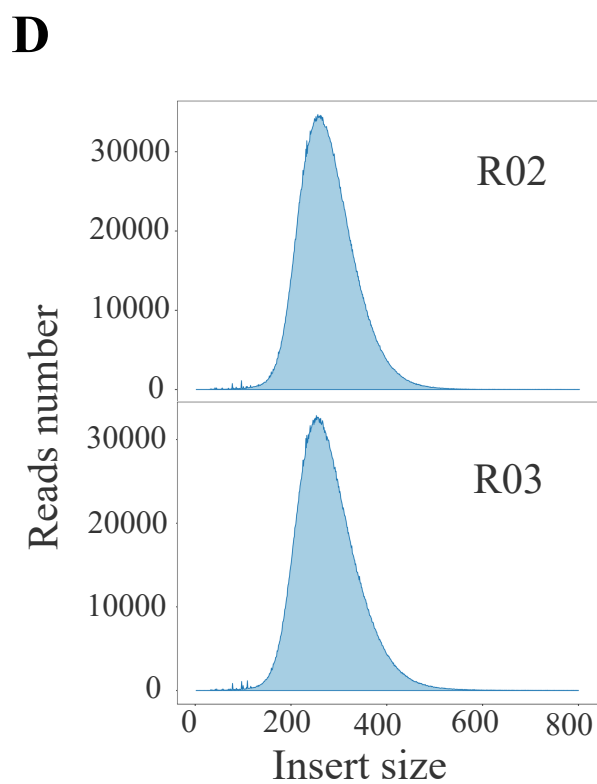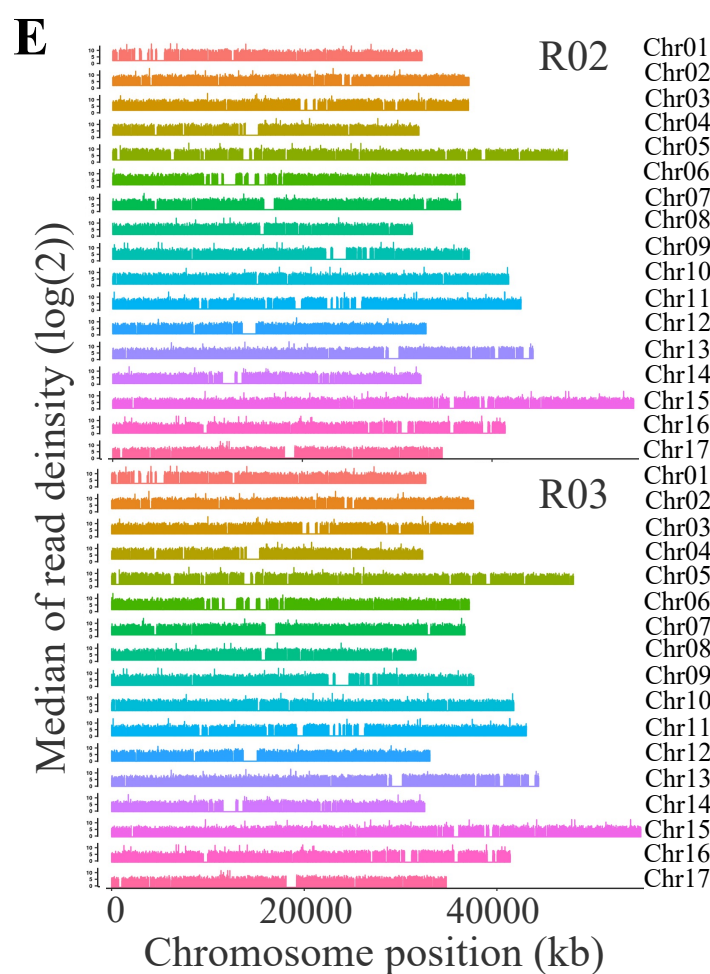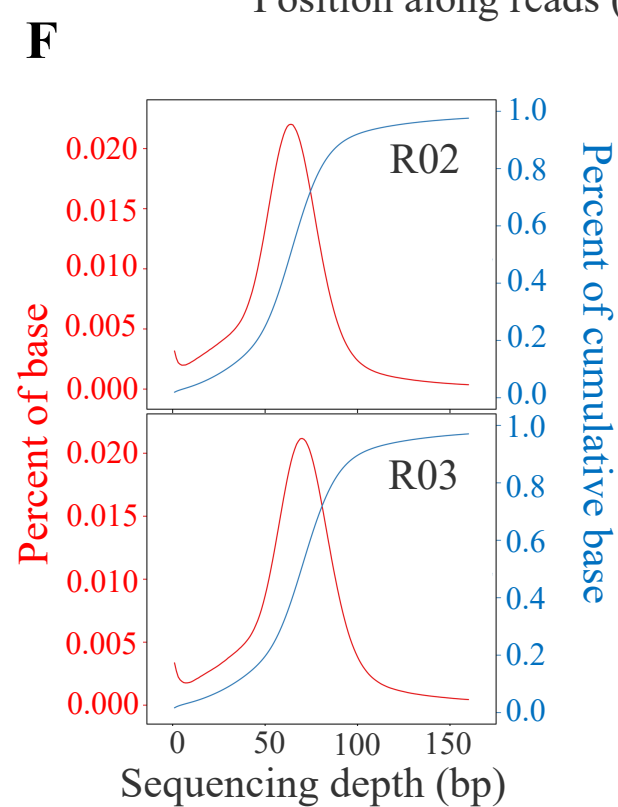

Supplement: Web_Material_uhae284 [file web_material_uhae284.zip › Figure S6.pdf]

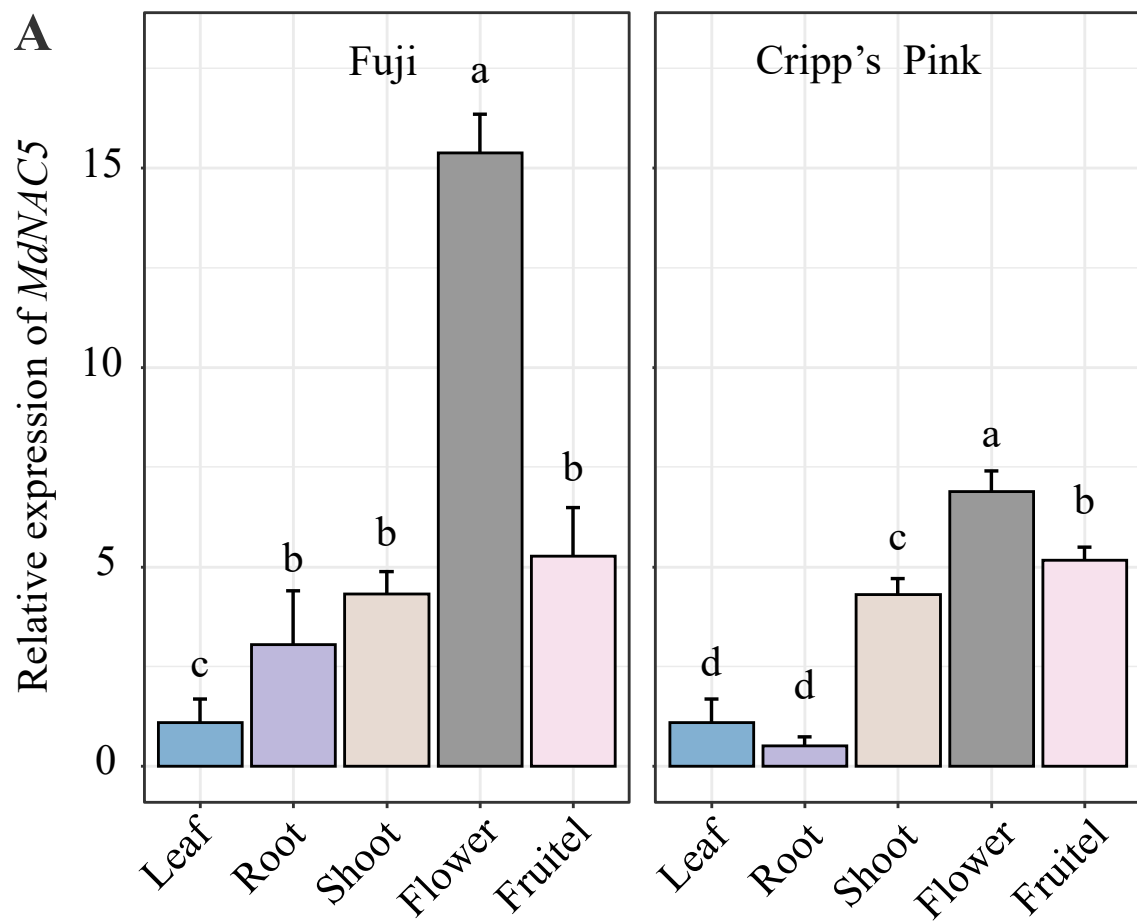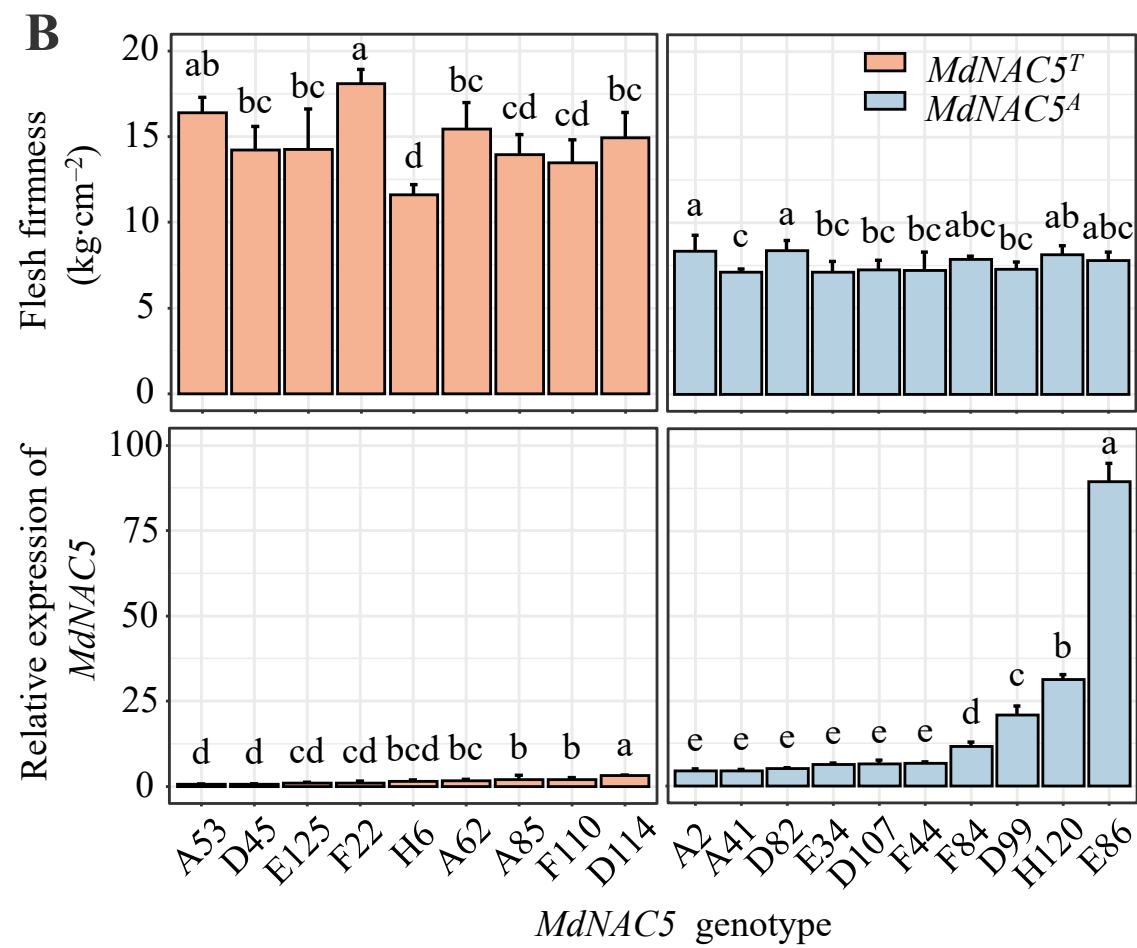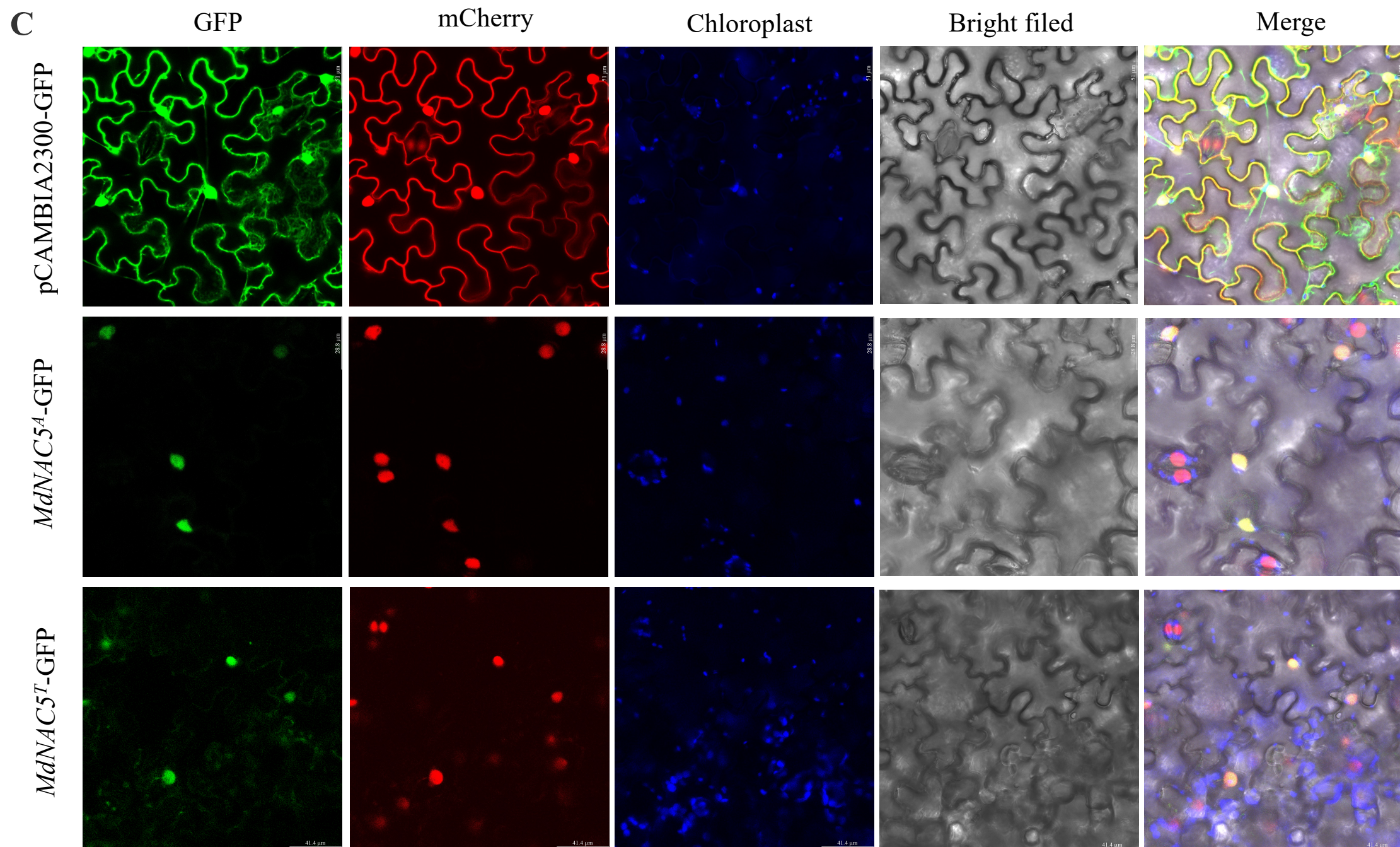

Supplement: Web_Material_uhae284 [file web_material_uhae284.zip › Figure S8.pdf]

A

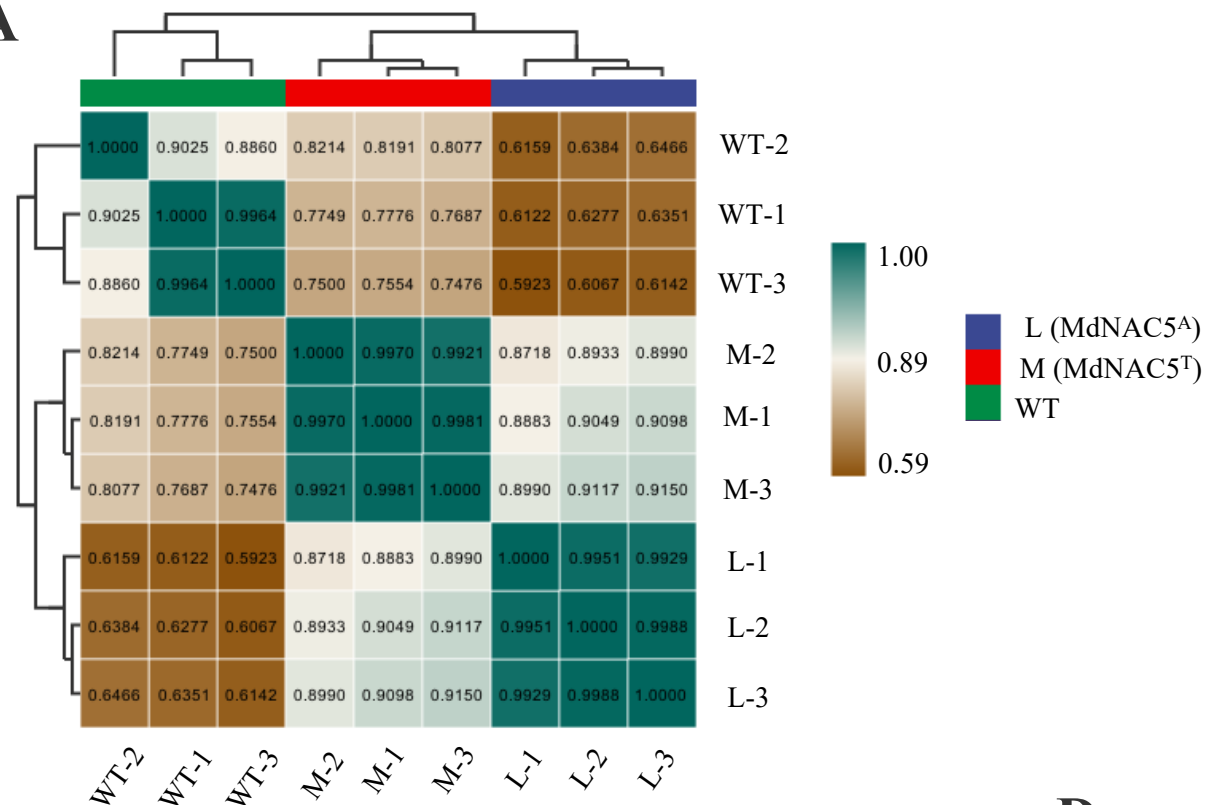

B

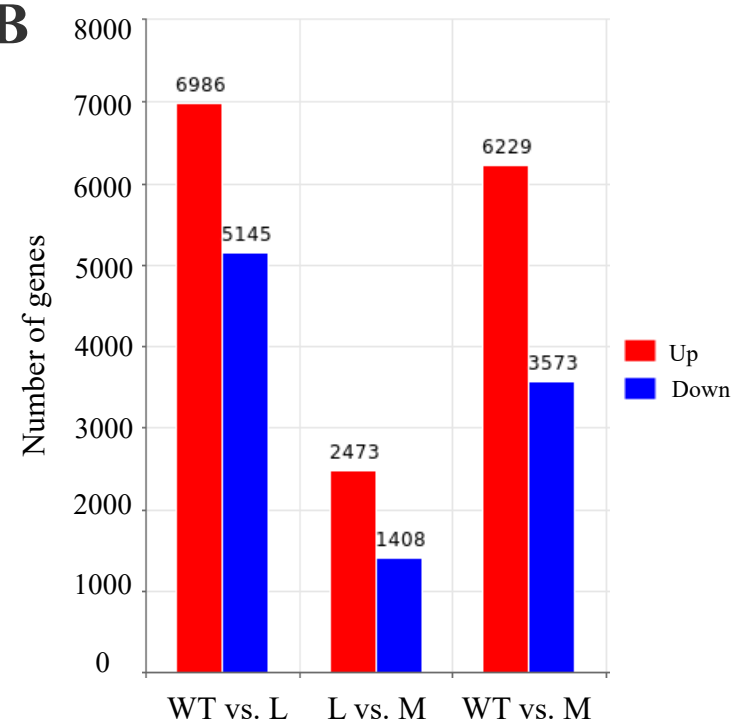

C

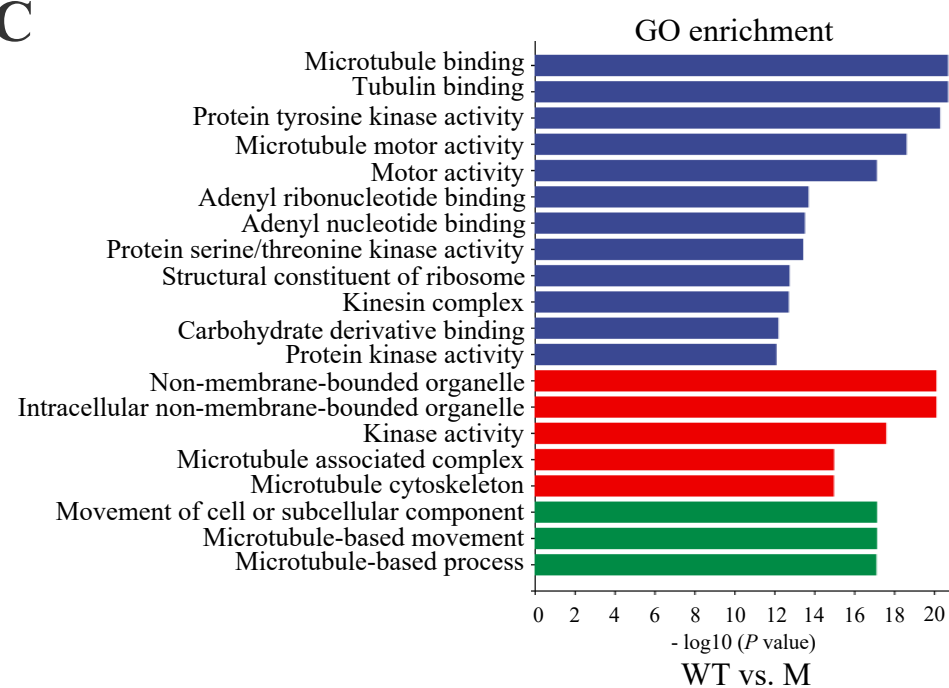

D

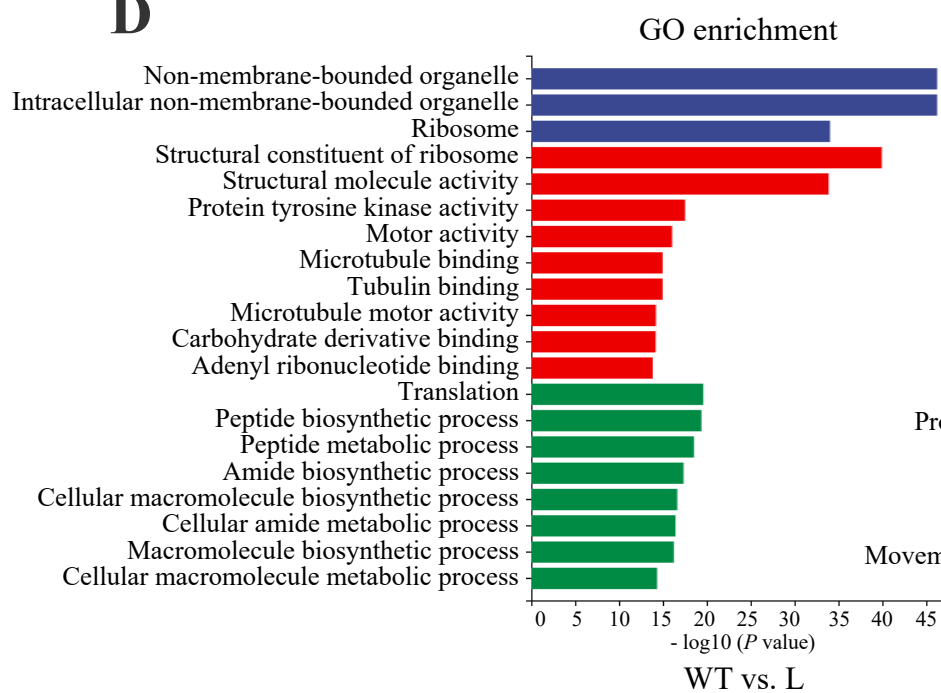

E

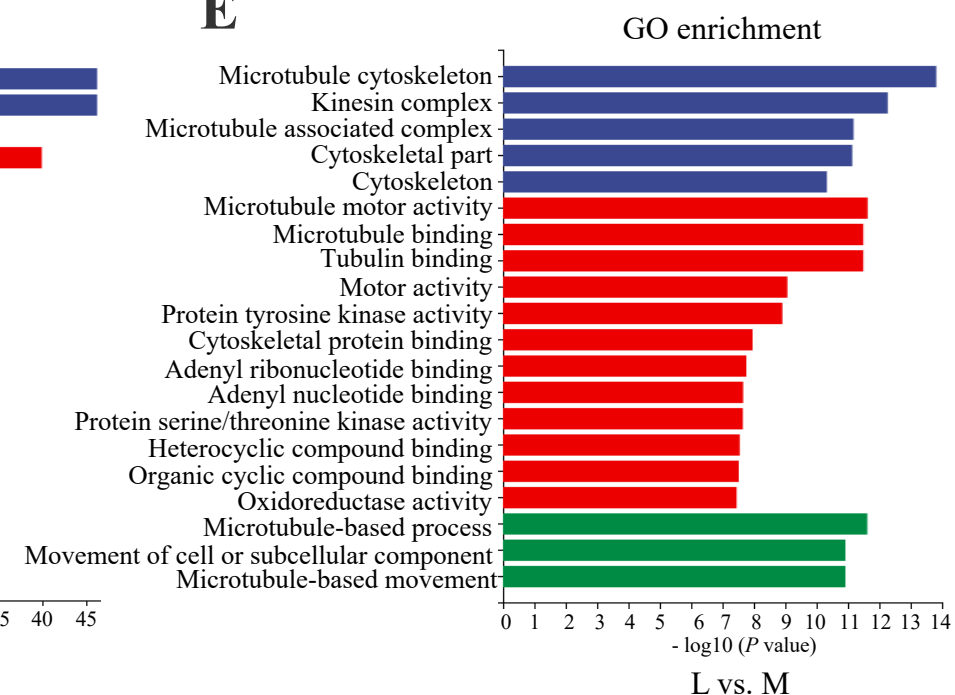

F

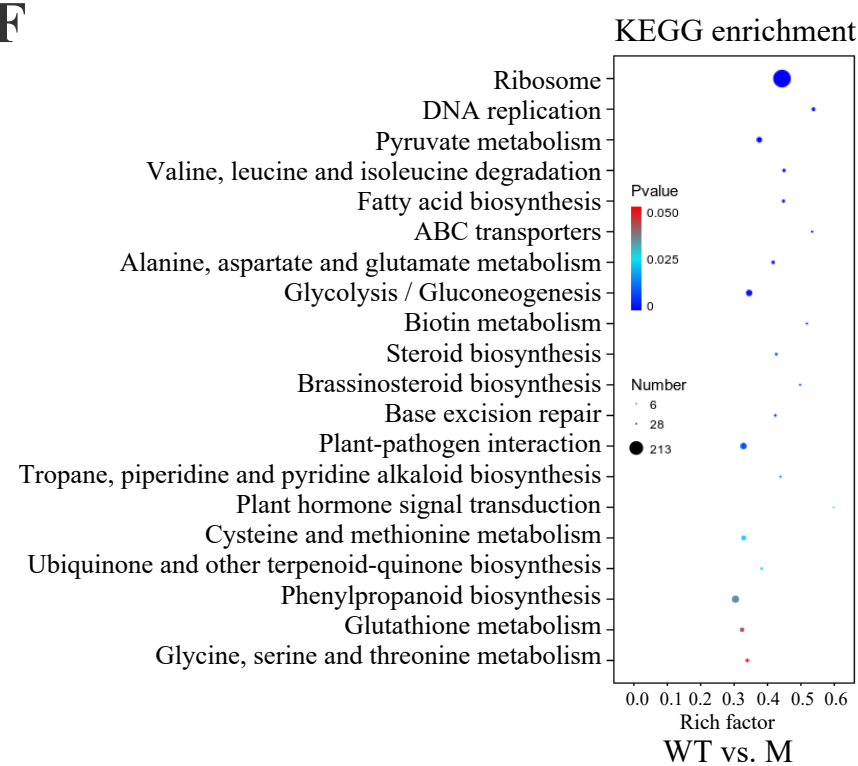

G

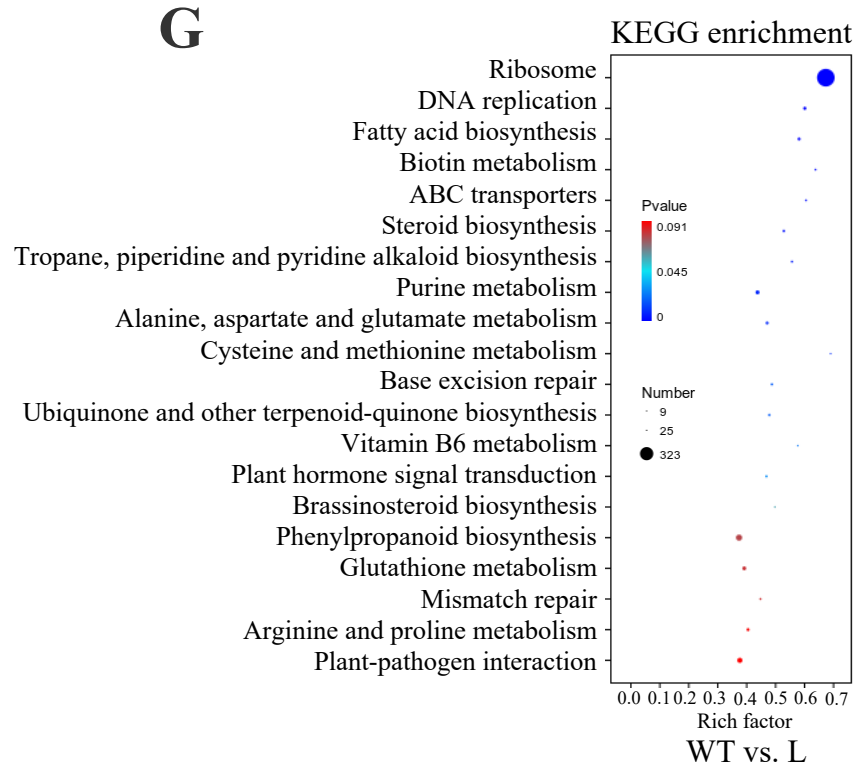

H

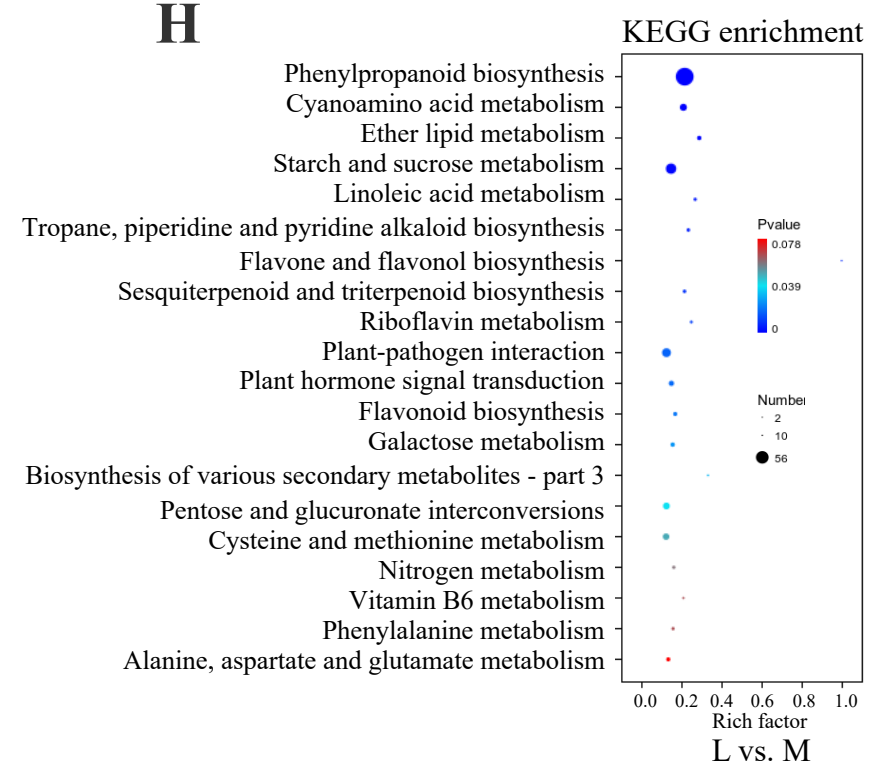

Supplement: Web_Material_uhae284 [file web_material_uhae284.zip › Figure S9.pdf]

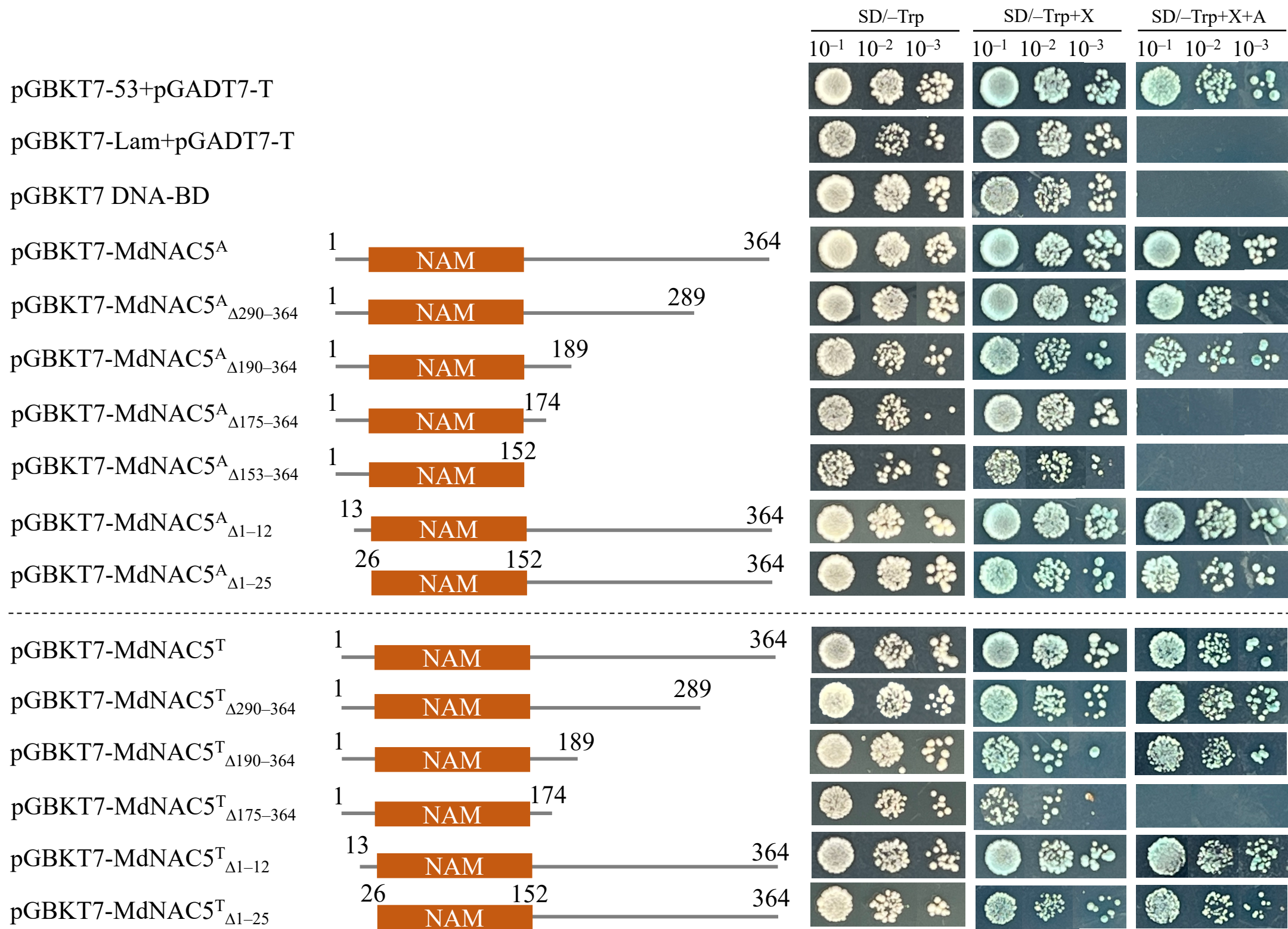

Supplement: Web_Material_uhae284 [file web_material_uhae284.zip › Figure S10.pdf]

SD/-Ura

AbA<sup>0</sup>

AbA<sup>100</sup>

AbA<sup>200</sup>

AbA<sup>300</sup>

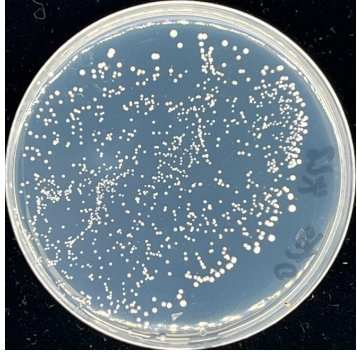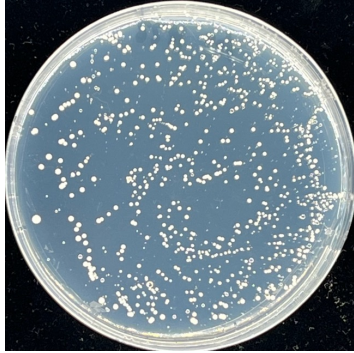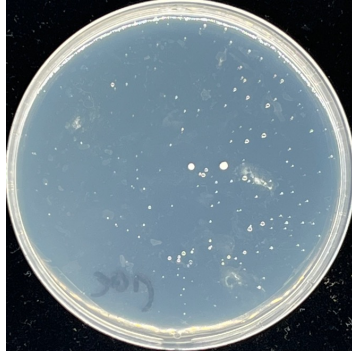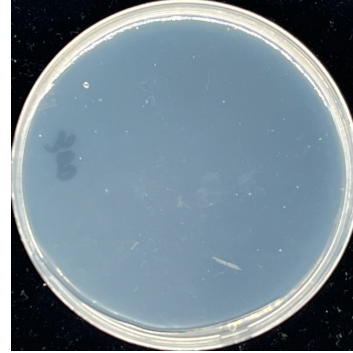

*pABAi-MdERF3p1*

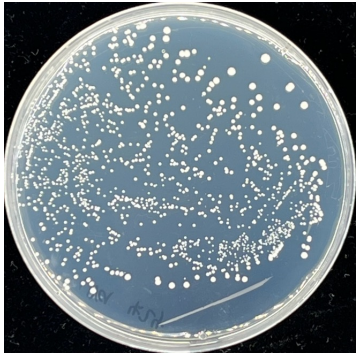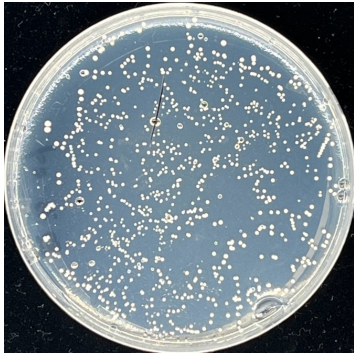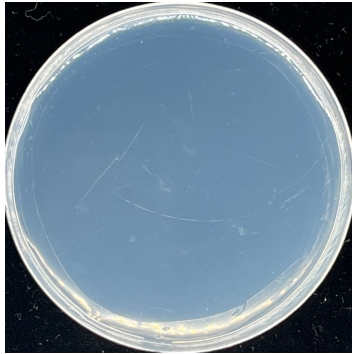

*pABAi-MdERF3p2*

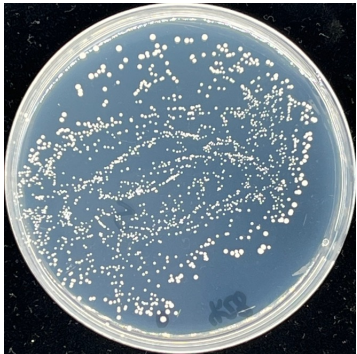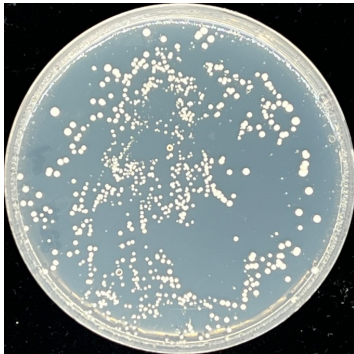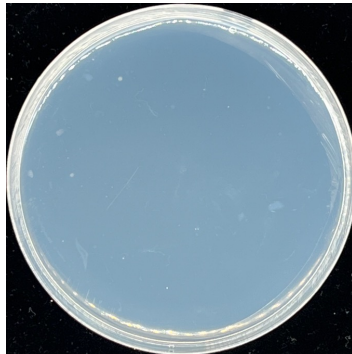

*pABAi-MdACS1*

Supplement: Web_Material_uhae284 [file web_material_uhae284.zip › Figure S11.pdf]

**A**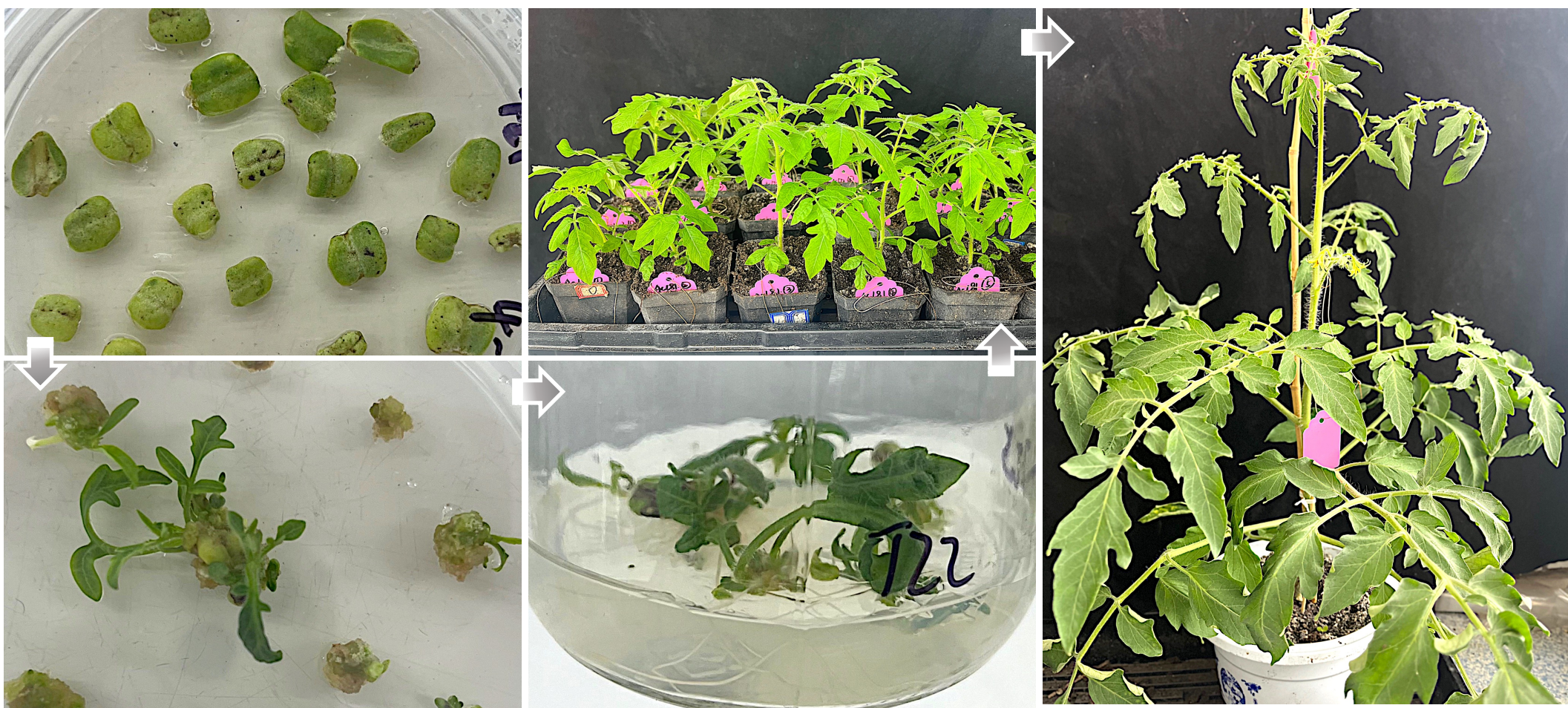**B**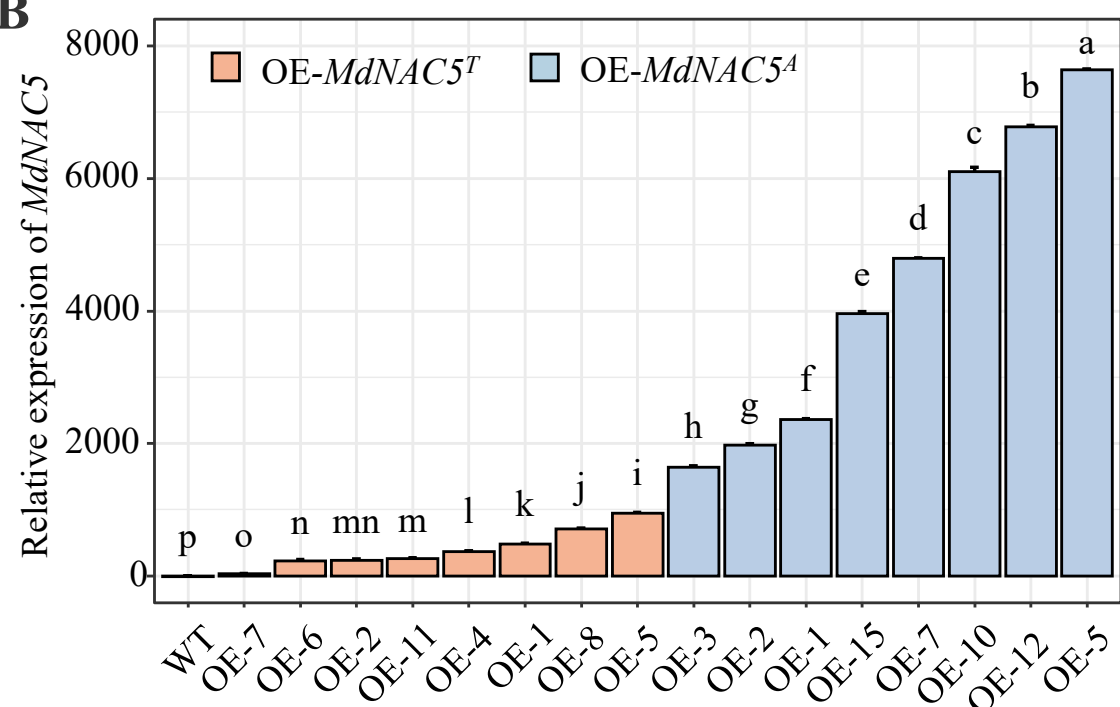**C**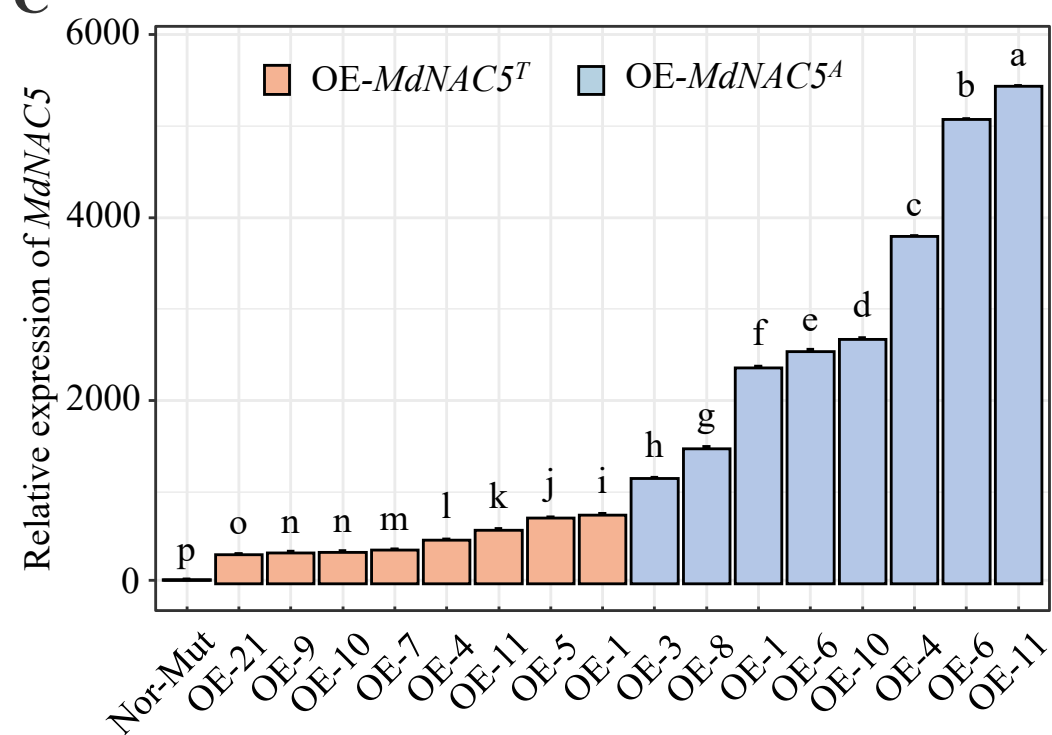**D**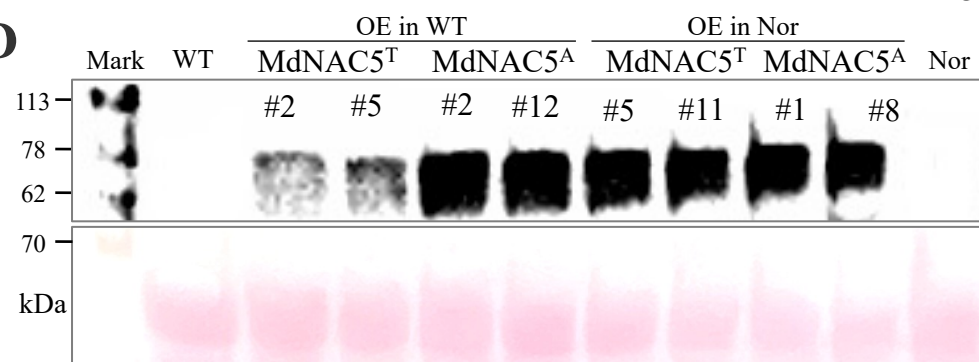

Supplement: Web_Material_uhae284 [file web_material_uhae284.zip › Figure S12.pdf]

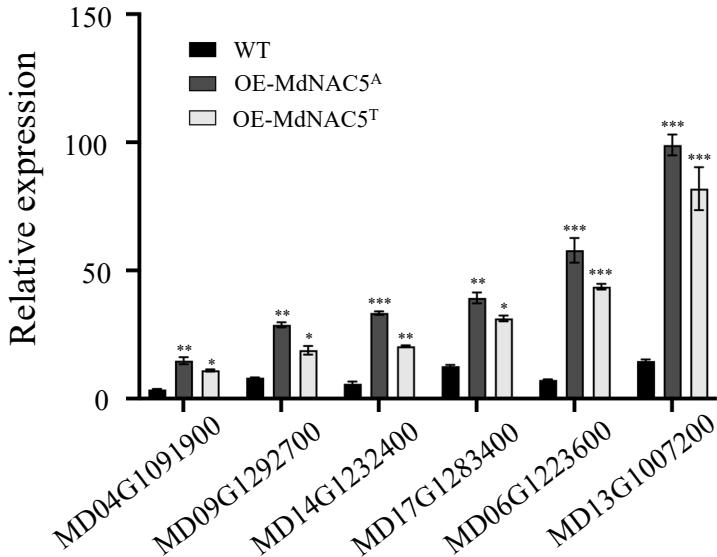

Supplement: Web_Material_uhae284 [file web_material_uhae284.zip › Figure S13.pdf]

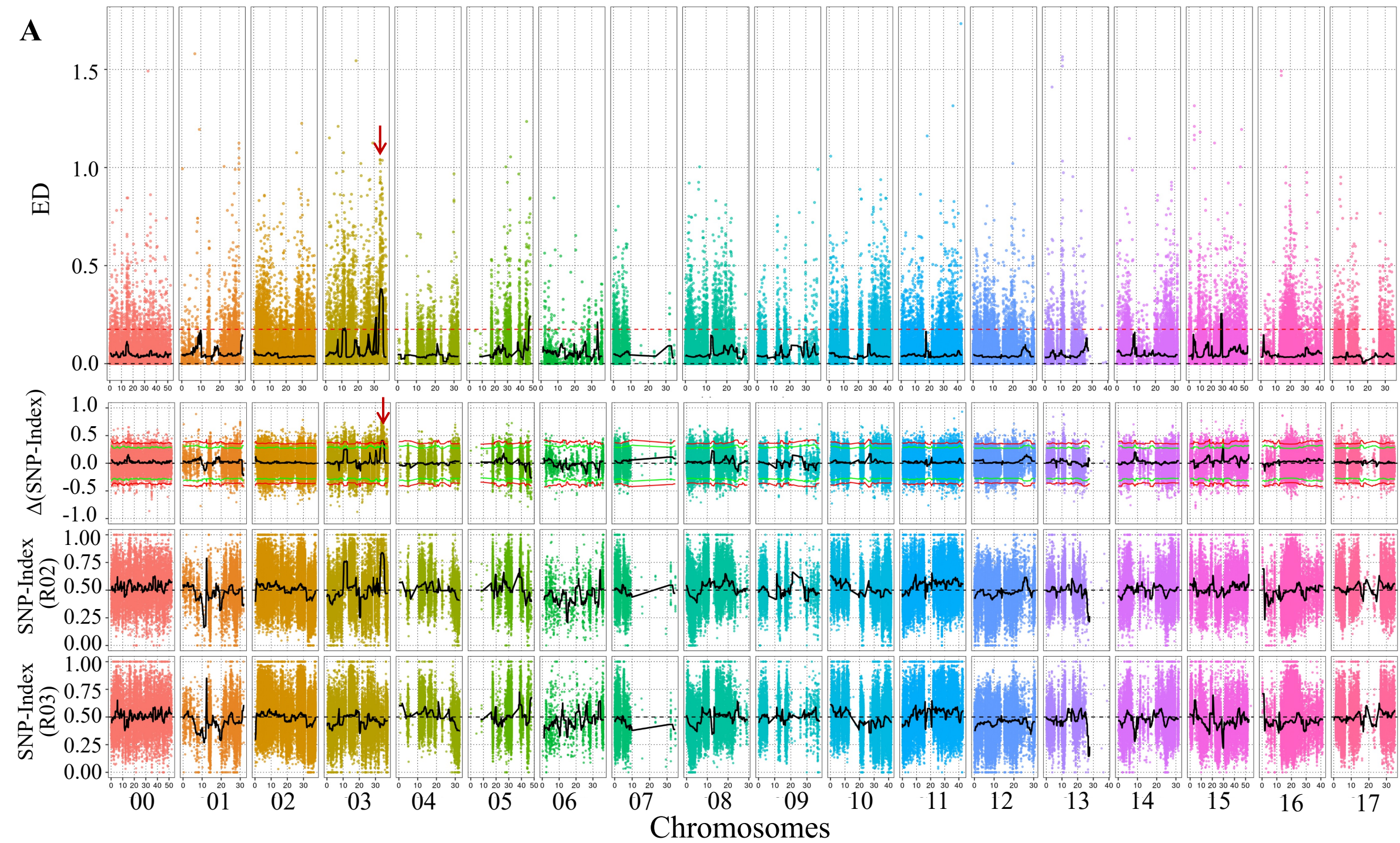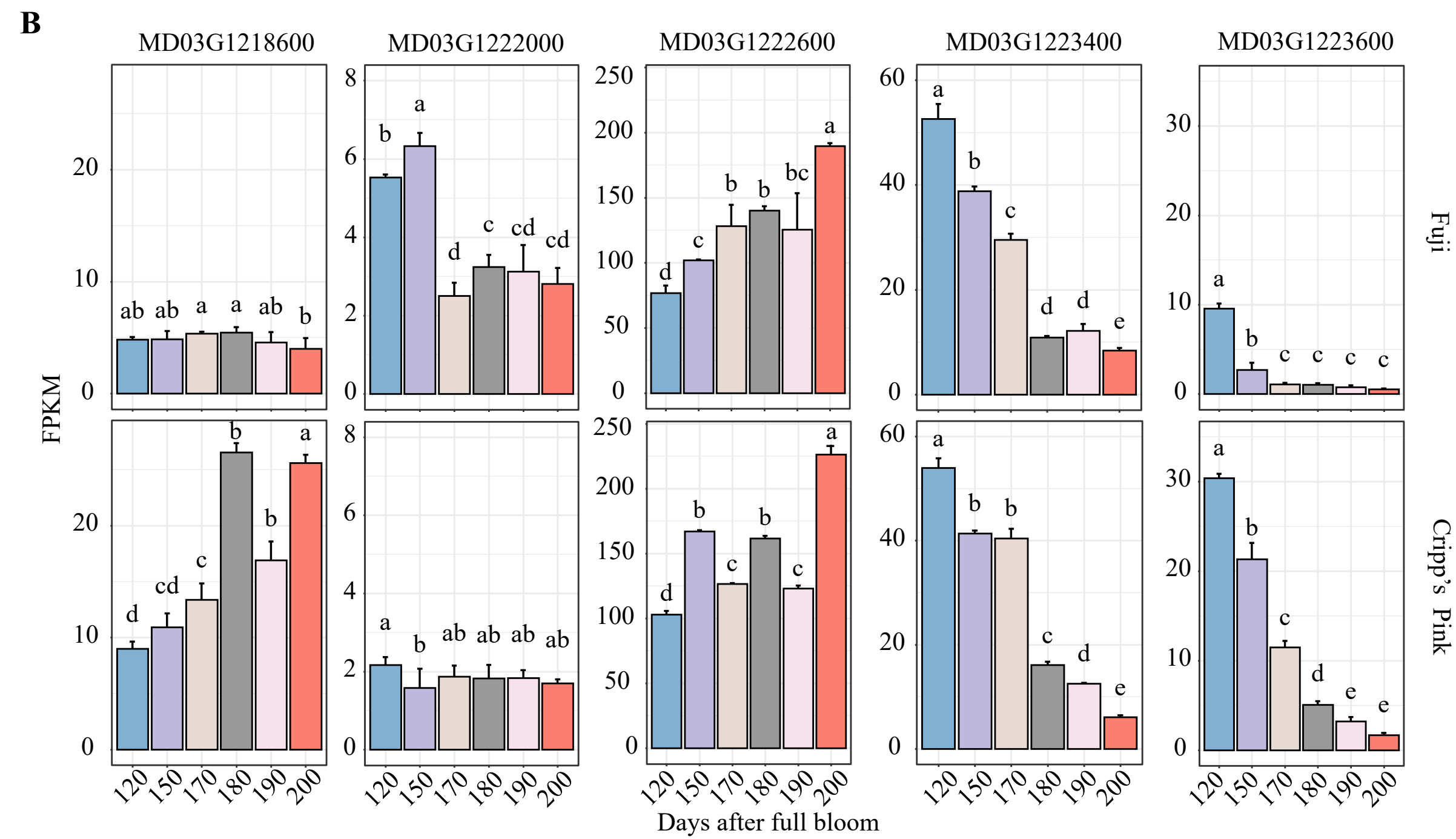

Supplement: Web_Material_uhae284 [file web_material_uhae284.zip › Figure. S7.pdf]
